# Supplementary material for: Dereplication, Annotation, and Characterization of 74 Potential Antimicrobial Metabolites from Penicillium Sclerotiorum Using t-SNE Molecular Networks
Source: Metabolites. 2021 Jul 8;11(7):444. doi: 10.3390/metabo11070444 (PMC8303670; doi:10.3390/metabo11070444)
Supplement: Supplementary file 1 [file metabolites-11-00444-s001.zip › metabolites-1276052-supplementary.pdf]

Dereplication, annotation and characterization of 74 potential anti-microbial metabolites from *Penicillium sclerotiorum* using t-SNE Molecular Networking

Teo Hebra, Salomé Poyer, Nicolas Elie, Elsa Van Elslande, David Touboul*, Véronique Eparvier*
Université Paris-Saclay, CNRS, Institut de Chimie des Substances Naturelles, UPR 2301, 91198, Gif-sur-Yvette, France.

SupData

Contents

Figure S1: Biological analyses of 109 ethyl acetate crude extracts from the associated microorganisms of French Guiana Termites....5
Figure S2: Molecular network from 109 crude extracts. 6
Figure S3: MS/MS information of compound **1**....7
Figure S4: MS/MS information of compound **2**....8
Figure S5: MS/MS information of compound **3**....9
Figure S6: MS/MS information of compound **4**....10
Figure S7: MS/MS information of compound **5**....11
Figure S8: Minimal inhibitory concentration of fractions 2 to 11 against three human pathogens 12
Figure S9: MS/MS information of compound **6**....13
Figure S10: MS/MS information of compound **7**....14
Figure S11: MS/MS information of compound **8**....15
Figure S12: MS/MS information of compound **9**....16
Figure S13: MS/MS information of compound **10**....17
Figure S14: MS/MS information of compound **11**....18
Figure S15: MS/MS information of compound **12**....19
Figure S16: MS/MS information of compound **13**....20
Figure S17: MS/MS information of compound **14**....21
Figure S18: MS/MS information of compound **15**....22
Figure S19: MS/MS information of compound **16**....23
Figure S20: MS/MS information of compound **17**....24
Figure S21: MS/MS information of compound **18**....25
Figure S22: MS/MS information of compound **19**....26
Figure S23: MS/MS information of compound **20**....27
Figure S24: MS/MS information of compound **21**....28
Figure S25: MS/MS information of compound **22**....29
Figure S26: MS/MS information of compound **23**....30
Figure S27: MS/MS information of compound **24**....31
Figure S28: MS/MS information of compound **25**....31
Figure S29: MS/MS information of compound **26**....32
Figure S30: MS/MS information of compound **27**....33

Figure S31: MS/MS information of compound **28**..........34
Figure S32: MS/MS information of compound **29**..........35
Figure S33: MS/MS information of compound **30**..........36
Figure S34: MS/MS information of compound **31**..........37
Figure S35: MS/MS information of compound **32**..........38
Figure S36: MS/MS information of compound **33**..........39
Figure S37: MS/MS information of compound **34**..........40
Figure S38: MS/MS information of compound **35**..........41
Figure S39: MS/MS information of compound **36**..........42
Figure S40: MS/MS information of compound **37**..........43
Figure S41: MS/MS information of compound **38**..........44
Figure S42: MS/MS information of compound **39**..........45
Figure S43: MS/MS information of compound **40**..........46
Figure S44: MS/MS information of compound **41**..........47
Figure S45: MS/MS information of compound **42**..........48
Figure S46: MS/MS information of compound **43**..........49
Figure S47: MS/MS information of compound **44**..........50
Figure S48: MS/MS information of compound **45**..........51
Figure S49: MS/MS information of compound **46**..........52
Figure S50: MS/MS information of compound **47**..........53
Figure S51: MS/MS information of compound **48**..........54
Figure S52: MS/MS information of compound **49**..........55
Figure S53: MS/MS information of compound **50**..........56
Figure S54: MS/MS information of compound **51**..........57
Figure S55: MS/MS information of compound **52**..........58
Figure S56: MS/MS information of compound **53**..........59
Figure S57: MS/MS information of compound **54**..........60
Figure S58: MS/MS information of compound **55**..........61
Figure S59: MS/MS information of compound **56**..........62
Figure S60: MS/MS information of compound **57**..........63
Figure S61: MS/MS information of compound **58**..........64
Figure S62: MS/MS information of compound **59**..........65
Figure S63: MS/MS information of compound **60**..........66
Figure S64: MS/MS information of compound **61**..........67
Figure S65: MS/MS information of compound **62**..........68
Figure S66: MS/MS information of compound **63**..........69
Figure S67: MS/MS information of compound **64**..........70
Figure S68: MS/MS information of compound **65**..........71
Figure S69: MS/MS information of compound **66**..........72
Figure S70: MS/MS information of compound **67**..........73

Figure S71: MS/MS information of compound **68**......74
Figure S72: MS/MS information of compound **69**......75
Figure S73: MS/MS information of compound **70**......76
Figure S74: MS/MS information of compound **71**......77
Figure S75: MS/MS information of compound **72**......78
Figure S76: MS/MS information of compound **73**......79
Figure S77: MS/MS information of compound **74**......80
Figure S78: MS/MS information of compound **75**......81
Figure S79: MS/MS information of compound **76**......82
Figure S80: MS/MS information of compound **77**......83
Figure S81: MS/MS information of compound **78**......84
Figure S82: MS/MS information of compound **79**......85
TableS1: ^{1}H NMR spectroscopic data for compounds **1**, **2**, **5**, **23, 75, 63, 80 and 74**......86
Table S2: ^{13}C NMR spectroscopic data for compounds **1**, **2**, **5**, **23**, **63**, **74**, **75**, **80**......87
Figure S83: ^{1}H NMR spectrum ($CDCl_3$) of compound **1**......88
Figure S84: 13C NMR spectrum ($CDCl_3$) of compound **1**......89
Figure S85: HRMS of compound **1**......90
Figure S86: ^{1}H NMR spectrum ($CDCl_3$) of compound **2**......91
Figure 87: ^{13}C NMR spectrum ($CDCl_3$) of compound **2**......92
Figure 88: HRMS of compound **2**......93
Figure S89: ^{1}H NMR spectrum ($CDCl_3$) of compound **5**......94
Figure S90: ^{13}C NMR spectrum ($CDCl_3$) of compound **5**......95
Figure S91: HRMS of compound **5**......96
Figure S92: ^{1}H NMR (MeOD) spectrum of compound compound **23**......97
Figure S93: HRMS of compound **23**......98
Figure S94: ^{1}H NMR spectrum ($CDCl_3$) of compound **75**......99
Figure S95: ^{13}C NMR ($CDCl_3$) spectrum of compound **75**......100
Figure S96: HRMS of compound **75**......101
Figure S97: ^{1}H NMR spectrum ($CDCl_3$) of compound **63**......102
Figure S98: ^{13}C NMR spectrum ($CDCl_3$) of compound **63**......103
Figure S99: COSY NMR spectrum ($CDCl_3$) of compound **63**......104
Figure S100: HSQC NMR spectrum ($CDCl_3$) of compound **63**......105
Figure S101: HMBC NMR spectrum ($CDCl_3$) of compound **63**......106
Figure S102: HRMS of compound **63**......107
Figure S103: ^{1}H NMR spectrum ($CDCl_3$) of compound **74**......108
Figure S104: ^{13}C NMR spectrum ($CDCl_3$) of compound **74**......109
Figure S106: HSQC NMR spectrum ($CDCl_3$) of compound **74**......111
Figure S107: HMBC NMR spectrum ($CDCl_3$) of compound **74**......112
Figure S108: HRMS of compound **74**......113
Figure S109: Compound **80** position within t-SNE molecular network......114

Figure S110: MS/MS information of compound **80**....115
Figure S111: ^{1}H NMR spectrum (DMF-*d6*) of compound **80**....116
Figure S112: ^{13}C NMR spectrum (DMF-*d6*) of compound **80**....117
Figure S113: COSY NMR spectrum (DMF-*d6*) of compound **80**....118
Figure S114: HSQC NMR spectrum (DMF-*d6*) of compound **80**....119
Figure S115: HMBC NMR spectrum (DMF-d6) of compound **80**....120
Figure S116: HRMS of compound **80**....121
FigS117: Structural elucidation of isolated azaphilones....121
Figure S118: UV spectra in acetonitrile of isolated azaphilones **1**, **2**, **5**, **23**, **63**, **74**, **75** and **80** 122
Figure S119: UV spectra in methanol of isolated azaphilones **1**, **2**, **5**, **23**, **63**, **74**, **75** and **80**...122
Figure S120: CD spectra of isolated azaphilones **2**, **5**, **23**, **63**, **74**, **75** and **80**....123
Figure S121: Absolute configuration of compound 1 and 5....123
Table S3**:** Crystal data and structure refinement....124
Table S4: Fractional Atomic Coordinates (×10^4) and Equivalent Isotropic Displacement Parameters ($Å^2$×10^3) for compound **1**....125
Table S5: Anisotropic Displacement Parameters (×10^4) for compound **1**....126
Table S6: Bond Lengths in Å for compound **1**....127
Table S7: Bond Angles in for compound **1**.....128
Table S8: Torsion Angles in ˚ for compound **1**....129
Table S9: Hydrogen Bonds for compound **1**....130
Table S10: Hydrogen Fractional Atomic Coordinates (×10^4) and Equivalent Isotropic Displacement Parameters ($Å^2$×10^3) for compound **1**....131
Table S11: Fractional Atomic Coordinates (×10^4) and Equivalent Isotropic Displacement Parameters ($Å^2$×10^3) for compound **5**....132
Table S12: Anisotropic Displacement Parameters (×10^4) for compound **5**....133
Table S13: Bond Lengths in Å for compound **5**.....134
Table S14: Bond Angles in ˚ for compound **5**.....134
Table S15: Torsion Angles in ° for compound **5**....135
Table S16: Hydrogen Bonds for compound **5**....135
Table S17 Hydrogen fractional atomic coordinates (×104) and equivalent isotropic displacement parameters (Å2×103) for compound **5**.....136
Figure S122: Evolution of retention time of acylated azaphilone in function of the length of acylation....137

Figure S1: Biological analyses of 109 ethyl acetate crude extracts from the associated microorganisms of French Guiana Termites. (a) Minimum inhibitory concentration (MIC) of crude extracts against 3 human pathogens, Methicillin-Resistant *Staphylococcus aureus* (bacteria), *Candida albicans* (yeast) and *Tricophyton rubrum* (fungus). (b) Percent of survival of MRC5 cell line (human fetal cell lung) from crude extracts. Red dots are value from *P. sclerotiorum* SNB-CN111 crude extract.

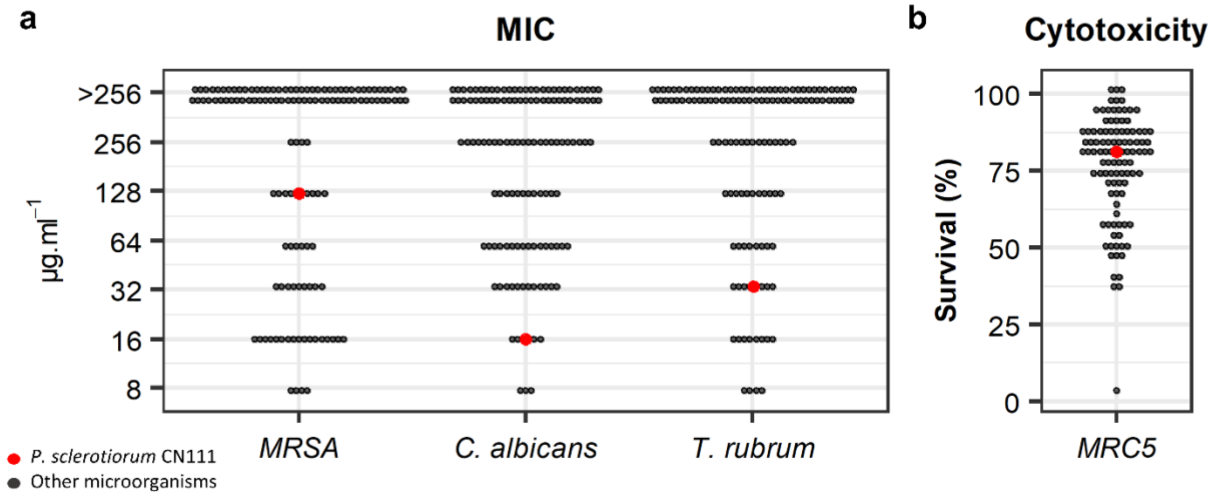

Figure S2: Molecular network from 109 crude extracts. Highlights of *P. sclerotiorum* unique molecular features (red), shared with other extracts (blue) or not present (gray)

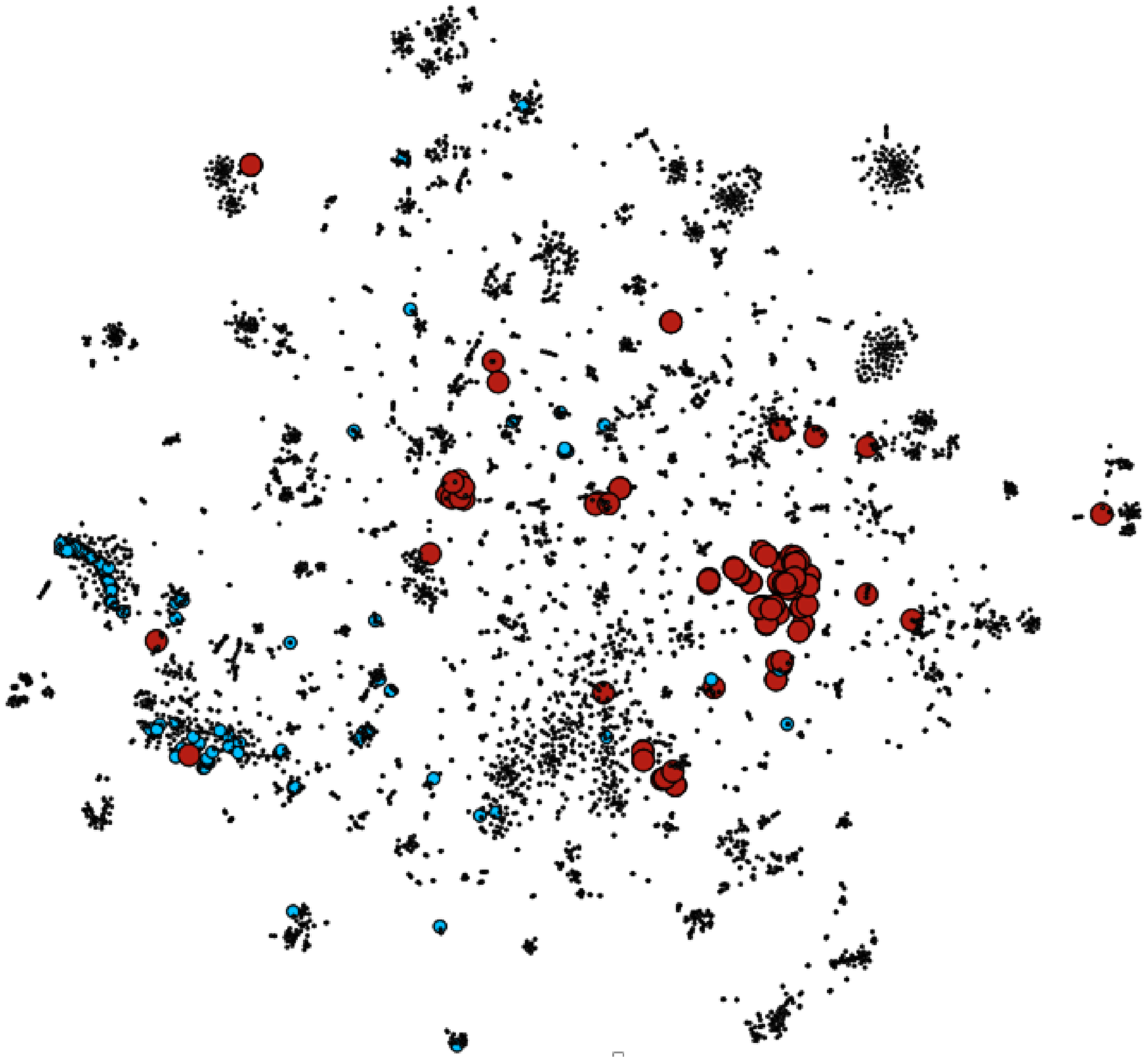

Figure S3: MS/MS information of compound **1** (*m/z* 391.1309, −0.6 ppm, cosine score= 0.82, level 0) from molecular network.(a) MS/MS spectrum of compound **1**, (b) the mirror plot of MS/MS spectra from compound **1** against its standard spectrum from MS-Dial library, and (c) the fragment list and their contribution to cosine score.

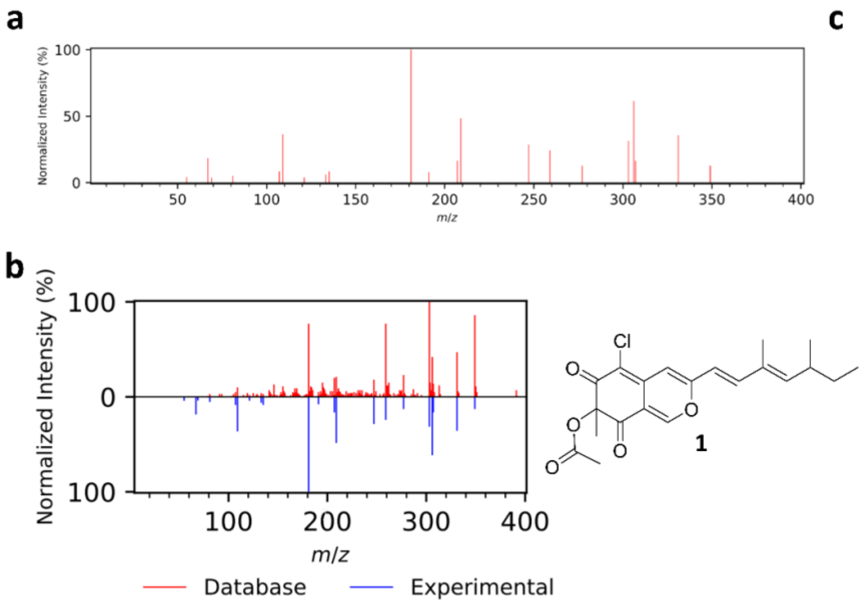

c

Fragment	Score
81.0693	0.005
107.0857	0.0081
109.1015	0.0238
121.101	0.0035
133.1012	0.0062
135.1169	0.0063
181.0055	0.1092
191.0303	0.0078
207.021	0.0226
209.0007	0.0397
247.0522	0.0282
259.0525	0.0538
277.0631	0.0214
303.1153	0.0698
306.1022	0.0632
307.1087	0.0189
331.1106	0.051
349.1213	0.0414

Figure S4: MS/MS information of compound **2** (*m/z* 390.1472, −1.4 ppm, cosine score= 0.97, level 0) from molecular network. (a) MS/MS spectrum of compound **2**, (b) the mirror plot of MS/MS spectra from compound **2** against its standard spectrum from MONA library, and (c) the fragment list and their contribution to cosine score.

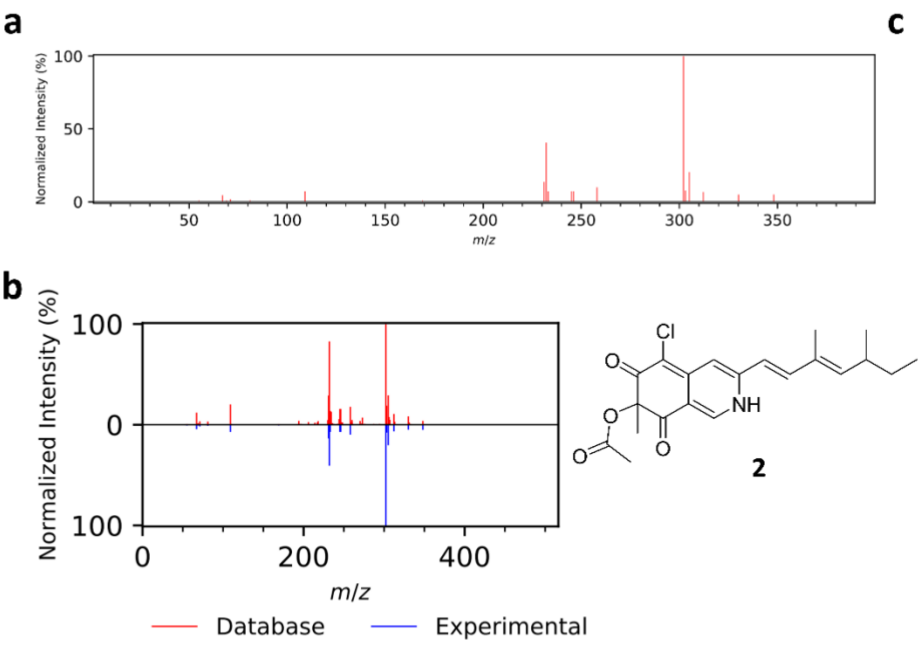

c

Fragment	Score
55.0546	0.0019
67.0544	0.0203
69.07	0.003
71.0856	0.0066
81.07	0.0051
110.1046	0.0015
169.0887	0.0017
231.0447	0.0554
232.0526	0.1622
233.0582	0.0277
245.0604	0.0295
246.0682	0.0295
258.0683	0.0369
302.1308	0.2797
303.1342	0.0339
305.1177	0.0681
312.1594	0.0235
330.1255	0.0178
348.1362	0.0123

Figure S5: MS/MS information of compound **3** (*m/z* 383.1859, −1.6 ppm, cosine score= 0.86, level 2) from molecular network. (a) MS/MS spectrum of compound **3**, (b) the mirror plot of MS/MS spectra from compound **3** against its standard spectrum from MS-Dial library, and (c) the fragment list and their contribution to cosine score.

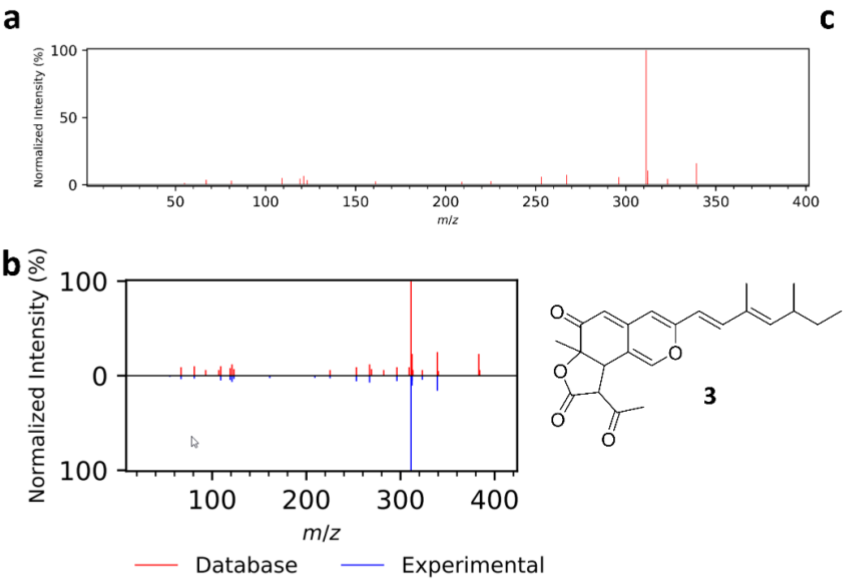

c

Fragment	Score
67.0544	0.023
81.0699	0.0218
109.1013	0.0284
119.0857	0.0242
121.1013	0.0357
123.1169	0.0196
225.1275	0.0162
253.1224	0.0293
267.1383	0.0375
296.177	0.0287
311.2006	0.4012
312.1729	0.0248
312.2039	0.0622
323.1642	0.0204
339.1228	0.08
67.0544	0.023
81.0699	0.0218
109.1013	0.0284

Figure S6: MS/MS information of compound **4** (*m/z* 417.1456, 1.8 ppm, cosine score= 0.74, level 2) from molecular network. (a) MS/MS spectrum of compound **4**, (b) the mirror plot of MS/MS spectra from compound **4** against its standard spectrum from MS-Dial library, and (c) the fragment list and their contribution to cosine score.

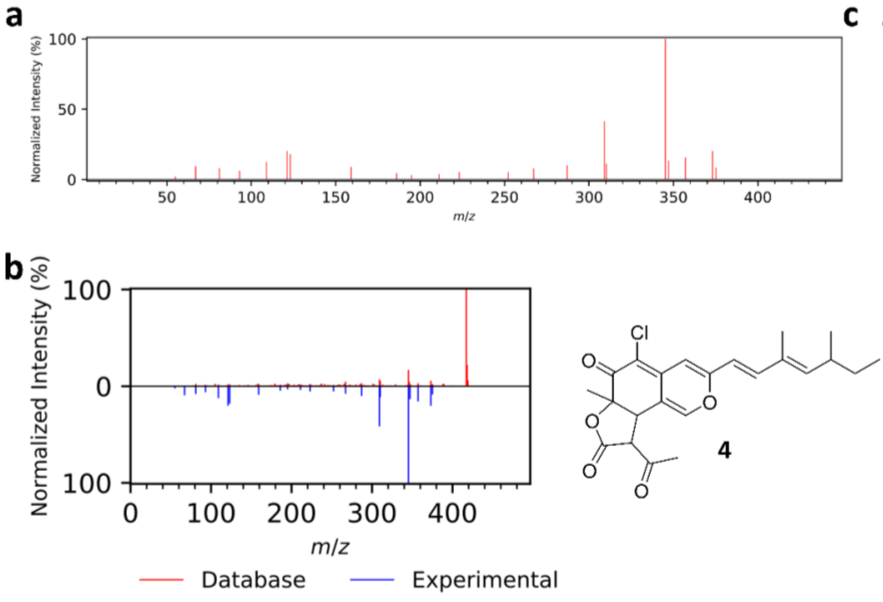

Fragment	Score
81.0691	0.0111
93.0702	0.0074
109.0999	0.0119
121.1049	0.0156
123.1196	0.015
159.0827	0.0115
186.0635	0.0065
195.0609	0.0052
211.1095	0.0064
223.1081	0.0086
252.1083	0.0061
267.1752	0.0156
287.0815	0.0139
309.1843	0.0435
310.1903	0.0195
345.1631	0.1034
347.1527	0.0134
357.1263	0.0175
373.081	0.0272
375.1372	0.0108
81.0691	0.0111
93.0702	0.0074

Figure S7: MS/MS information of compound **5** (*m/z* 434.1731, −0.5 ppm, cosine score= 0.83, level 0) from molecular network. (a) MS/MS spectrum of compound **5**, (b) the mirror plot of MS/MS spectra from compound **5** against its standard spectrum from MS-Dial library, and (c) the fragment list and their contribution to cosine score.

a

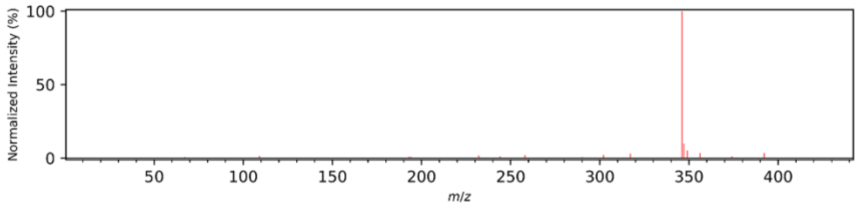

b

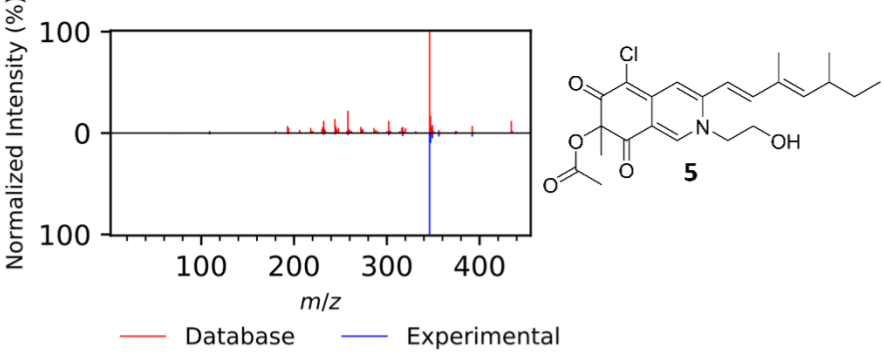

c

Fragment	Score
109.0649	0.0032
109.1015	0.0075
193.0295	0.0118
194.0366	0.0093
232.0528	0.0207
244.0529	0.019
258.069	0.0289
290.0942	0.0056
302.1299	0.0126
317.1179	0.0189
346.1587	0.4463
347.1617	0.0576
349.1452	0.0285
356.1872	0.0143
374.1536	0.0069
392.1644	0.0221

Figure S8: Minimal inhibitory concentration of fractions 2 to 11 against three human pathogens (a) MRSA, (b) *C. albicans*, and (c)*T. rubrum*.

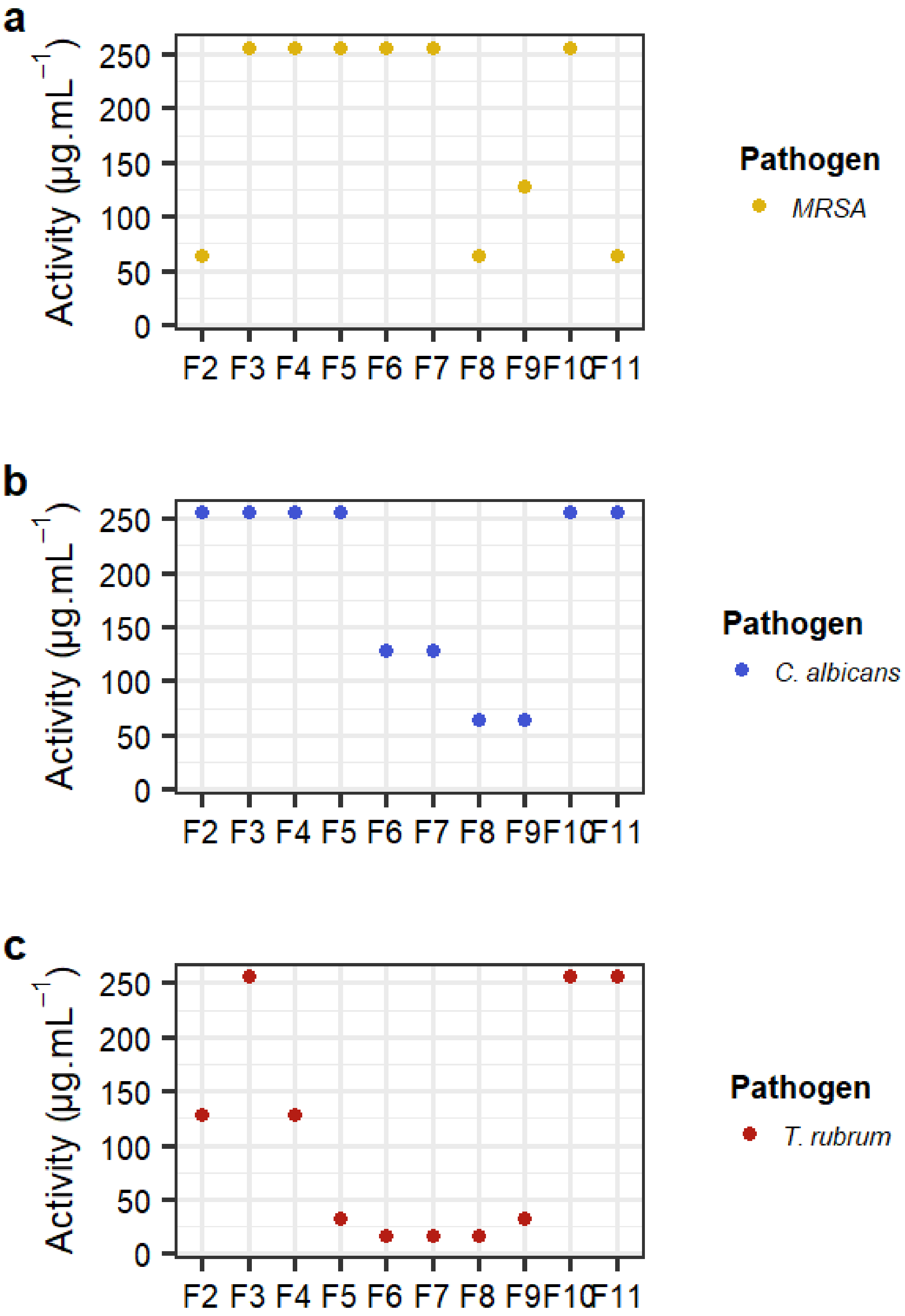

Figure S9: MS/MS information of compound **6** (*m/z* 476.1846, −2.4 ppm, cosine score= 0.92, level 2) from molecular network. (a) MS/MS spectrum of compound **6**, (b) the mirror plot of MS/MS spectra from compound **6** against its standard spectrum from MONA library, and (c) the fragment list and their contribution to cosine score.

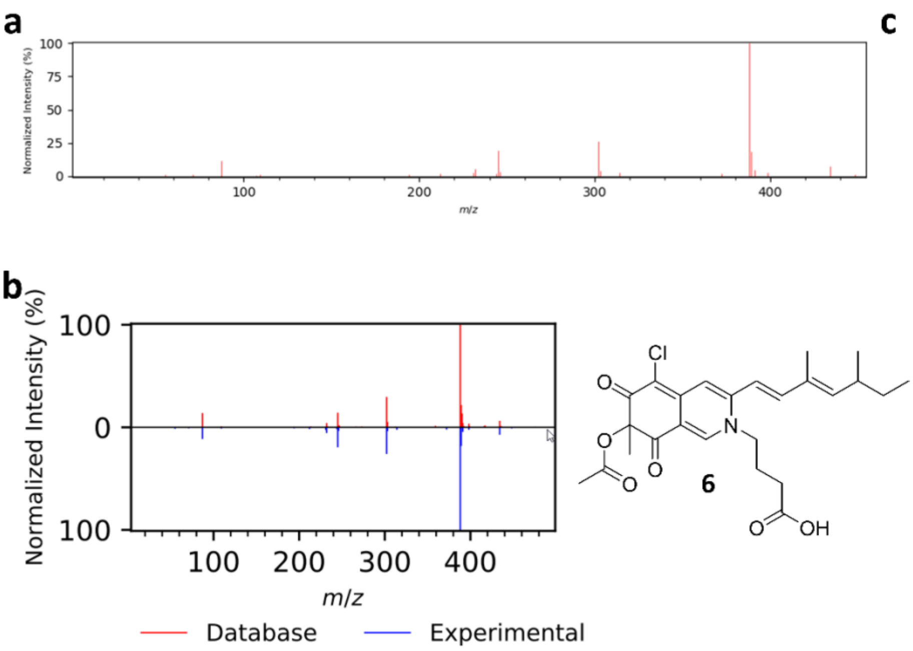

c

Fragment	Score
71.0862	0.0009
87.0446	0.0547
109.1015	0.0048
194.0368	0.0026
231.0444	0.0051
232.0523	0.0208
244.0527	0.0036
245.06	0.0721
246.0641	0.0114
302.1304	0.1201
303.1339	0.0192
388.1672	0.433
389.1704	0.0861
391.1543	0.0186
398.1962	0.0124
434.1727	0.0295

Figure S10: MS/MS information of compound **7** (*m/z* 385.1426, −3.5 ppm, cosine score = 0.76, level 2) from molecular network. (a) MS/MS spectrum of compound **7**, (b) the mirror plot of MS/MS spectra from compound **7** against its standard spectrum from GNPS library, and (c) the fragment list and their contribution to cosine score.

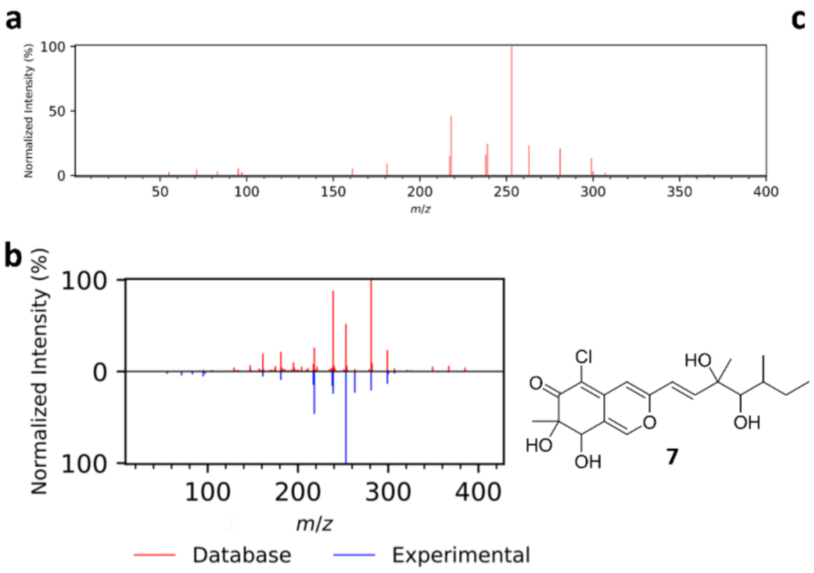

c

Fragment	Score
83.0483	0.0039
95.0492	0.0074
161.0595	0.0242
181.0064	0.0329
217.0862	0.0255
218.0942	0.0809
238.0409	0.0181
239.0482	0.1082
253.0638	0.1681
263.0482	0.0187
281.059	0.1062
299.0696	0.0412
300.0714	0.0042
307.1122	0.0064
367.1318	0.0052
83.0483	0.0039
95.0492	0.0074
161.0595	0.0242
181.0064	0.0329

Figure S11: MS/MS information of compound **8** (*m/z* 443.2438, −2.3 ppm, cosine score= 0.96, level 2) from molecular network. (a) MS/MS spectrum of compound **8**, (b) the mirror plot of MS/MS spectra from compound **8** against its standard spectrum from MONA library, and (c) the fragment list and their contribution to cosine score.

a

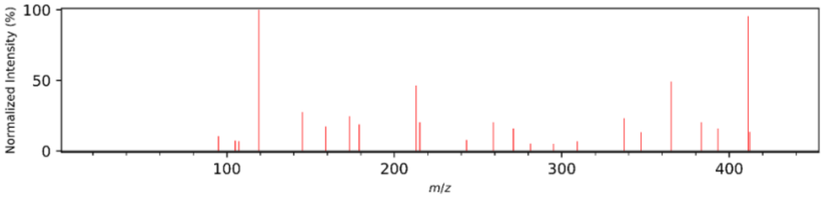

b

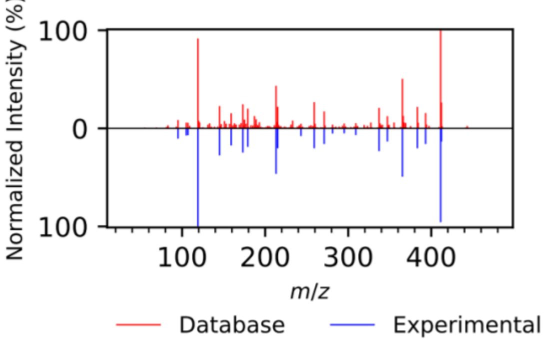

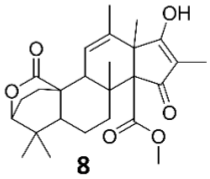

8

c

Fragment	Score
95.0855	0.0124
105.0699	0.0085
107.0855	0.0083
119.0855	0.126
145.1011	0.0329
159.1168	0.0216
173.1325	0.0324
179.0702	0.0256
213.1638	0.0591
215.1794	0.0278
243.1378	0.008
259.169	0.0307
271.1328	0.0219
281.1898	0.0059
295.1328	0.0066
309.1847	0.0079
337.2161	0.029
347.2006	0.017
365.2109	0.0657
383.2216	0.0277
393.2056	0.0208
411.2162	0.1287
412.2198	0.0248

Figure S12: MS/MS information of compound **9** (*m/z* 245.1292, −3.0 ppm, cosine score= 0.97, level 2) from molecular network. (a) MS/MS spectrum of compound **9**, (b) the mirror plot of MS/MS spectra from compound **9** against its standard spectra from compound GNPS library, and (c) the fragment list and their contribution to cosine score.

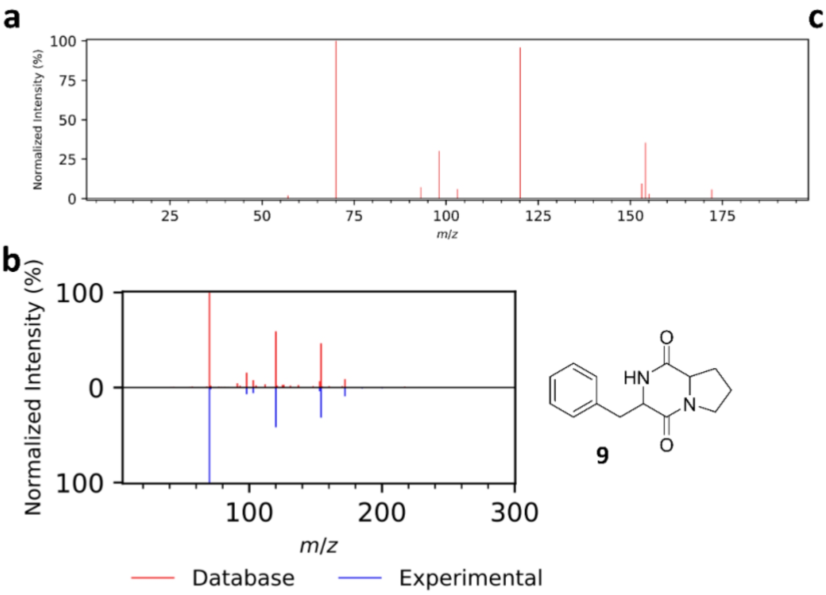

c

Fragment	Score
70.0652	0.4062
71.0689	0.007
98.0595	0.0423
103.0538	0.0275
120.0804	0.2017
153.0649	0.0208
154.0729	0.1559
172.1115	0.0367
200.1025	0.0022

Figure S13: MS/MS information of compound **10** (*m/z* 279.2321, −0.9 ppm, cosine score= 0.88, level 2) from molecular network. (a) MS/MS spectrum of compound **10**, (b) the mirror plot of MS/MS spectra from compound **10** against its standard spectra from compound NIST14 library, and (c) the fragment list and their contribution to cosine score.

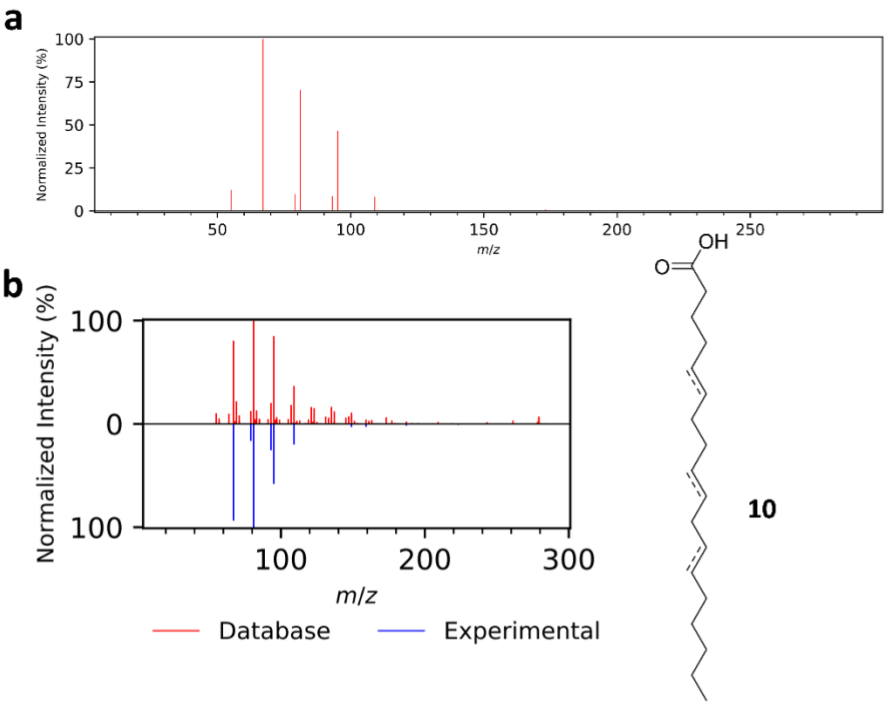

c

Fragment	Score
67.0548	0.1809
79.0547	0.0299
81.0703	0.2084
93.0702	0.0473
95.0858	0.1464
109.1014	0.0565
149.0231	0.0119
159.1166	0.0073
187.1485	0.0039

Figure S14: MS/MS information of compound **11** (*m/z* 281.2484, −3.2 ppm, cosine score= 0.75, level 2) from molecular network. (a) MS/MS spectrum of compound **11**, (b) the mirror plot of MS/MS spectra from compound **11** against its standard spectra from compound MONA library, and (c) the fragment list and their contribution to cosine score.

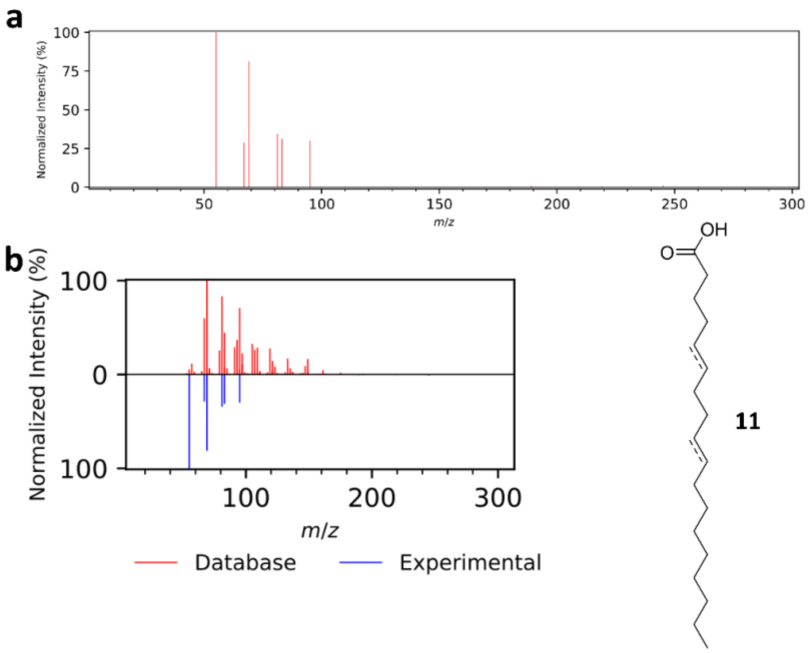

c

Fragment	Score
69.0699	0.1806
81.0699	0.1072
95.0855	0.0923
67.0542	0.0833
83.0855	0.0748
55.0543	0.0464
69.0699	0.1806
81.0699	0.1072
95.0855	0.0923

Figure S15: MS/MS information of compound **12** (*m/z* 391.2827, 4.1 ppm, cosine score= 0.97, level 2) from molecular network.(a) MS/MS spectrum of compound **12**, (b) the mirror plot of MS/MS spectra from compound **12** against its standard spectrum from GNPS library, and (c) the fragment list and their contribution to cosine score.

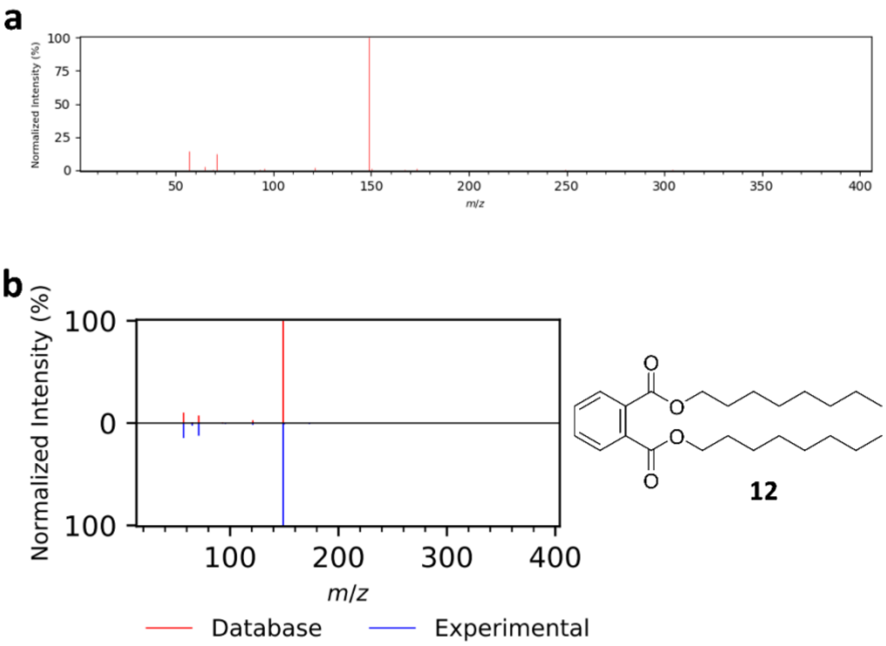

c

Fragment	Score
57.0698	0.094
65.0383	0.0113
71.0858	0.0731
93.0341	0.0049
121.0287	0.017
149.0246	0.7661
150.0268	0.0042
167.0338	0.0011
173.0996	0.0011

Figure S16: MS/MS information of compound **13** (*m/z* 371.1016, −1.0 ppm, cosine score= 0.95, level 2) from molecular network. (a) MS/MS spectrum of compound **13**, (b) the mirror plot of MS/MS spectra from compound **13** against its standard spectra from compound NIST14 library, and (c) the fragment list and their contribution to cosine score.

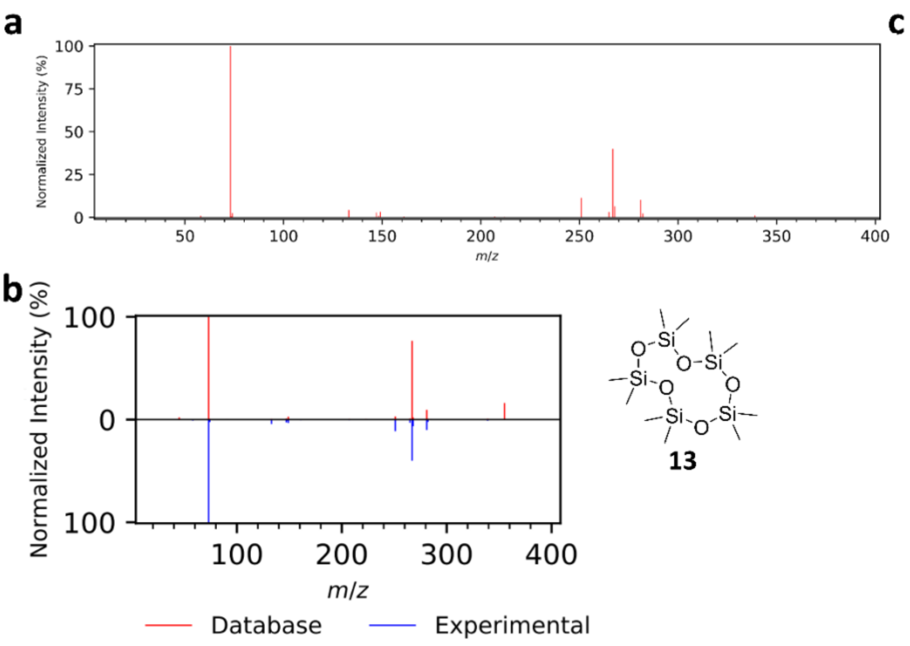

c

Fragment	Score
58.0234	0.0021
73.0467	0.4803
73.3424	0.0009
74.0482	0.0056
133.0138	0.0071
147.0639	0.0079
148.0615	0.002
149.044	0.0147
207.0313	0.0023
250.9665	0.0281
265.019	0.0091
266.9992	0.2659
267.9991	0.0177
281.0509	0.047
282.0524	0.0051
339.038	0.0043

Figure S17: MS/MS information of compound **14** (*m/z* 351.1807, −1.4 ppm level 2) from molecular network and isotopic pattern of **14**. (a) MS/MS spectrum of compound **14**, (b) the mirror plot of MS/MS spectra from compound **14** against itself with the main contributors to cosine score (bold), and (c) the fragment list and their contribution to cosine score.

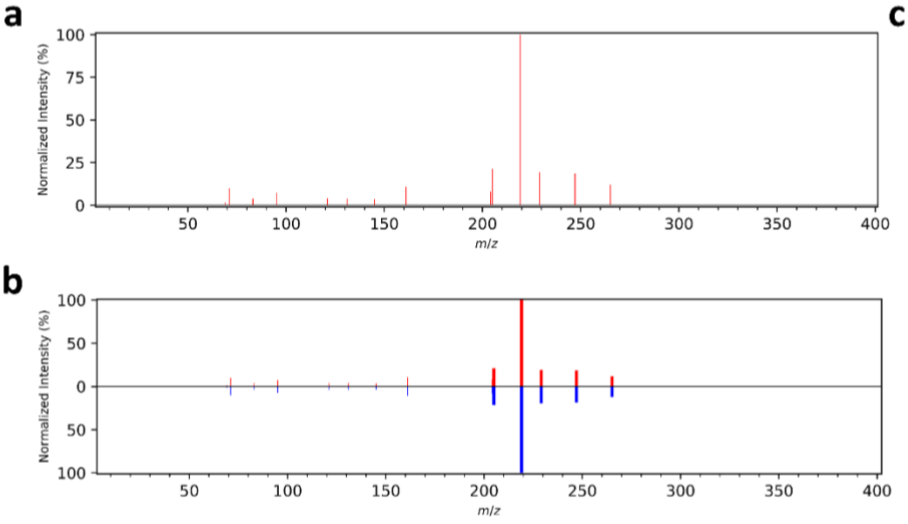

c

Fragment	Score
69.034	0.008
71.0498	0.0444
83.0492	0.0175
95.0495	0.0325
121.0649	0.018
131.0854	0.0175
145.066	0.0157
161.0603	0.0473
204.0779	0.0355
**205.0865**	**0.0947**
**219.1018**	**0.4437**
**229.0866**	**0.0858**
**247.0966**	**0.0828**
**265.1066**	**0.0532**
333.1684	0.0009

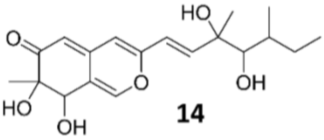

14

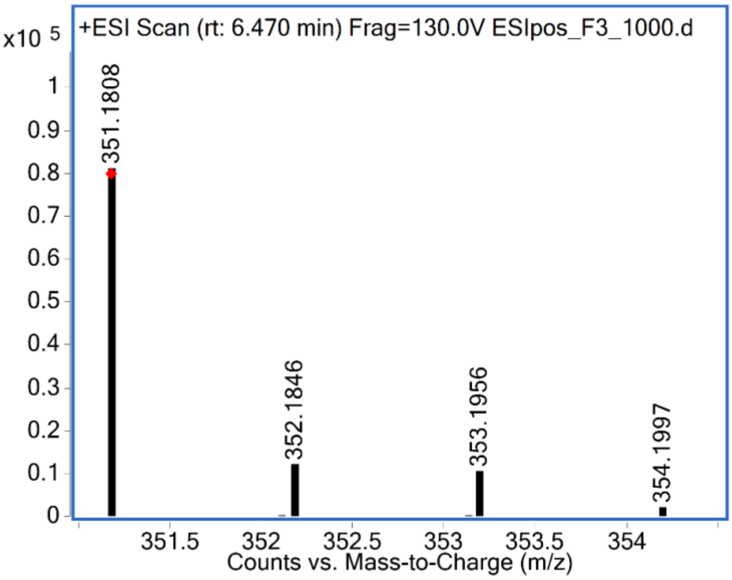

Annotation explanation: Its *m/z* difference with compound **7** is 33.9608, and it isotopic pattern lack of typical ^{37}Cl isotopic peak.

Figure S18: MS/MS information of compound **15** (*m/z* 417.1914, −1.5 ppm, level 2) from molecular network. (a) MS/MS spectrum of compound **15**, (b) the mirror plot of MS/MS spectra from compound **15** against itself with the main contributors to cosine score (bold), and (c) the fragment list and their contribution to cosine score.

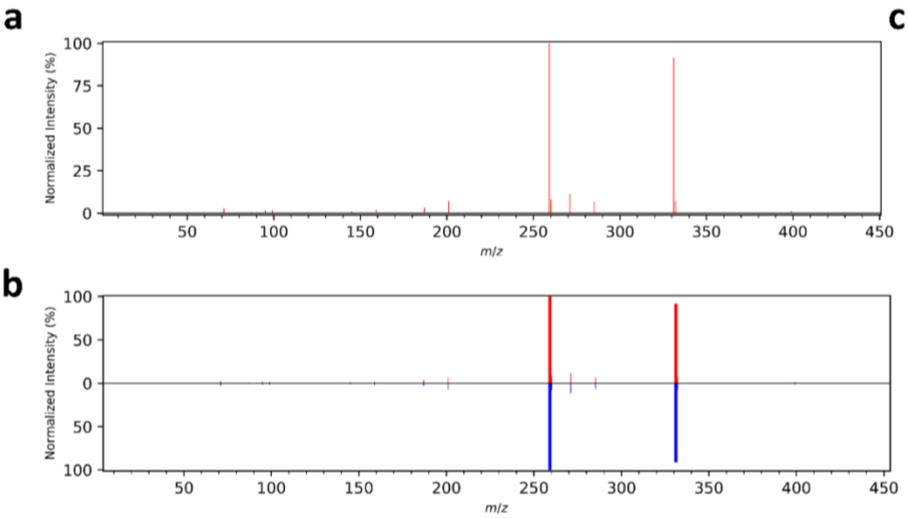

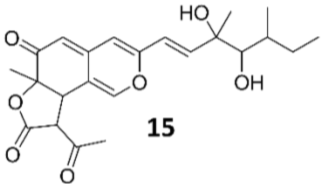

15

c

Fragment	Score
57.0694	0.0011
69.0694	0.0029
71.0488	0.0109
87.08	0.0023
95.0486	0.0067
99.0433	0.0069
145.0635	0.004
159.0785	0.0083
187.0736	0.0125
201.0894	0.0276
**259.1307**	**0.3931**
260.1022	0.0251
260.1341	0.0326
271.0948	0.0452
285.1099	0.0268
**331.1149**	**0.3596**
332.1189	0.0284
399.1767	0.0051
400.1803	0.0007

Annotation explanation: Its presence within cluster C of diol-azaphilones (compounds **7** and **14**) and its exact mass corresponding to the one reported in the literature for *Penicillium sp.* [1].

Figure S19: MS/MS information of compound **16** (*m/z* 353.1963, −1.2 ppm, level 3) from molecular network. (a) MS/MS spectrum of compound **16**, (b) the mirror plot of MS/MS spectra from compound **16** against itself with the main contributors to cosine score (bold), and (c) the fragment list and their contribution to cosine score.

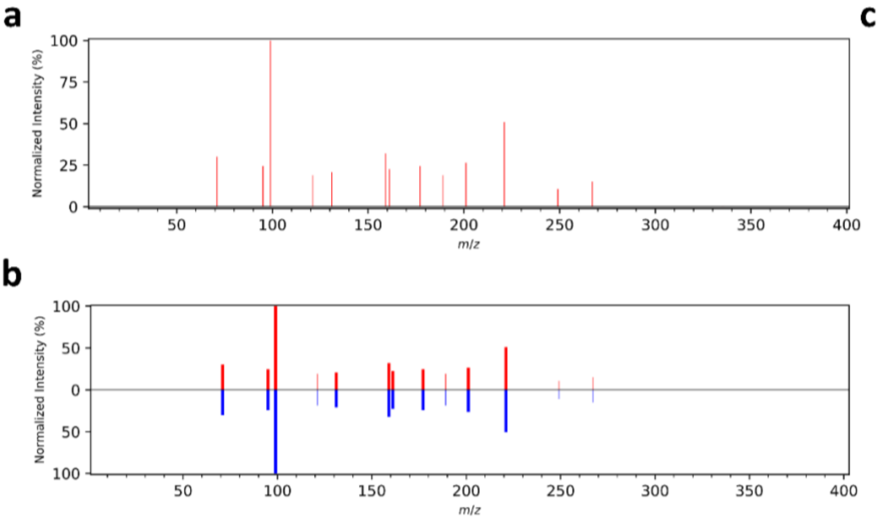

c

Fragment	Score
**71.0496**	**0.0757**
**95.0498**	**0.0615**
**99.0448**	**0.2509**
121.0653	0.0473
**131.0861**	**0.0521**
**159.0811**	**0.0805**
**161.0965**	**0.0568**
**177.0918**	**0.0615**
189.0918	0.0473
**201.0916**	**0.0663**
**221.118**	**0.1278**
249.1129	0.027
267.1235	0.0379
335.1854	0.0009

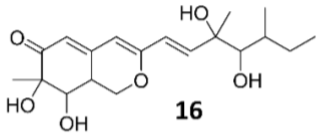

16

Annotation explanation: **16** presence within cluster C, its *m/z* difference of 2.0156 with compounds **7** and **14**, corresponding to a difference of H_2 (loss of an unsaturation) and its exact mass corresponding to the one reported in the literature for *Penicillium sp.* [1]

Figure S20: MS/MS information of compound **17** (*m/z* 387.1576, −1.8 ppm, level 2) from molecular network. (**a**) MS/MS spectrum of compound **17**, (**b**) the mirror plot of MS/MS spectra from compound **17** against itself with the main contributors to cosine score (bold), and (**c**) the fragment list and their contribution to cosine score.

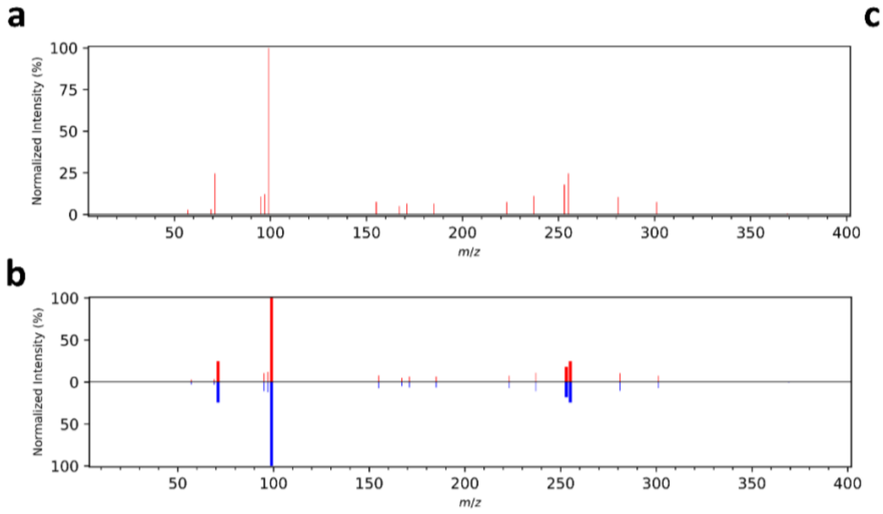

c

Fragment	Score
57.0704	0.0107
69.0707	0.012
**71.0501**	**0.0948**
95.0503	0.0411
97.0291	0.0468
**99.0452**	**0.3854**
155.0267	0.0291
167.0263	0.019
171.0216	0.0253
185.0377	0.0253
223.053	0.0284
237.0691	0.0423
**253.064**	**0.0695**
**255.08**	**0.0948**
281.0592	0.0404
301.0859	0.0284
369.1532	0.0021

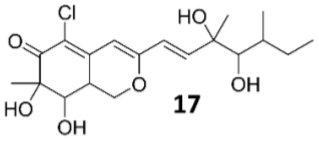

17

Annotation explanation: compound **17** presence within cluster C, its *m/z* difference of 2.0156 with compounds **7** and **14**, corresponding to a difference of H_2 (loss of an unsaturation), its exact mass corresponding to the one reported in the literature for *Penicillium sp.* [1]

Figure S21: MS/MS information of compound **18** (*m/z* 429.1675, 0.0 ppm, level 3) from molecular network. (a) MS/MS spectrum of compound **18**, (b) the mirror plot of MS/MS spectra from compound **18** against itself with the main contributors to cosine score (bold), and (c) the fragment list and their contribution to cosine score.

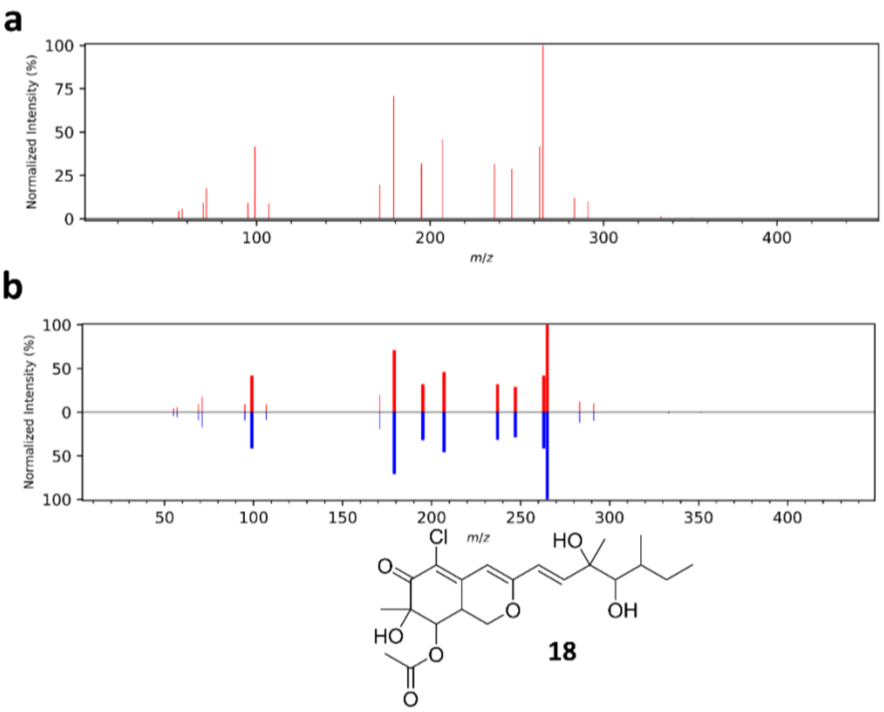

c

Fragment	Score
55.0543	0.0088
57.0703	0.0112
69.0703	0.0175
71.0496	0.0335
95.0494	0.0175
**99.0446**	**0.0797**
107.0861	0.0167
171.0208	0.0375
**179.0261**	**0.1355**
**195.0214**	**0.0614**
**195.0566**	**0.0558**
**207.0209**	**0.0877**
**237.0677**	**0.0606**
**247.0523**	**0.055**
**263.047**	**0.0797**
**265.0628**	**0.1913**
283.0735	0.0231
291.1149	0.0191
333.1261	0.0025
351.1375	0.0011
409.4811	0

Annotation explanation: The spatial proximity within the t-SNE with compounds **16** and **17** and its exact mass corresponding to the one reported in the literature for *Penicillium sp.* [1]**.**

Figure S22: MS/MS information of compound **19** (*m/z* 490.2001, −2.1 ppm, level 3) from molecular network. (**a**) MS/MS spectrum of compound **19**, (**b**) the mirror plot of MS/MS spectra from compound **19** against itself with the main contributors to cosine score (bold), and (**c**) the fragment list and their contribution to cosine score.

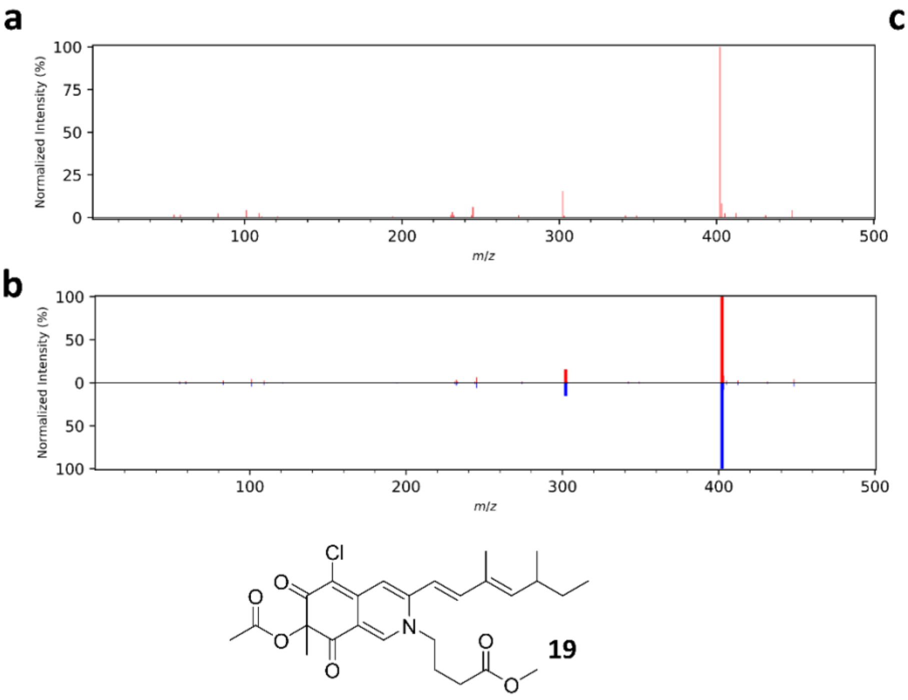

c

Fragment	Score
55.0538	0.0097
59.0485	0.0092
59.0593	0.0092
83.0498	0.0145
101.06	0.0259
109.1013	0.0156
111.0433	0.003
121.1014	0.0044
194.0353	0.0031
231.0434	0.0092
232.0524	0.0183
233.0611	0.0075
244.0527	0.0081
245.0597	0.0366
274.0965	0.0081
**302.1313**	**0.0916**
303.1325	0.0054
342.1606	0.0065
349.1472	0.0059
**402.1839**	**0.5924**
403.1881	0.0485
405.1707	0.0151
412.2101	0.0156
431.182	0.0075
448.1888	0.0248

Figure S23: MS/MS information of compound **20** (*m/z* 475.1989, 1.1 ppm, level 3) from molecular network. (**a**) MS/MS spectrum of compound **20**, (**b**) the mirror plot of MS/MS spectra from compound **20** against itself with the main contributors to cosine score (bold), and (**c**) the fragment list and their contribution to cosine score.

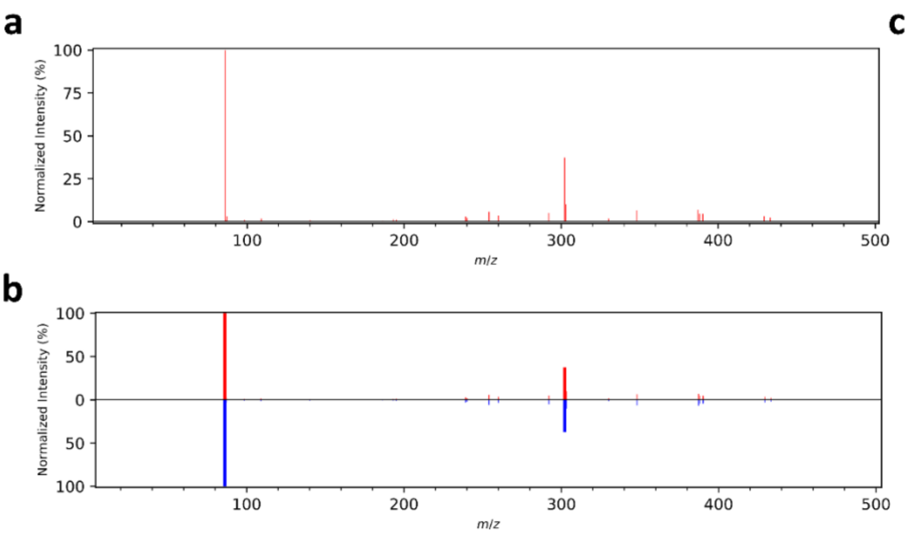

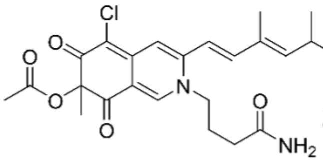

20

c

Fragment	Score
**86.0604**	**0.4621**
86.0999	0.005
86.3695	0.0025
87.0609	0.0139
98.4539	0.0046
109.1018	0.0075
140.1835	0.0036
186.3338	0.0023
193.1194	0.0057
195.0431	0.0052
239.1793	0.0135
240.0985	0.01
254.0832	0.0267
260.0472	0.0158
292.1302	0.0231
**302.1304**	**0.1724**
303.133	0.0462
330.1256	0.0078
348.1353	0.0302
387.1839	0.032
388.1301	0.0213
390.1415	0.0213
390.1688	0.0151
429.3014	0.0149
433.188	0.0107

Annotation explanation: Its *m/z* difference with compound **6** is 0.9857, corresponding to O/NH conversion. This *m/z* difference was also observed for the fragment corresponding to the carbon chain attached to the nitrogen (*m/z* 87.0438 for compound **6** and 86.0604 for compound **20**).

Figure S24: MS/MS information of compound **21** (*m/z* 395.1627, −1.8 ppm, level 3) from molecular network. (**a**) MS/MS spectrum of compound **21**, (**b**) the mirror plot of MS/MS spectra from compound **21** against itself with the main contributors to cosine score (bold), and (**c**) the fragment list and their contribution to cosine score.

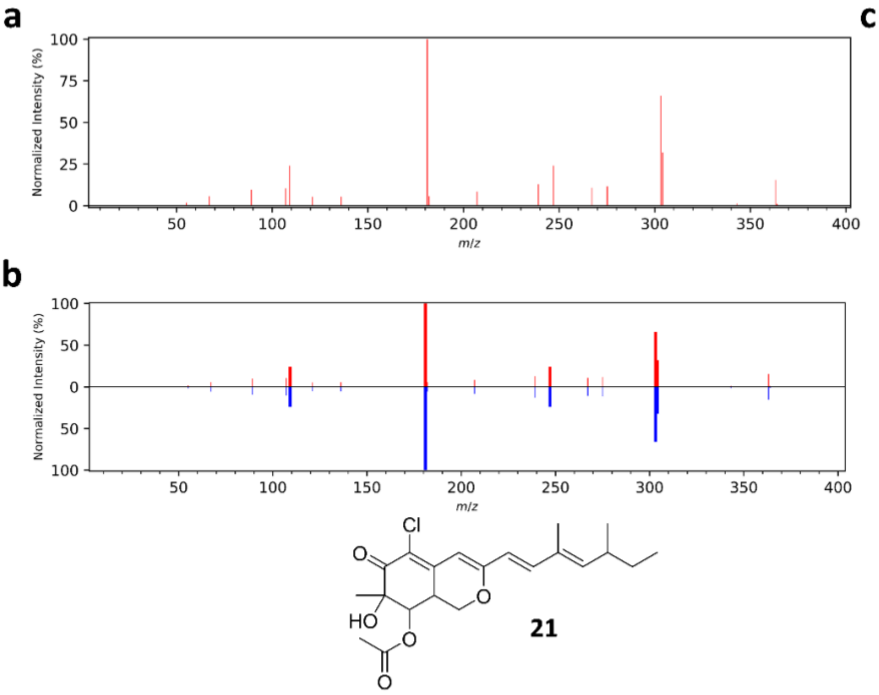

c

Fragment	Score
55.055	0.005
67.0549	0.0156
89.0604	0.0268
107.0848	0.029
**109.1012**	**0.0669**
121.1012	0.0145
136.1202	0.0151
**181.0056**	**0.2789**
182.0134	0.0156
207.0204	0.0234
239.143	0.0357
**247.0529**	**0.0669**
267.1388	0.0301
275.1201	0.0324
**303.1165**	**0.1841**
**304.1213**	**0.0892**
343.1141	0.0038
363.1351	0.0429
363.7875	0.0032
364.1375	0.0018

Figure S25: MS/MS information of compound **22** (*m/z* 405.1467, −0.9 ppm, level 2) from molecular network. (**a**) MS/MS spectrum of compound **22**, (**b**) the mirror plot of MS/MS spectra from compound **22** against itself with the main contributors to cosine score (bold), and (**c**) the fragment list and their contribution to cosine score.

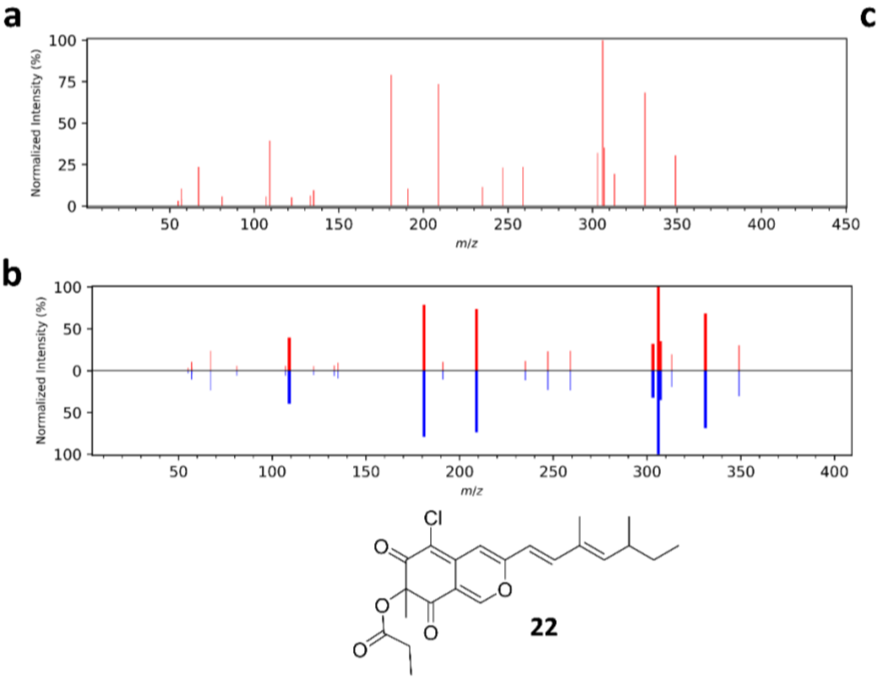

c

Fragment	Score
55.0542	0.0051
57.0338	0.0169
57.0696	0.0067
67.0541	0.038
81.0708	0.0093
107.0859	0.0093
**109.1015**	**0.0633**
122.1091	0.0084
133.1015	0.0101
135.1174	0.0152
**181.0057**	**0.1266**
191.0341	0.0169
**209.0007**	**0.1182**
235.0162	0.0186
247.0528	0.0371
259.053	0.038
**303.1154**	**0.0515**
**306.1025**	**0.1604**
**307.1097**	**0.0566**
313.1447	0.0312
**331.1107**	**0.1097**
349.1216	0.049

Annotation explanation: Its homology of 0.90 with compound **1**, and its *m/z* difference of 14.0158 corresponding to the addition of a CH_2.

Figure S26: MS/MS information of compound **23** (*m/z* 415.1312, −1.3 ppm, level 0) from molecular network. (**a**) MS/MS spectrum of compound **23**, (**b**) the mirror plot of MS/MS spectra from compound **23** against itself with the main contributors to cosine score (bold), and (**c**) the fragment list and their contribution to cosine score.

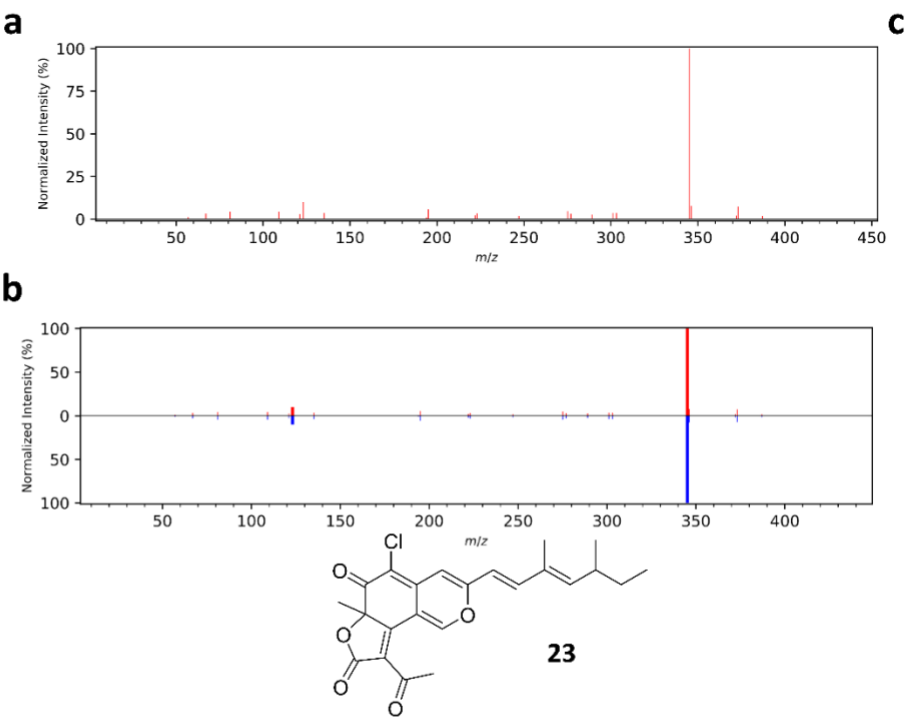

c

Fragment	Score
57.0694	0.0061
67.0535	0.0174
81.0692	0.0241
109.1003	0.0241
121.1005	0.0163
**123.1159**	**0.0555**
135.1158	0.0204
194.0112	0.0048
195.0192	0.0315
222.0063	0.0124
223.0134	0.0185
247.0506	0.0104
275.1177	0.0259
276.9873	0.0168
289.061	0.0146
301.0598	0.0204
303.112	0.0204
**345.1225**	**0.5552**
346.1256	0.0426
372.1088	0.0107
373.1172	0.0407
387.1321	0.0104

Annotation explanation: Its *m/z* difference of 2.0156 with compound **4,** that correspond to a difference of H_2 (loss of an unsaturation).

Figure S27: MS/MS information of compound **24** (*m/z* 357.1705, −2.4 ppm, level 2) from molecular network. (**a**) MS/MS spectrum of compound **24**, (**b**) the mirror plot of MS/MS spectra from compound **24** with compound **1** with neutral losses from acetylation (bold), and (**c**) the common fragments and neutral losses with their contribution to cosine score.

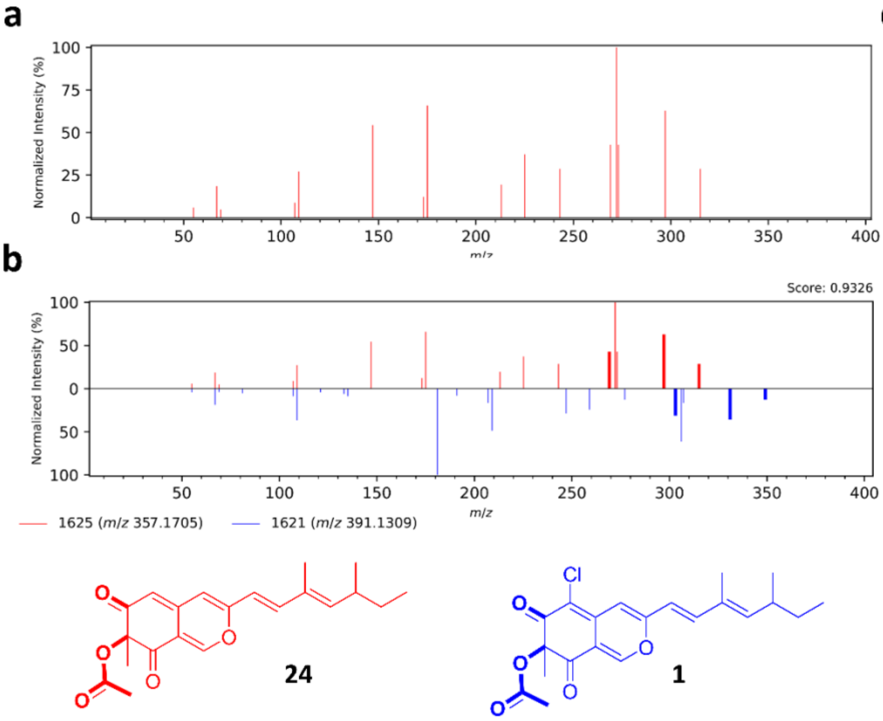

c

Fragment	Score
55.0542	0.0087
67.0544	0.0351
69.0701	0.0082
107.0858	0.0165
109.1014	0.0595

Neutral loss	Score
**42.0107**	**0.0363**
**60.0209**	**0.0896**
84.0213	0.0502
85.0289	0.1483
**88.016**	**0.0694**
114.0678	0.0363
132.0787	0.0568
144.0789	0.0446
182.1311	0.1069
184.1104	0.0269
210.1261	0.1394

Figure S28: MS/MS information of compound **25** (*m/z* 371.1862, −2.4 ppm, level 2) from molecular network. (**a**) MS/MS spectrum of compound **25**, (**b**) the mirror plot of MS/MS spectra from compound **25** with compound **22** with neutral losses from propionylation (bold), and (**c**) the common fragments and neutral losses with their contribution to cosine score.

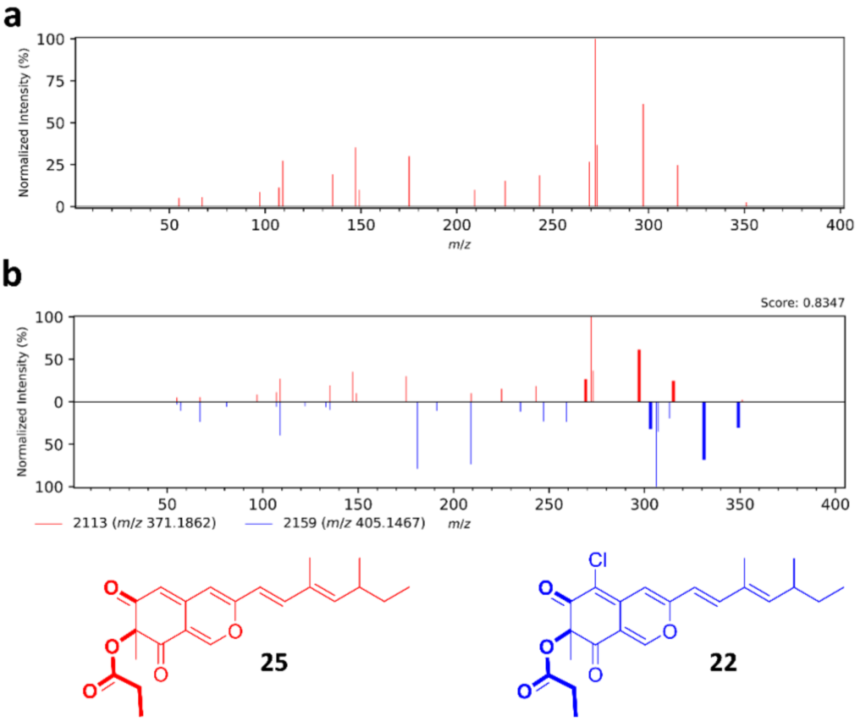

c

Fragment	Score
67.0545	0.022
107.085	0.0153
109.1009	0.062
135.1172	0.0255

Neutral Loss	Score
**56.0271**	**0.0518**
**74.0372**	**0.1222**
98.0376	0.0678
99.0446	0.1887
**102.0297**	**0.0552**
146.0962	0.036
196.1473	0.0887
224.1421	0.0996

Figure S29: MS/MS information of compound **26** (*m/z* 419.1619, 0.2 ppm, t_R = 16.44 min, level 3) from molecular network. (a) MS/MS spectrum of compound **26**, (b) the mirror plot of MS/MS spectra from compound **26** with compound **1** with fragment from acylation loss (bold), and (**c**) the common fragments with their contribution to cosine score.

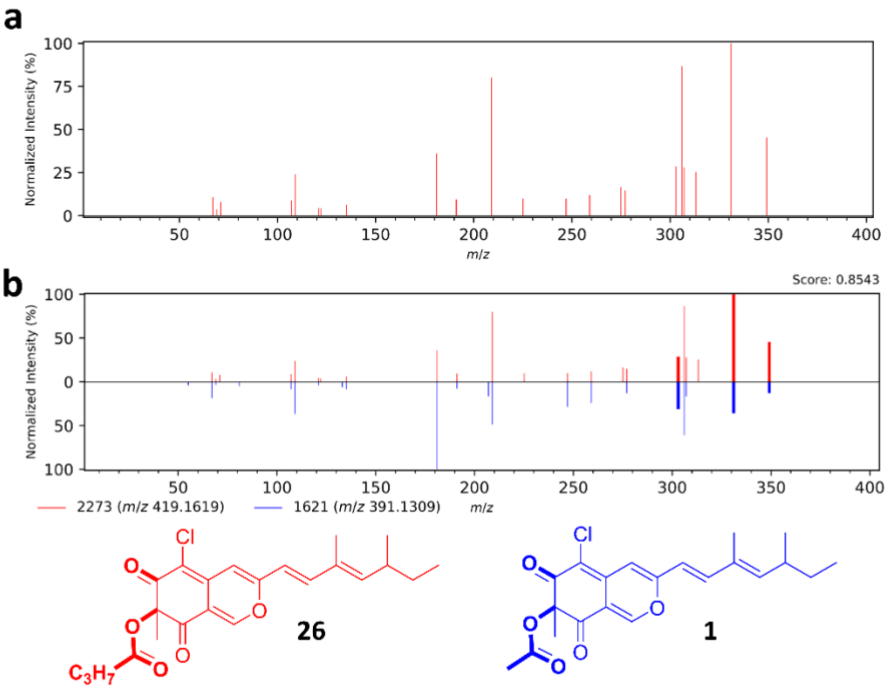

c

Fragment	Score
67.0541	0.0265
69.0697	0.0071
107.0854	0.0162
109.1014	0.0556
121.1013	0.0079
135.1165	0.0138
181.0053	0.1128
191.0325	0.0161
209.0008	0.1172
247.0521	0.0318
259.0526	0.0321
277.063	0.0258
**303.1145**	**0.0564**
306.1021	0.1371
307.1091	0.0403
**331.11**	**0.1123**
**349.1206**	**0.0454**

Annotation explanation: Its MS/MS comparison with compound **1** exhibits common fragments of *m/z* 349.1206, 331.1100, 303.1145, its elution 0.72 min after compound **22**, and 1.49 min after compound **1**.

Figure S30: MS/MS information of compound **27** (*m/z* 385.2018, −2.2 ppm, t_R = 15.32 min, level 3) from molecular network. (**a**) MS/MS spectrum of compound **27**, (**b**) the mirror plot of MS/MS spectra from compound **27** with compound **26** with neutral losses from butanoylation loss (bold) and (**c**) the common fragments and neutral losses with their contribution to cosine score.

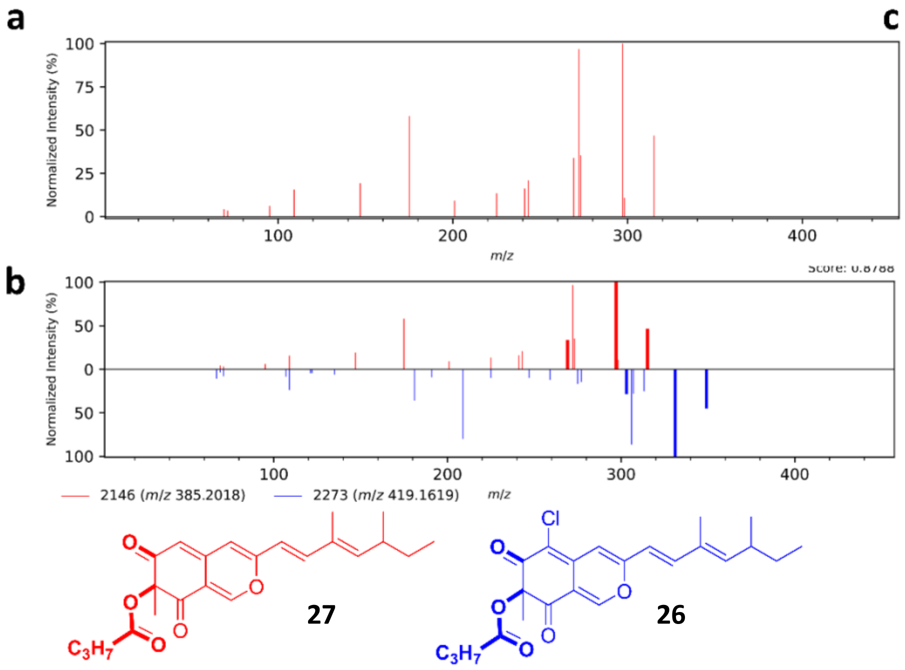

c

Fragment	Score
69.0694	0.0075
71.049	0.0097
109.1002	0.0362
**Neutral Loss**	**Score**
**70.0424**	**0.0861**
**88.0524**	**0.187**
112.0537	0.0589
113.0611	0.1712
**116.0477**	**0.0583**
142.1001	0.0328
144.1158	0.0307
160.1109	0.0237
210.163	0.1274
238.1585	0.0493

Annotation explanation: Its MS/MS comparison with compound **26** exhibits neutral losses of 70.0424, 88.0524, 116.0477 Da, its elution 0.84 min after **26**, and 1.65 min after compound **25** supported its annotation.

Figure S31: MS/MS information of compound **28** (*m/z* 433.1777, −0.2 ppm, t_R = 17.15 min, level 3) from molecular network. (**a**) MS/MS spectrum of compound **28**, (**b**) the mirror plot of MS/MS spectra from compound **28** against compound **1** with fragment from acylation loss (bold) and (**c**) the common fragments and neutral loss with their contribution to cosine score.

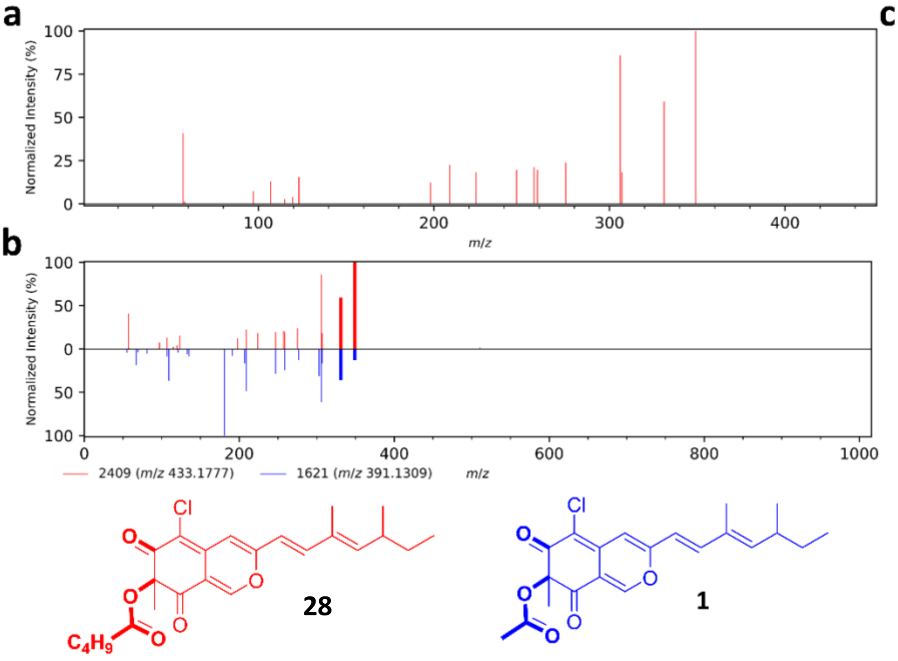

c

Fragment	Score
107.0846	0.0213
209.0029	0.0668
247.0519	0.0479
259.0493	0.0442
306.1005	0.1466
307.1086	0.035
**331.1096**	**0.0927**
**349.1195**	**0.0724**

Neutral Loss	Score
310.0614	0.0184

Raw MS/MS spectra from compound **28** from *m/z* 255 to 375.

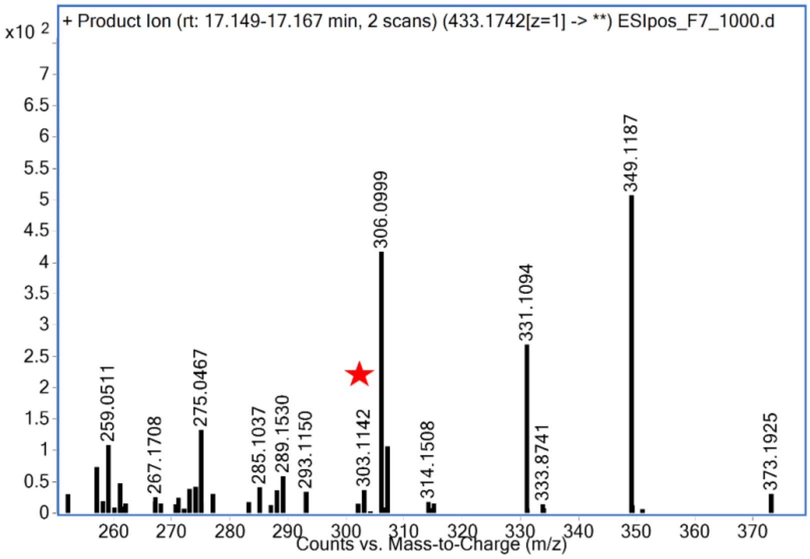

Annotation explannation: Its MS/MS comparison with **1** exhibits common fragments of *m/z* 349.1206 and 331.1100. Fragment C (*m/z* 303.1100, red star) was not found within MS/MS spectra in the molecular network but was found within raw data. Its exclusion is explained by the processing parameters to construct a molecular network, that excludes less intense pics in certain *m/z* windows. Compound 28 elutes 0.69 min after compound **28**, 1.43 min after compound **22**, and 2.20 min after compound **1**.

Figure S32: MS/MS information of compound **29** (*m/z* 399.2169, −0.8 ppm, t_R = 16.09 min, level 3) from molecular network. (**a**) MS/MS spectrum of compound **29**, (**b**) the mirror plot of MS/MS spectra from compound **29** against compound **24** with fragments from acylation loss (bold) and (**c**) the common fragments with their contribution to cosine score.

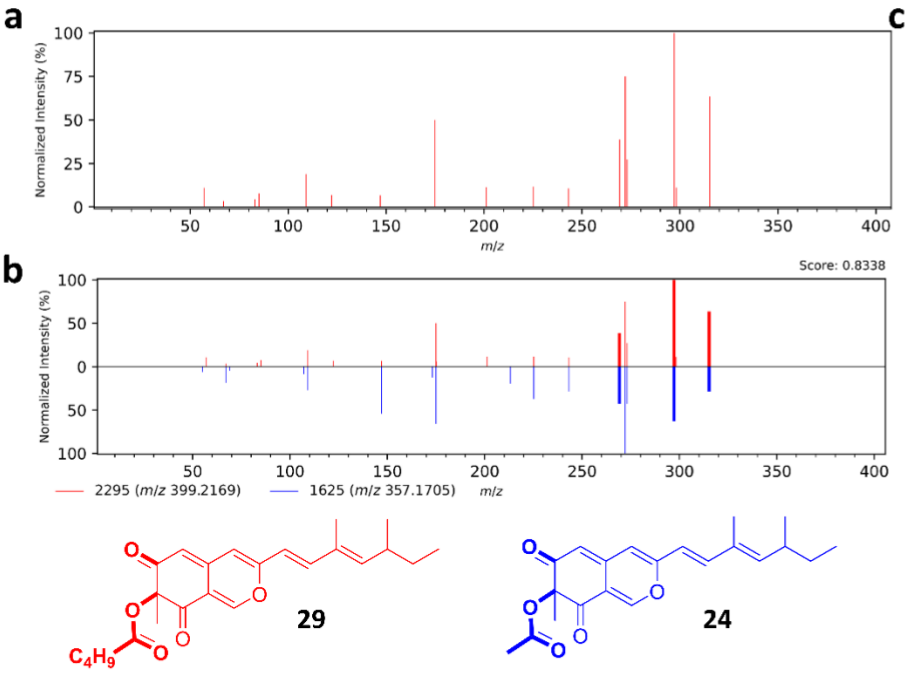

c

Fragment	Score
67.0542	0.0155
109.1013	0.044
147.0451	0.0368
175.0388	0.1115
225.0913	0.0404
243.101	0.034
**269.1522**	**0.0792**
272.1409	0.1685
273.1476	0.0665
**297.1491**	**0.1543**
**315.1588**	**0.083**

Annotation explanation: Its MS/MS comparison with compound **24** exhibiting common fragments of *m/z* 315.588, 297.1491, 269.1522, and its elution 0.77 min after compound **27**, 1.59 min after compound **25** and 2.41 min after compound **24** explained its annotation.

Figure S33: MS/MS information of compound **30** (m/z 349.1205, −1.1 ppm, t_R = 13.09 min, level 2) from molecular network. (**a**) MS/MS spectrum of compound **30**, (**b**) the mirror plot of MS/MS spectra from compound **30** against compound **1**, (**c**) the common fragments and neutral losses with their contribution to cosine score.

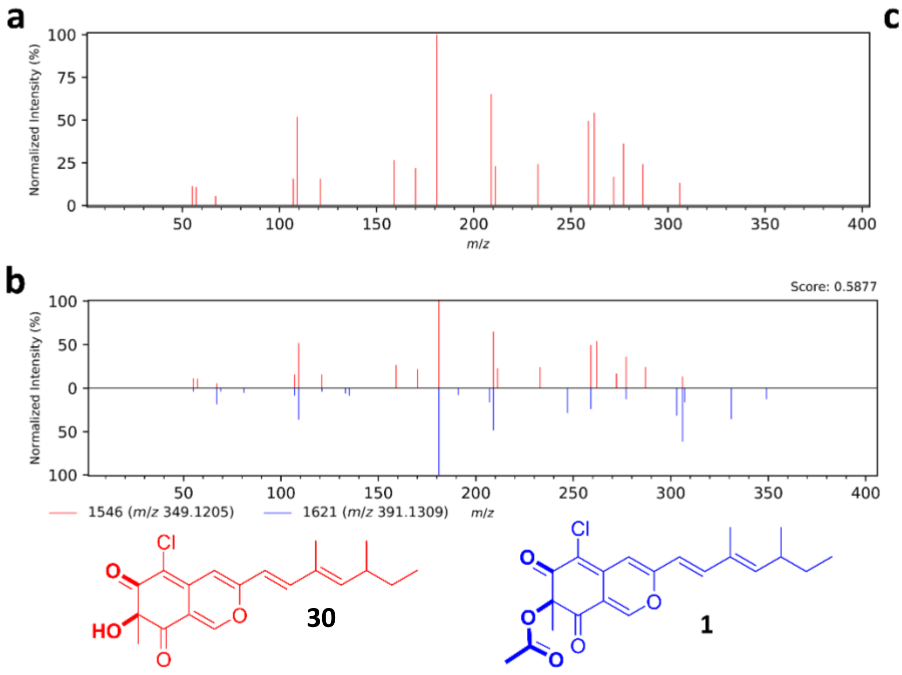

c

Fragment	Score
55.0553	0.0128
67.0534	0.0191
107.0847	0.0218
109.1011	0.0816
181.0055	0.1878
208.9989	0.1056
259.0495	0.065
277.0641	0.0405
306.1031	0.0536

MS/MS spectrum of compound **30** with a zoom on m/z from 295 to 355.

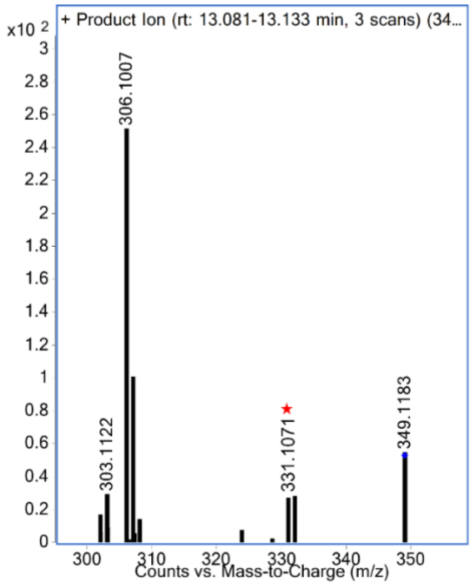

Annotation explanation: the exact mass of compound **30** corresponded to fragment A, compound **30** clustered with compound **1** on molecular network. No fragment B and C were to be found within processed data for molecular network but inspection of raw data confirmed the presence of fragment B (m/z 331.1071) and C (m/z 303.1122) (red star)

Figure S34: MS/MS information of compound **31** (*m/z* 315.1594, −1.0 ppm, t_R = 11.97 min, level 3) from molecular network. (**a**) MS/MS spectrum of compound **31**, (**b**) the mirror plot of MS/MS spectra from compound **31** against compound **24** (**c**) the common fragments with their contribution to cosine score.

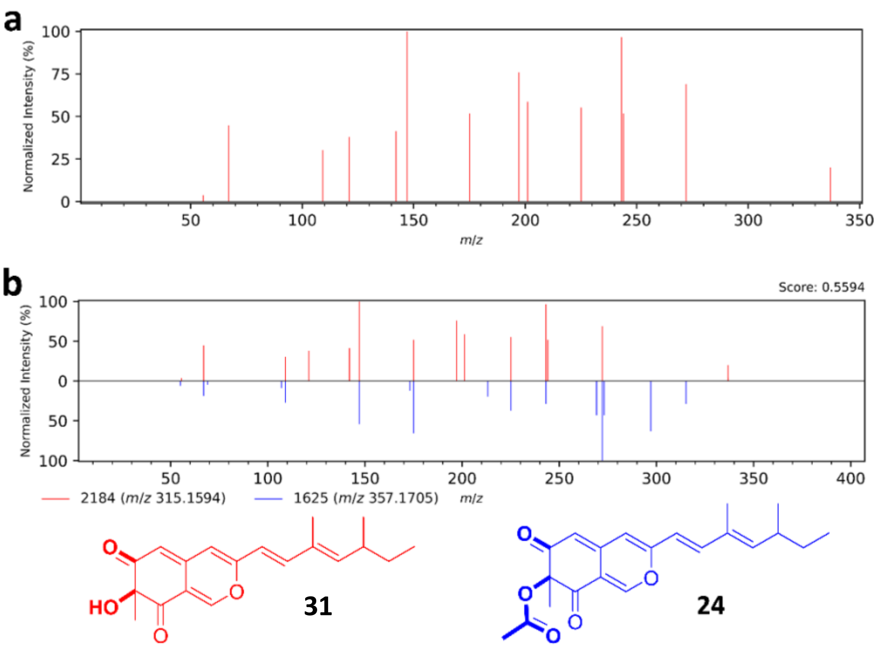

c

Fragment	Score
67.0536	0.0436
109.1026	0.0433
147.0457	0.1113
175.0409	0.088
225.0909	0.0684
243.1007	0.0793
272.1422	0.1254

Annotation explanation: Despite being excluded from the t-SNE, its cosine scores against compounds **30** and **24**, its two closer annotated structural analogs were 0.53 and 0.56. Moreover, fragment D (*m/z* 147.0457) is found within MS/MS data of compound **31**.

Figure S35: MS/MS information of compound **32** (*m/z* 359.1859, −1.7 ppm, level 3) from molecular network. (**a**) MS/MS spectrum of compound **32**, (**b**) the mirror plot of MS/MS spectra from compound **31** against compound **24** (**c**) the common fragments and neutral losses with their contribution to cosine score.

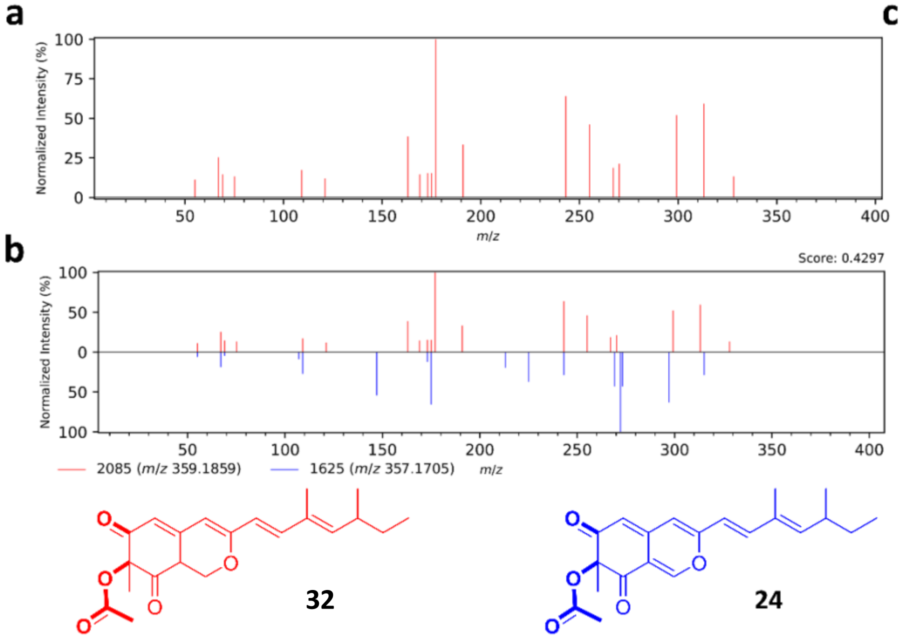

Fragment	Score
55.0545	0.0129
67.0542	0.0367
69.0699	0.0143
109.1008	0.0367
243.1019	0.0723

Neutral Loss	Score
182.1309	0.1371
60.0219	0.0967
184.1117	0.0232

Annotation explanation: compound **32** calculated molecular formula was $C_{21}H_{26}O_5$. Annotation as an analog of compound **24** with one less insaturation was favoured, as this scaffold is reported for some azaphilones like compound **17.**

Figure S36: MS/MS information of compound **33** (*m/z* 419.1864, −2.6 ppm, level 2) from molecular network. (**a**) MS/MS spectrum of compound **33**, (**b**) the mirror plot of MS/MS spectra from compound **33** against compound **36** with fragment from acylation loss (bold) (**c**) the common fragments and neutral losses with their contribution to cosine score.

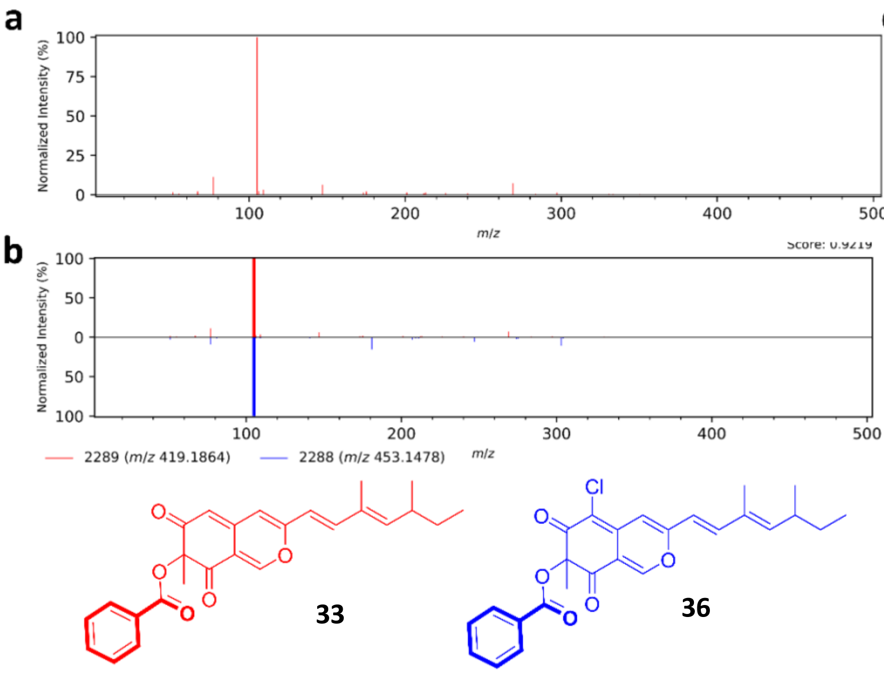

c

Fragment	Score
51.0225	0.0138
77.0394	0.0656
**105.0338**	**0.6408**
106.0377	0.0117
109.101	0.0129

Neutral Loss	Score
122.0369	0.0039
150.032	0.0567
179.0681	0.0091
206.0973	0.0182
244.1468	0.0123
246.1263	0.0135
272.1422	0.0634

Figure S37: MS/MS information of compound **34** (*m/z* 468.1575, −0.6 ppm, level 2) from molecular network. (**a**) MS/MS spectrum of compound **34**, (**b**) the mirror plot of MS/MS spectra from compound **34** against compound **36** with fragment from azaphilone scaffold loss (bold) (**c**) the common fragments and neutral losses with their contribution to cosine score.

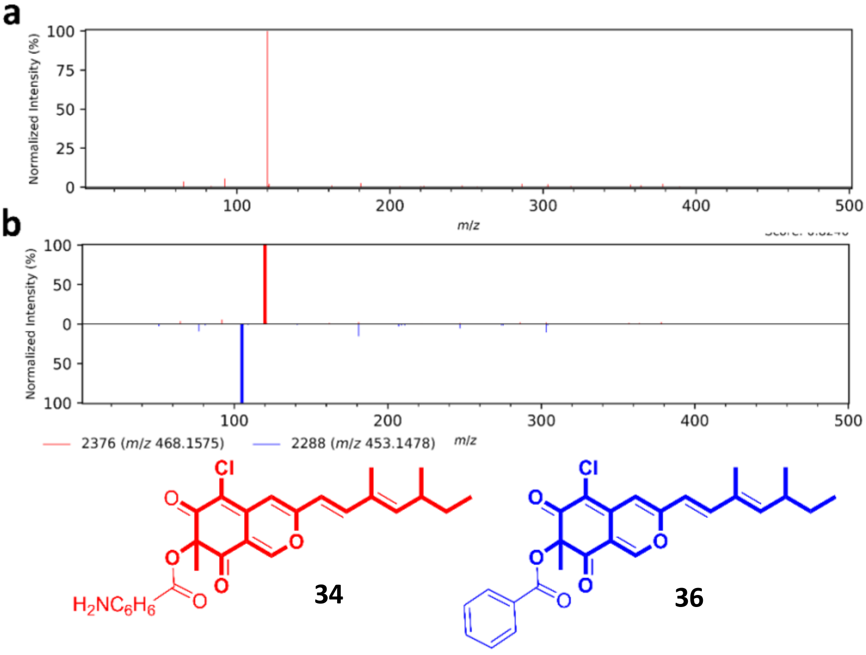

c

Fragment	Score
181.0079	0.0419
247.0521	0.0164
303.1164	0.0305

Neutral Loss	Score
347.1114	0.0115
**348.1132**	**0.6757**
376.1072	0.0479

Annotation explanation: In a similar fashion to compound **36** its most intense fragment correspond to the aminobenzoyl.

Figure S38: MS/MS information of compound **35** (*m/z* 407.1265, −2.2 ppm, level 3) from molecular network. (**a**) MS/MS spectrum of compound **35** (**b**) the mirror plot of MS/MS spectra from compound **35** against compound **1** with fragments from acylation loss (bold) (**c**) the common fragments with their contribution to cosine score.

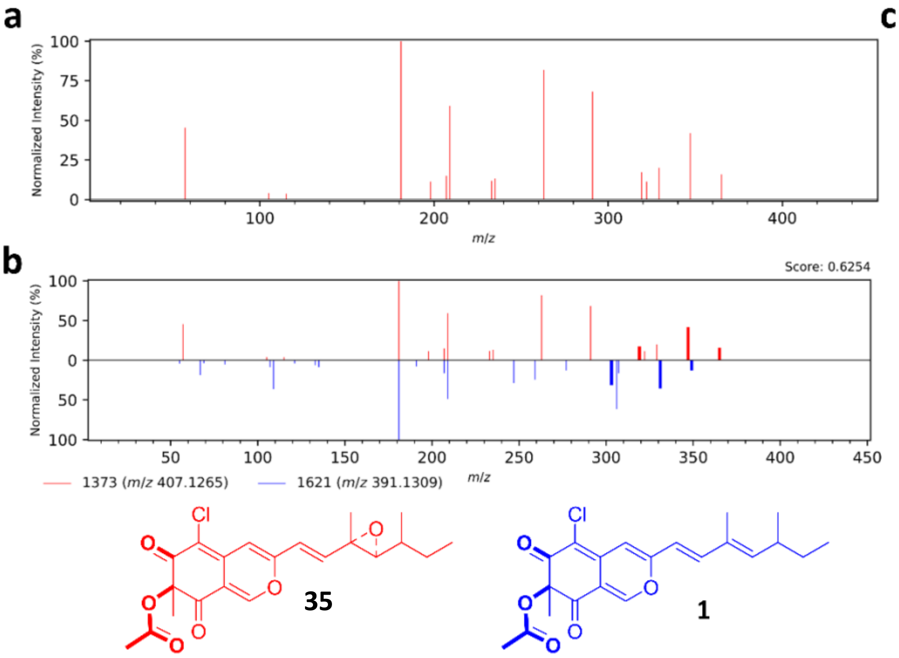

c

Fragment	Score
181.0054	0.1953
207.0209	0.0307
208.9999	0.1046

Neutral Loss	Fragment
**42.0134**	**0.0279**
**60.0222**	**0.0755**
85.0318	0.0516
**88.0186**	**0.0455**
144.0794	0.0944

Annotation explanation: The neutral losses A, B C from the acetylation of compound **35**. An intense common fragment D (*m/z* 181.0054) with other compound **1**’s analogs was found within compound **35** MS/MS spectra explaining the positionning of the epoxydation.

Figure S39: MS/MS information of compound **36** (*m/z* 453.1458, 1.2 ppm, level 2) from molecular network. (**a**) MS/MS spectrum of compound **36**, (**b**) the mirror plot of MS/MS spectra from compound **36** against compound **1** with fragment from acylation loss (bold) (**c**) the common fragments with their contribution to cosine score.

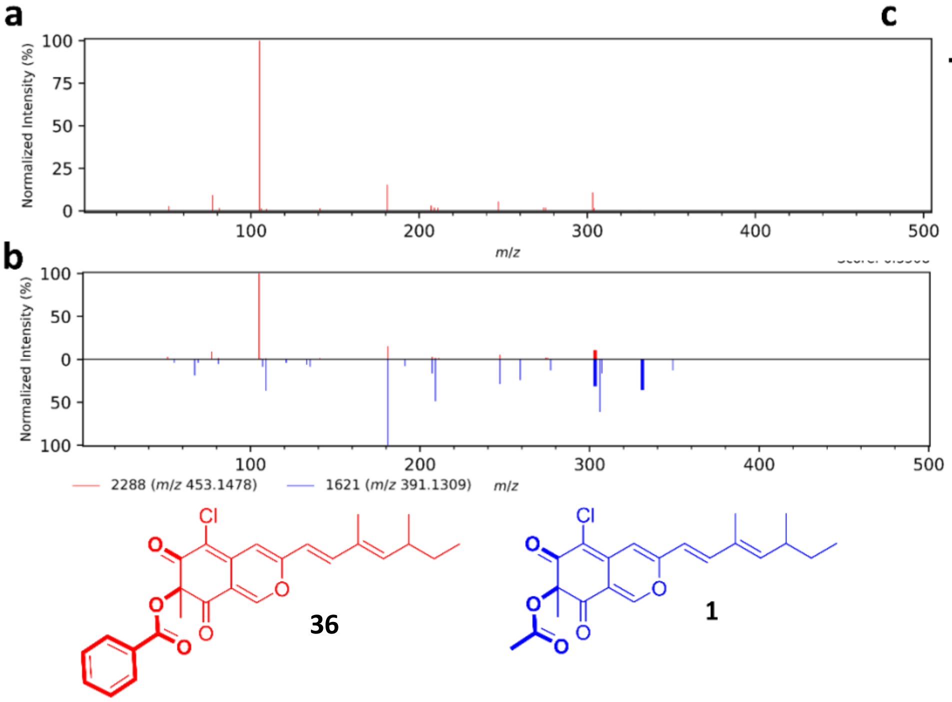

c

Fragment	Score
81.07	0.0106
109.1002	0.0236
181.0061	0.1383
207.0212	0.0254
208.9988	0.0327
247.0528	0.0443
**303.1154**	**0.0648**
**331.1148**	**0.0111**

Annotation explanation: The neutral loss of the acylation indicates a benzaldehyde loss, and its expected 105.0336 Da was found within MS/MS spectra.

Figure S40: MS/MS information of compound **37** (*m/z* 363.1360, −0.7 ppm, t_R = 14.58 min, level 2) from molecular network. (a) MS/MS spectrum of compound **37**, (**b**) the mirror plot of MS/MS spectra from compound **37** against compound **22** with fragment from acylation loss (bold) (**c**) the common fragments with their contribution to cosine score.

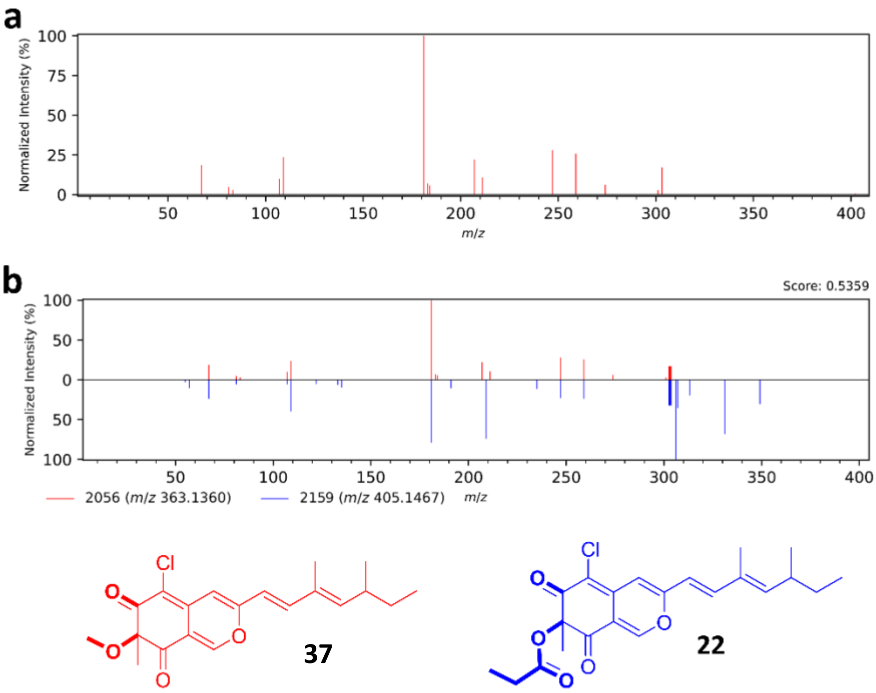

c

Fragment	Score
67.0539	0.0496
81.0693	0.0124
107.0851	0.018
109.1008	0.0721
181.0051	0.21
247.0522	0.06
259.0518	0.0583
**303.1145**	**0.0554**

Annotation explanation: the presence of specific fragment C, compound **37** exact mass and its presence in cluster A.

Figure S41: MS/MS information of compound **38** (*m/z* 435.1575, −1.4 ppm, level 3) from molecular network. (a) MS/MS spectrum **38**, (**b**) the mirror plot of MS/MS spectra from compound **38** against compound **26** with fragments from acylation loss (bold) (**c**) the common fragments with their contribution to cosine score.

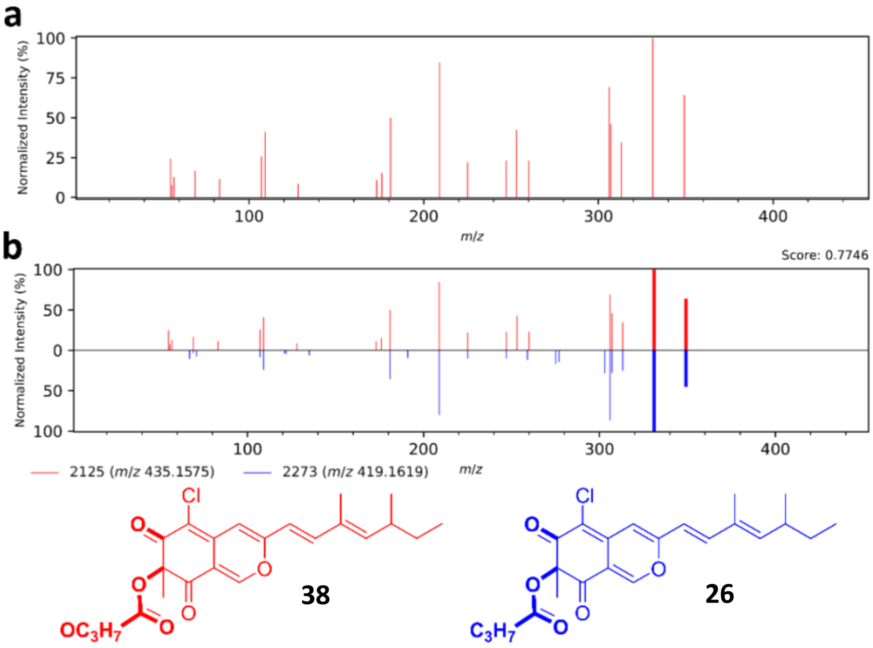

c

Fragment	Score
69.0702	0.012
107.0863	0.0228
109.1019	0.0481
181.0047	0.065
209.0002	0.126
225.1272	0.0226
247.0515	0.0233
306.1031	0.1186
307.1084	0.0551
313.1437	0.0454
**331.1109**	**0.1532**
**349.1205**	**0.0826**

Figure S42: MS/MS information of compound **39** (*m/z* 452.1631, −1.8 ppm, level 2) from molecular network. (**a**) MS/MS spectrum of compound **39**, (**b**) the mirror plot of MS/MS spectra from compound **39** against compound **36** with neutral losses from benzaldehyde loss (bold) (**c**) the common fragments and neutral losses with their contribution to cosine score.

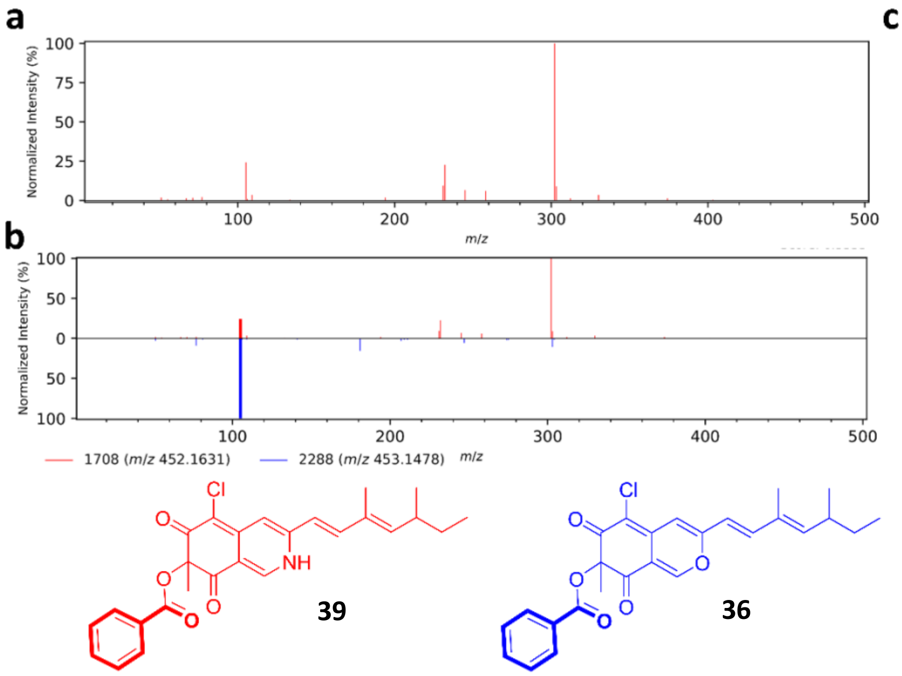

c

Fragment	Score
51.0239	0.0125
77.0396	0.0244
**105.0333**	**0.2711**
106.0392	0.0066
109.1009	0.0114

Neutral Loss	Score
122.0361	0.0054
149.0284	0.0214
150.0326	0.1807

Figure S43: MS/MS information of compound **40** (*m/z* 434.1734, −1.2 ppm, level 3) from molecular network. (**a**) MS/MS spectrum of compound **40**, (**b**) the mirror plot of MS/MS spectra from compound **40** against compound **38** with neutral losses from acylation loss (bold) (**c**) the common fragments and neutral losses with their contribution to cosine score.

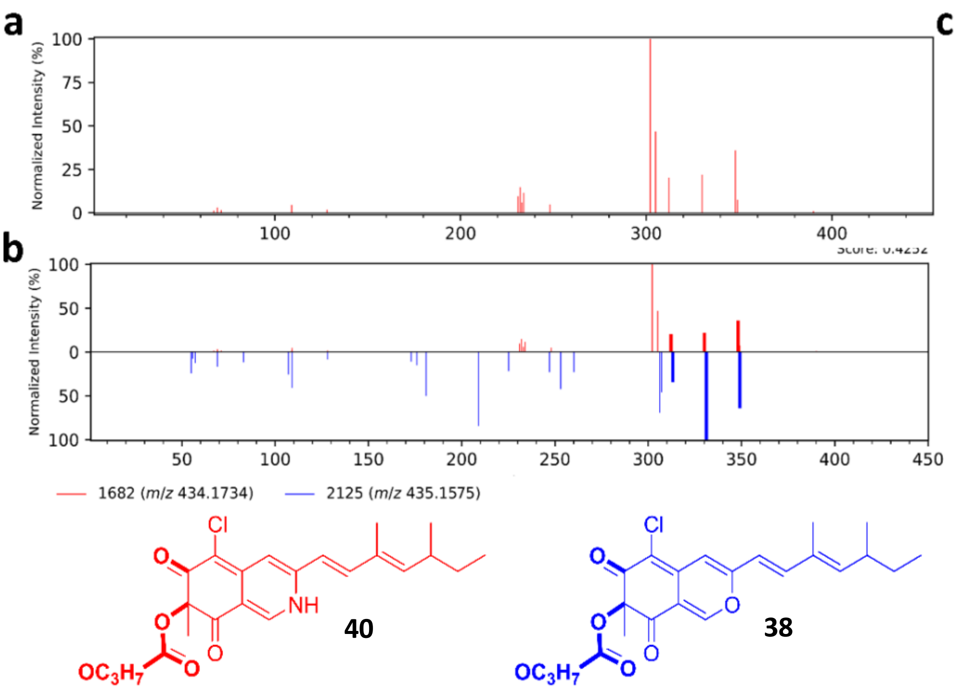

c

Fragment	Score
69.0702	0.0154
109.0997	0.0291

Neutral Loss	Score
**86.0354**	**0.1025**
**104.0474**	**0.0999**
**122.0136**	**0.0566**
129.0549	0.1217

Figure S44: MS/MS information of compound **41** (*m/z* 356.1862, −1.6 ppm, level 2) from molecular network. (a) MS/MS spectrum of compound **41**, (b) the mirror plot of MS/MS spectra from compound **41** with compound **2** with neutral losses from acetylation (bold), and (**c**) the common fragments and neutral losses with their contribution to cosine score.

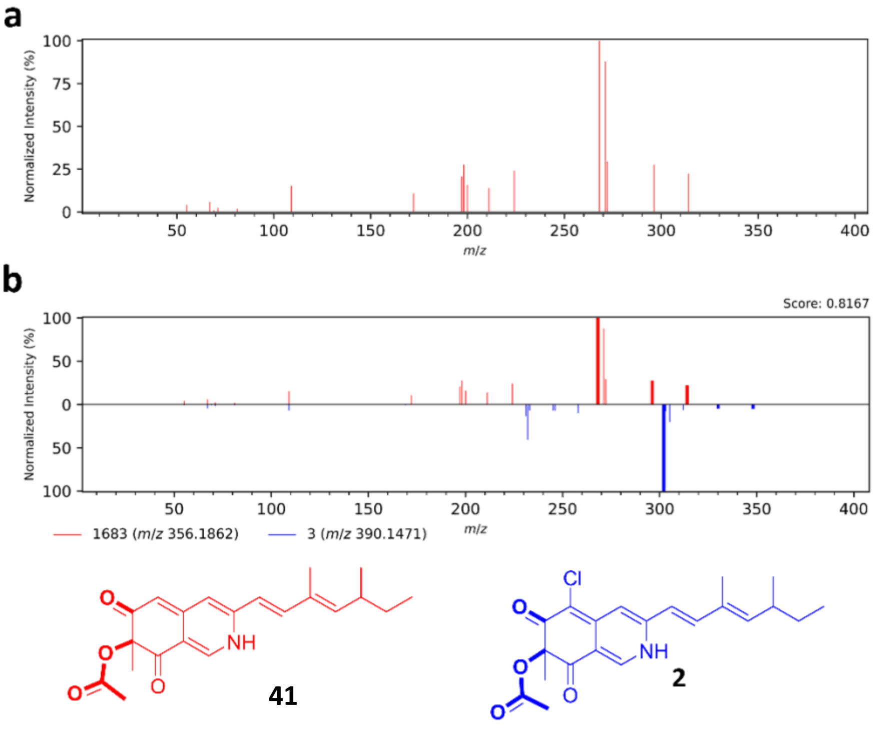

c

Fragment	Score
55.0535	0.0055
67.0536	0.0159
69.0689	0.0026
71.085	0.0063
81.0682	0.0044
109.1014	0.0324

Neutral Loss	Score
**42.0115**	**0.0333**
**60.0228**	**0.0363**
85.0291	0.132
**88.0163**	**0.312**
132.0783	0.0481
145.0858	0.0311
158.0946	0.1045
159.103	0.0523

Figure S45: MS/MS information of compound **42** (*m/z* 404.1626, −0.7 ppm, level 2) from molecular network. (**a**) MS/MS spectrum of compound **42**, (**b**) the mirror plot of MS/MS spectra from compound **42** with compound **22** with neutral losses from propionylation (bold), and (**c**) the common fragments and neutral losses with their contribution to cosine score.

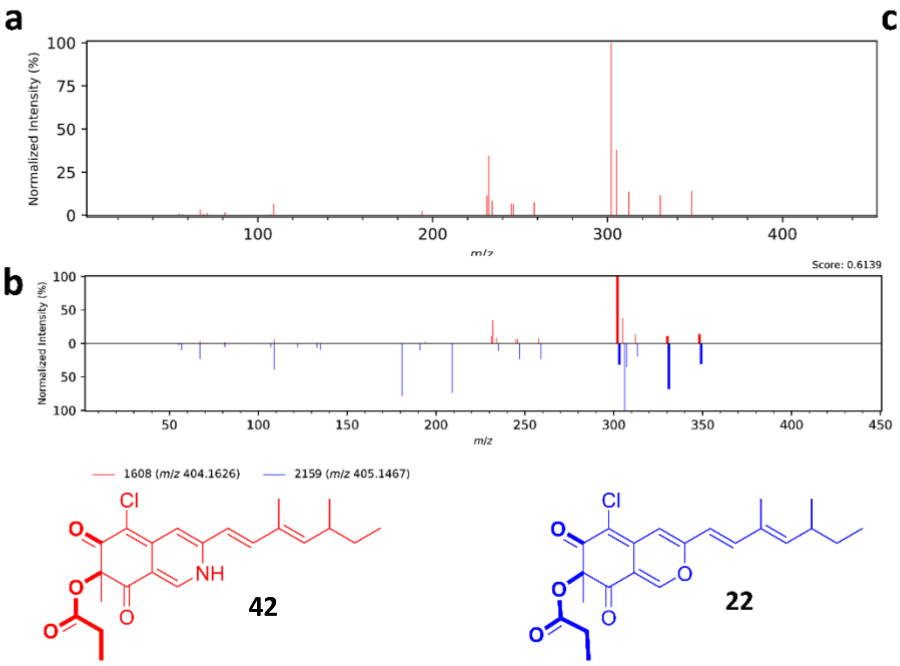

c

Fragment	Score
55.0539	0.0038
57.0333	0.0052
67.054	0.0214
81.0701	0.0074
107.0854	0.0038
109.1015	0.0396

Neutral Loss	Score
**56.0262**	**0.0508**
**74.0373**	**0.0683**
92.0033	0.0396
99.0448	0.1497
102.0317	0.1377
146.0943	0.0326
158.095	0.0299
170.1302	0.024

Figure S46: MS/MS information of compound **43** (*m/z* 362.1524, −1.8 ppm, t_R = 11.12 min, level 2) from molecular network.

(a) MS/MS spectrum of compound **43**, (**b**) the mirror plot of MS/MS spectra from compound **43** against Sclerotioramine **2** with fragment from acylation loss (bold) (**c**) the common fragments with their contribution to cosine score.

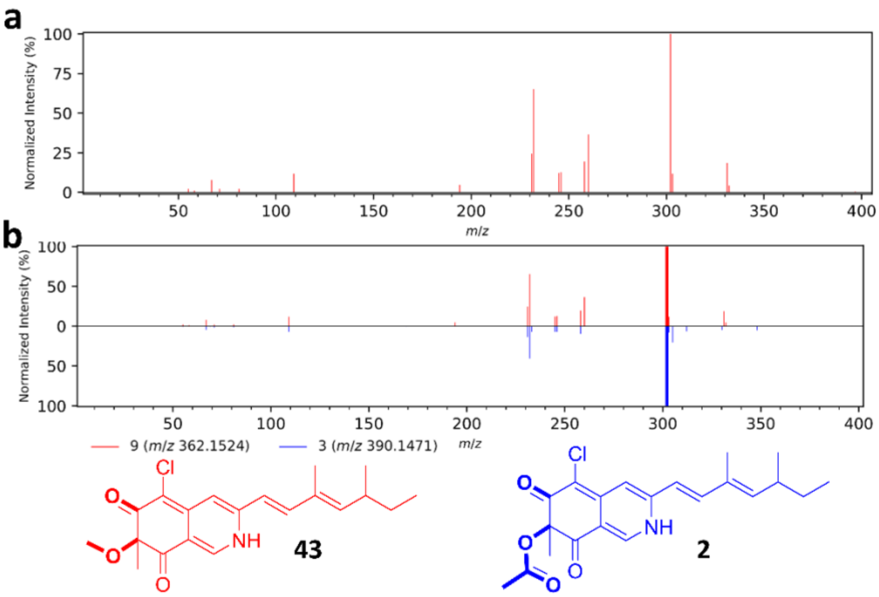

c

Fragment	Score
55.0536	0.0044
67.0541	0.0203
71.0858	0.0066
81.0697	0.0052
109.1016	0.0316
231.0455	0.0628
232.0526	0.178
245.0606	0.0322
246.0681	0.0327
258.0687	0.0479
**302.1315**	**0.3456**
303.1347	0.0331

Annotation explanation: the presence of Fragment C (*m/z* 303.1145), compound **43** exact mass and its presence in cluster B2 explained its annotation.

Figure S47: MS/MS information of compound **44** (*m/z* 432.1945, −2.1 ppm, t_R = 13.65 min, level 3) from molecular network.

(**a**) MS/MS spectrum of compound **44**, (**b**) the mirror plot of MS/MS spectra from compound **44** against compound **29** with neutral losses from acylation loss (bold) and (**c**) the common fragments and neutral losses with their contribution to cosine score.

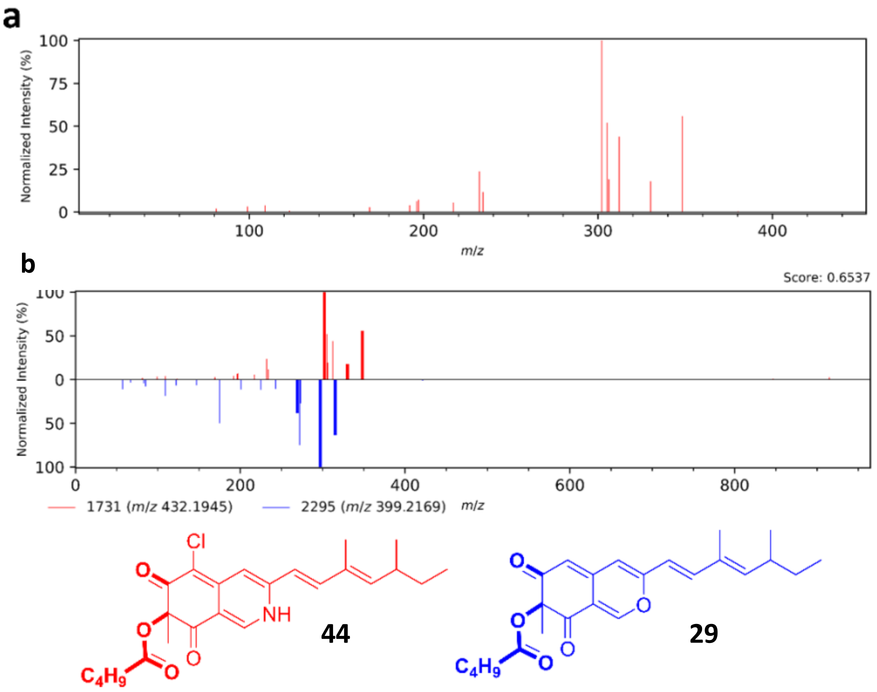

c

Fragment	Score
109.1017	0.0206

Neutral Loss	Score
**84.057**	**0.1447**
**102.0686**	**0.1029**
126.0685	0.0555
127.0749	0.1514
**130.0633**	**0.1507**
198.1623	0.0278

Figure S48: MS/MS information of compound **45** (*m/z* 418.1784, −1.1 ppm, t_R = 12.94 min, level 3) from molecular network. (**a**) MS/MS spectrum **45**, (**b**) the mirror plot of MS/MS spectra from compound **45** with compound **26** with fragment from acylation loss (bold) and (**c**) the common fragments with their contribution to cosine score.

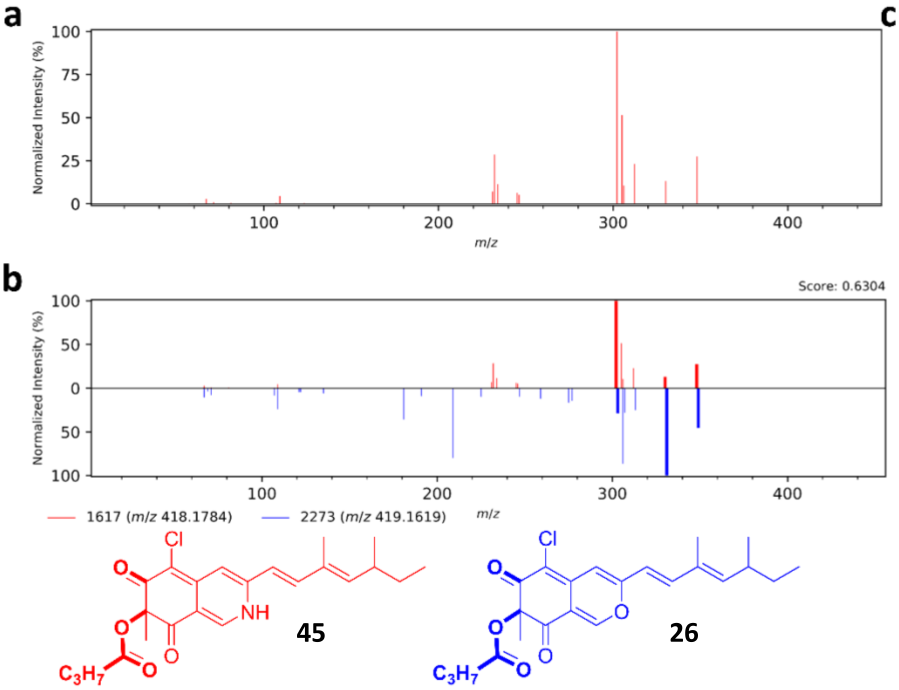

c

Fragment	Score
67.0541	0.0132
71.0484	0.0043
107.0847	0.005
109.1011	0.0254

Neutral Loss	Score
**70.0422**	**0.0854**
**88.0524**	**0.0878**
106.0189	0.0587
112.0539	0.0417
113.0605	0.1618
**116.0477**	**0.1297**
172.111	0.0174

Figure S49: MS/MS information of compound **46** (*m/z* 406.1425, −2.3 ppm, level 3) from molecular network. (**a**) MS/MS spectrum of compound **46**, (**b**) the mirror plot of MS/MS spectra from compound **45** against compound **2** with neutral losses from acylation loss (bold) (**c**) the common fragments and neutral losses with their contribution to cosine score.

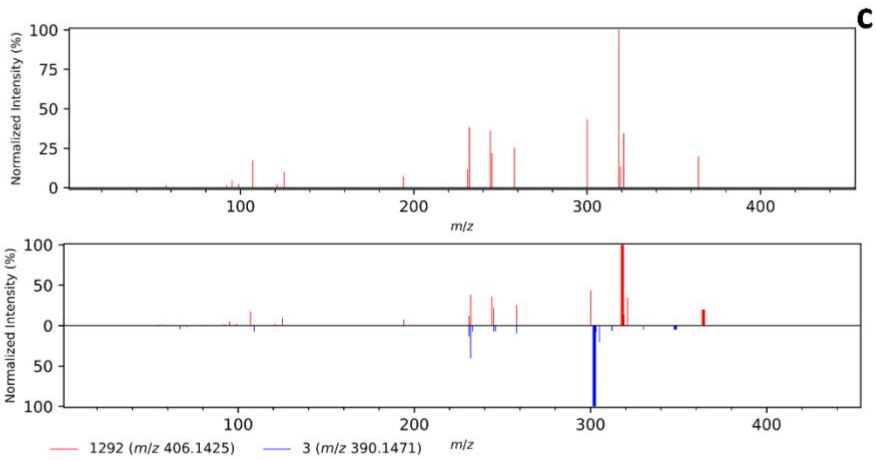

c

Fragment	Score
57.0703	0.0017
231.0439	0.0407
232.0527	0.1266
245.0596	0.04
258.0685	0.0508

Neutral Loss	Score
**42.0118**	**0.0324**
85.0291	0.0851
87.0145	0.0331
**88.0167**	**0.3212**
281.0471	0.0271

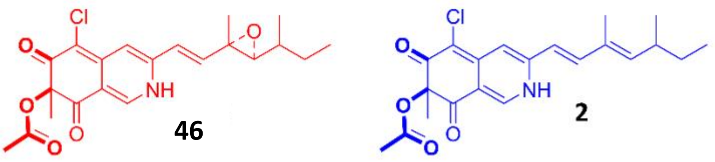

Annotation explanation: the presence of acetylation typical neutral losses within compound **46** fragmentation spectra indicates that the epoxyde is elsewhere on the molecules. Position on the 3,5-dimethyl-1,3-heptadienyl unit was favoured.

Figure S50: MS/MS information of compound **47** (*m/z* 448.1890, −1.1 ppm, level 2) from molecular network. (**a**) MS/MS spectrum of compound **47**, (**b**) the mirror plot of MS/MS spectra from compound **47** against compound **42** with neutral losses from acylation loss (bold) (**c**) the common fragments and neutral losses with their contribution to cosine score.

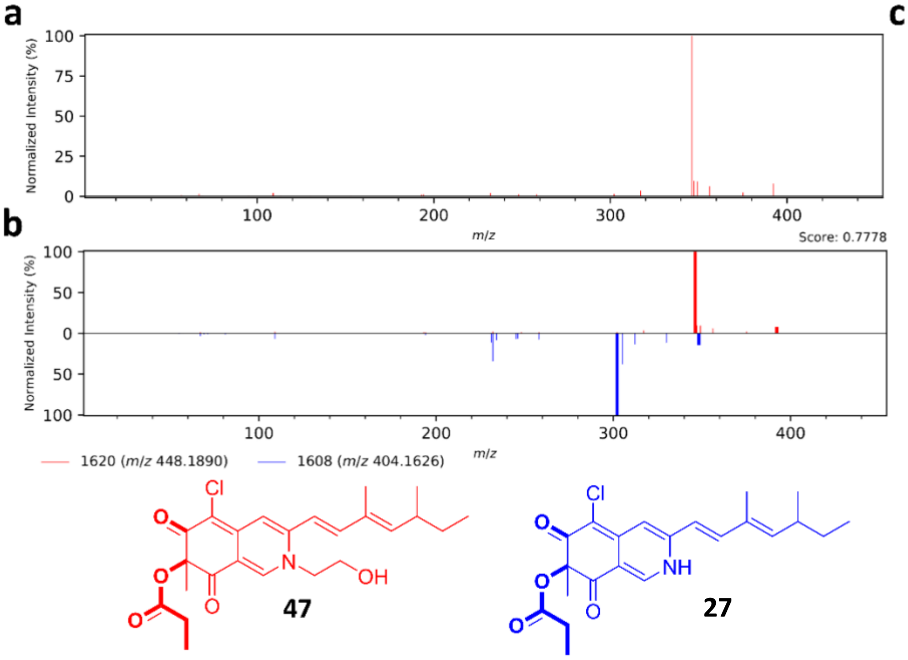

c

Fragment	Score
67.0534	0.0111
69.0681	0.0029
109.0997	0.0178
194.0359	0.0082
232.0504	0.0417
258.0682	0.0152

Neutral Loss	Score
**56.028**	**0.0522**
92.002	0.0454
99.0453	0.0922
**102.0322**	**0.491**

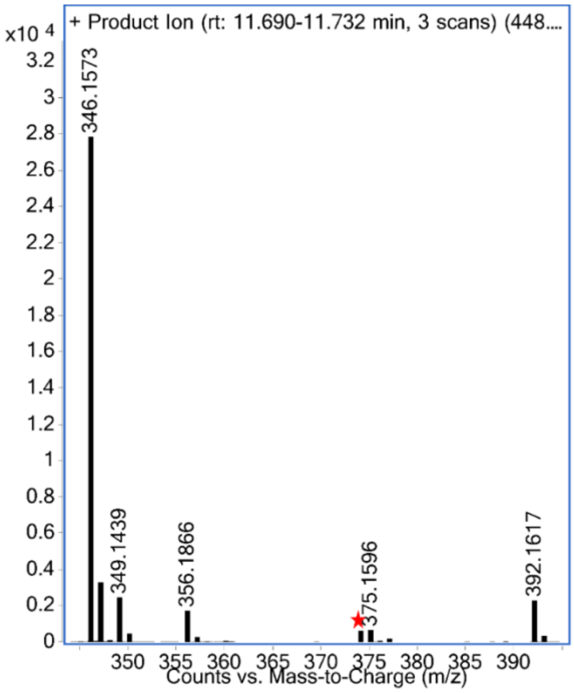

Fragmentation spectrum raw data of compound **47** to detect fragment B (red star) at *m/z* 374.1497

Figure S51: MS/MS information of compound **48** (*m/z* 462.2061, −4.2 ppm, level 2) from molecular network. (**a**) MS/MS spectrum **48**, (**b**) the mirror plot of MS/MS spectra from compound **48** against compound **26** with neutral losses from acylation loss (bold) (**c**) the common fragments and neutral losses with their contribution to cosine score.

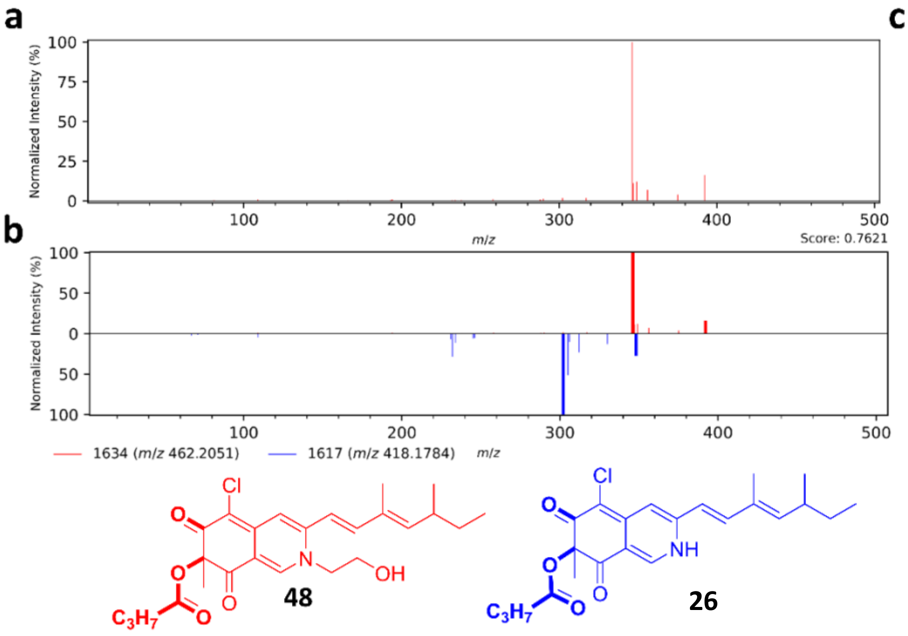

c

Fragment	Score
57.0703	0.0014
81.0687	0.0025
109.1016	0.0103
232.0542	0.0181

Neutral Loss	Score
**70.0421**	**0.0955**
106.0189	0.0571
113.06	0.1133
**116.0472**	**0.4524**
172.11	0.0116

Annotation explanation: The neutral losses from butanoylation were found within its MS/MS spectra, explaining compound **48** annotation.

Figure S52: MS/MS information of compound **49** (*m/z* 478.2001, −2.1 ppm, level 3) from molecular network. (**a**) MS/MS spectrum of compound **49**, (**b**) the mirror plot of MS/MS spectra from compound **49** against compound **5** with fragment from acylation loss (bold) (**c**) the common fragments with their contribution to cosine score.

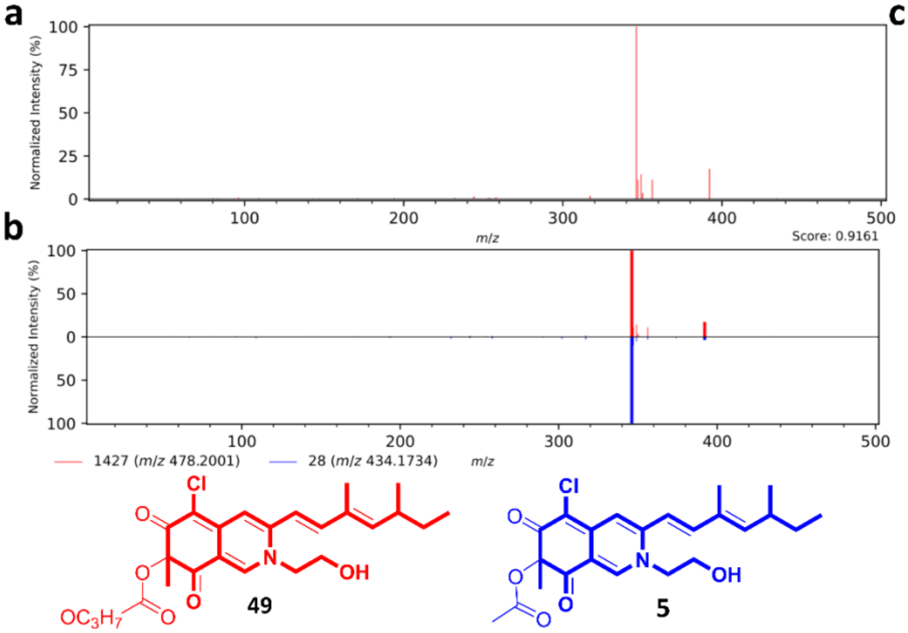

c

Fragment	Score
55.0526	0.0006
57.0688	0.0013
109.1006	0.0061
194.0343	0.005
232.0534	0.0081
244.0513	0.0092
258.0649	0.0097
317.1219	0.0151
**346.1579**	**0.647**
347.1613	0.0679
349.1449	0.0554
356.1858	0.04
**392.1624**	**0.0506**

Annotation explanation: The Fragment A and C *m/z* are the same as compound **5** indicating a difference of acylation length .

Figure S53: MS/MS information of compound **50** (*m/z* 448.1527, −1.3 ppm, level 2) from molecular network. (**a**) MS/MS spectrum of compound **50**, (**b**) the mirror plot of MS/MS spectra from compound **53** against compound **5** with neutral losses from acylation loss (bold) (**c**) the common fragments and neutral losses with their contribution to cosine score.

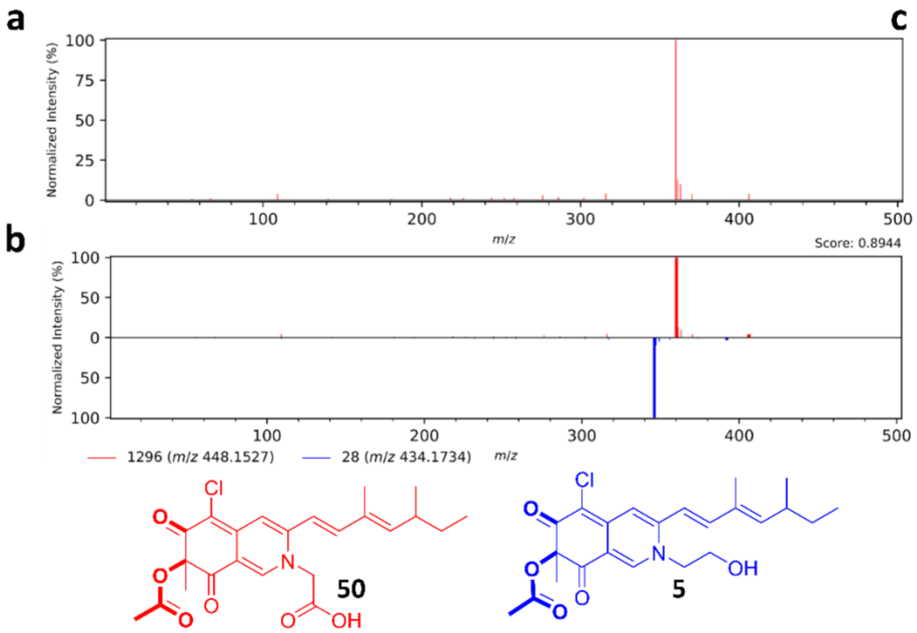

Fragment	Score
55.0558	0.0017
67.0533	0.0068
109.1012	0.016
244.0539	0.0093
258.0677	0.0109

Neutral Loss	Score
**42.0097**	**0.025**
77.9867	0.0251
85.0293	0.0482
87.0134	0.0764
**88.0161**	**0.6749**

Annotation explanation: The the N-chain was 28.0318 (C_2H_4) shorter than compound **6**. Carboxilic acid motive was favoured because several azaphilones with similar function on their carbon chain are aslo described. [2, 3]

Figure S54: MS/MS information of compound **51** (*m/z* 462.1680, −0.5 ppm, level 2) from molecular network. (**a**) MS/MS spectrum of compound **51**, (**b**) the mirror plot of MS/MS spectra from compound **51** against **19** with neutral losses from acylation loss (bold) (**c**) the common fragments and neutral losses with their contribution to cosine score.

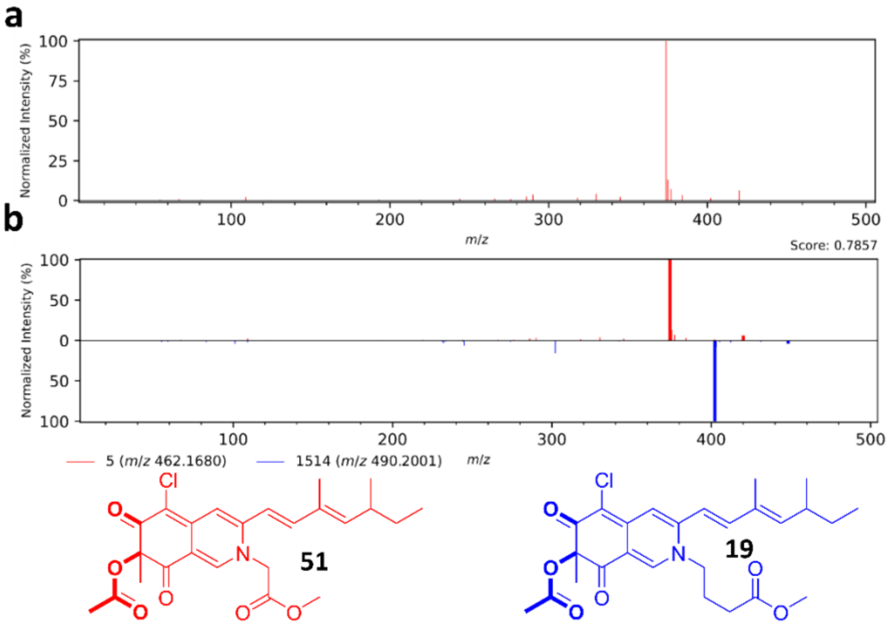

c

Fragment	Score
55.0535	0.0051
109.1009	0.0155
244.0528	0.0079

Neutral Loss	Score
**42.011**	**0.0317**
77.987	0.0183
85.029	0.0263
87.0128	0.0639
**88.0159**	**0.6171**

Annotation explanation: Possesses the same *m/z* as **61** but did not clusterize on the same area of cluster B2. Both molecules can ben discrimated using caracteristic fragment from linear acid chain such as one found for **6** or **20**. **51** was proposed with a methylation at the end of the chain as no caracteristic fragment was found, in a similar fashion as **19**. On the other hand, **61** MS/MS spectrum displays an intense fragment of 73.0287 *m/z*, corresponding to its chain.

Figure S55: MS/MS information of compound **52** (*m/z* 460.1523, −0.4 ppm, level 2) from molecular network. (**a**) MS/MS spectrum of compound **53**, (**b**) the mirror plot of MS/MS spectra from compound **52** against **5** with neutral losses from acylation loss (bold) (**c**) the common fragments and neutral losses with their contribution to cosine score.

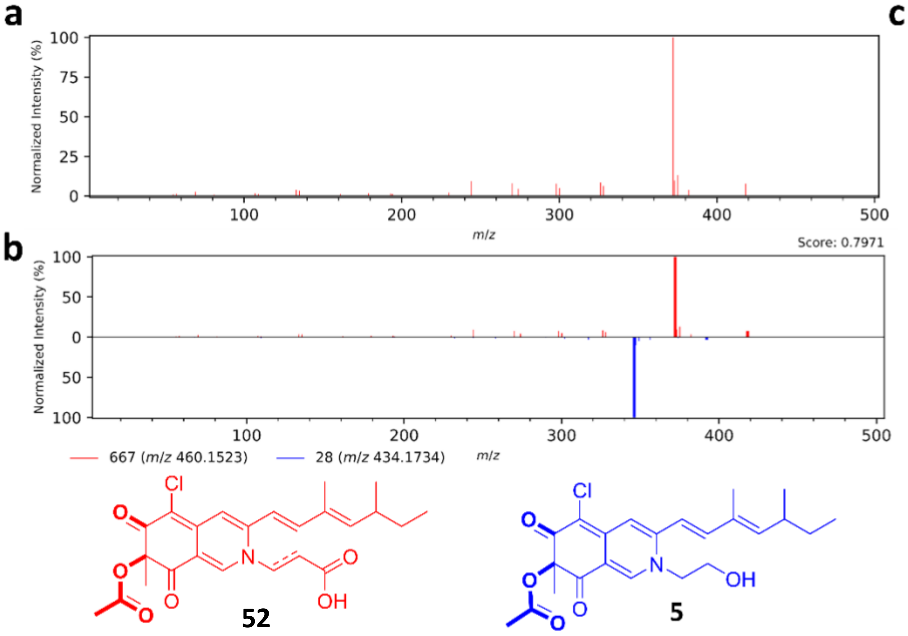

c

Fragment	Score
55.0537	0.0017
57.0696	0.0024
81.0704	0.0028
107.0856	0.0031
109.1013	0.0074
193.0293	0.0072
194.0354	0.0058
244.0529	0.0204

Neutral Loss	Score
**42.0098**	**0.0307**
77.9857	0.0208
85.0289	0.0481
87.0128	0.0572
**88.0152**	**0.5895**

Annotation explanation: Its mass difference from **51** or **61** is 2.0172 (H_2). This unsaturation could only be within N-chain as MS/MS spectra display typical acetylation loss.

Figure S56: MS/MS information of compound **53** (*m/z* 400.2125, −1.6 ppm, level 2) from molecular network. (**a**) MS/MS spectrum of compound **54**, (**b**) the mirror plot of MS/MS spectra from compound **53** against **5** with neutral losses from acylation loss (bold) (**c**) the common fragments and neutral losses with their contribution to cosine score.

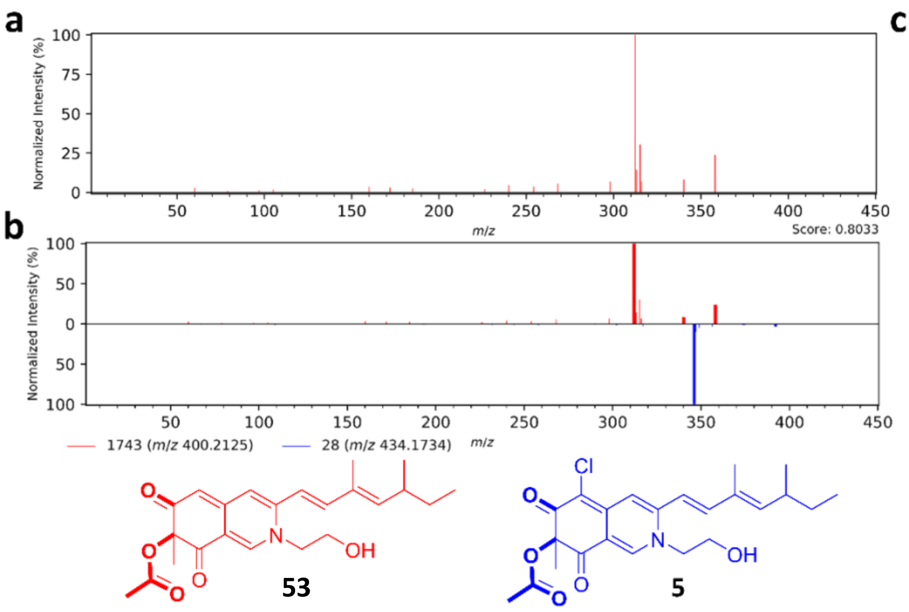

c

Neutral Loss	Score
**42.0117**	**0.0518**
**60.022**	**0.0178**
85.03	0.0706
87.0127	0.0672
**88.0165**	**0.5666**
132.0432	0.0191
240.1395	0.0102

Figure S57: MS/MS information of compound **54** (*m/z* 478.1635, −1.7 ppm, level 3) from molecular network. (**a**) MS/MS spectrum of compound **54**, (**b**) the mirror plot of MS/MS spectra from compound **54** against **5** with neutral losses from acylation loss (bold) (**c**) the common fragments and neutral losses with their contribution to cosine score.

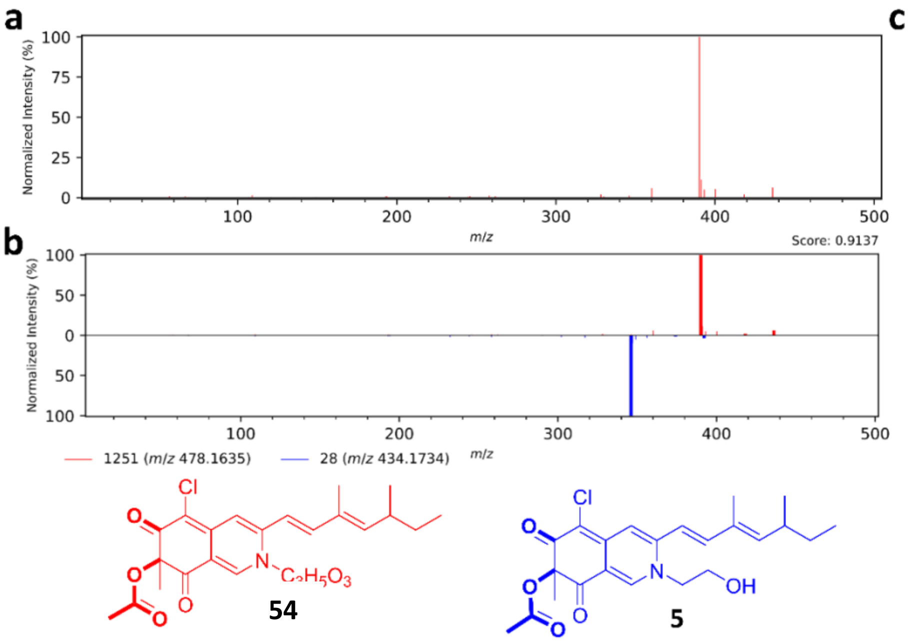

c

Fragment	Score
57.0692	0.002
67.0543	0.0052
81.0693	0.0024
107.0843	0.0019
109.101	0.01
193.0287	0.0067
194.037	0.0052
258.0682	0.01

Neutral Loss	Score
**42.0119**	**0.0322**
**60.0235**	**0.0112**
77.9885	0.0296
85.0298	0.0351
87.014	0.0721
**88.0165**	**0.6902**

Annotation explanation: Its calculated molecular formula was $C_{24}H_{28}ClNO_7$, because the molecule is acetylated, the moiety attached to the nitrogen has the deducted formula $C_3H_5O_3$.

Figure S58: MS/MS information of compound **55** (*m/z* 506.1576, 0.0 ppm, level 3) from molecular network. (**a**) MS/MS spectrum of compound **55**, (**b**) the mirror plot of MS/MS spectra from compound **55** against **5** with neutral losses from acylation loss (bold) (**c**) the common fragments and neutral losses with their contribution to cosine score.

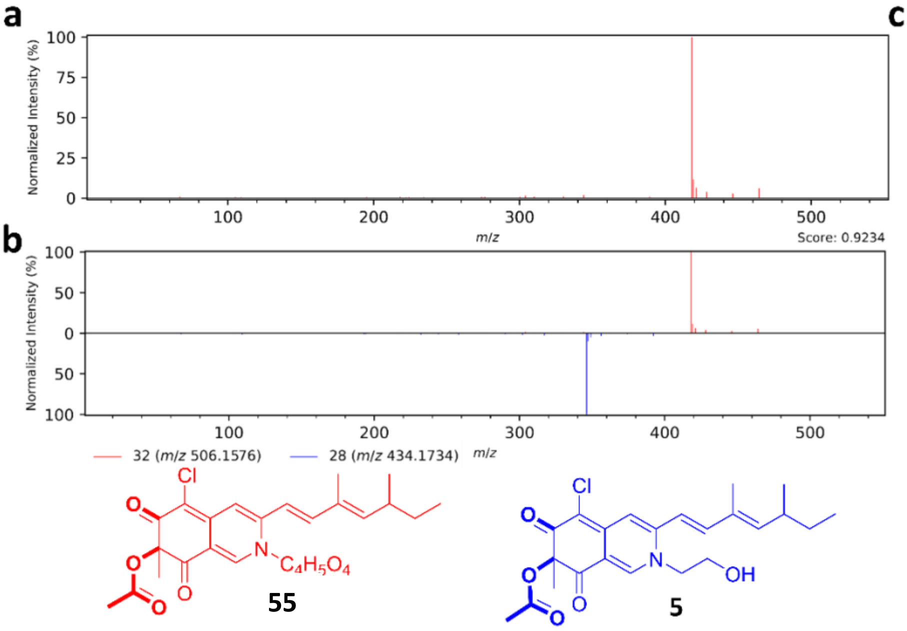

c

Fragment	Score
67.0533	0.0062
109.0998	0.0058

Neutral Loss	Score
**88.0163**	**0.7019**
87.0125	0.0748
85.0289	0.0401
**42.0118**	**0.0322**
77.9874	0.0254
**60.0223**	**0.013**
117.0576	0.0124
202.1236	0.0116

Annotation explanation: Its calculated molecular formula was $C_{25}H_{28}ClNO_8$, because the molecule is acetylated, the moiety attached to the nitrogen has the deducted formula $C_4H_5O_4$

Figure S59: MS/MS information of compound **56** (*m/z* 520.1742, −1.8 ppm, level 3) from molecular network. (**a**) MS/MS spectrum of compound **56**, (**b**) the mirror plot of MS/MS spectra from compound **56** against **5** with neutral losses from acylation loss (bold) (**c**) the common fragments and neutral losses with their contribution to cosine score.

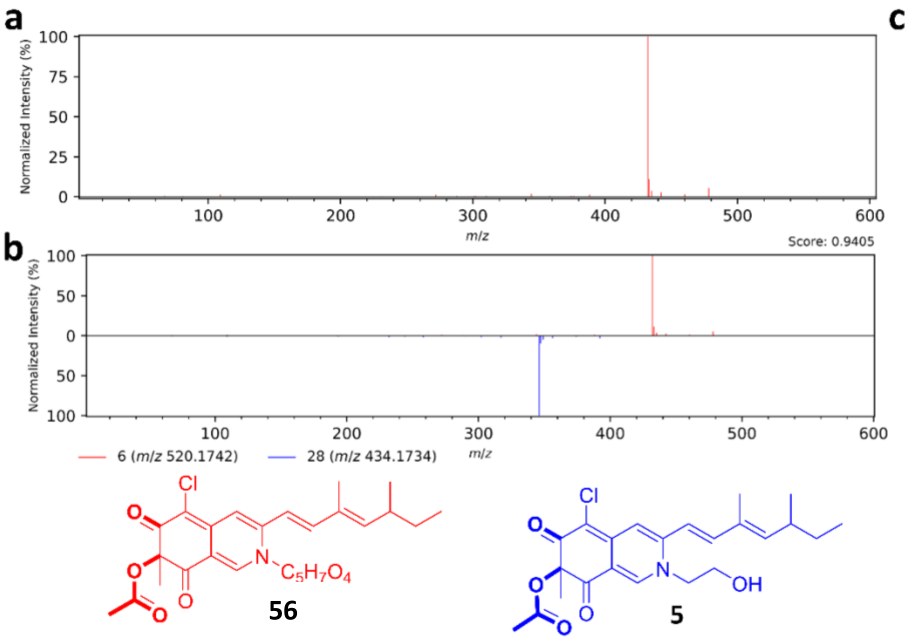

c

Fragment	Score
67.0547	0.0054
81.0694	0.0021
109.1023	0.0098
193.0323	0.0036
232.053	0.0052
258.0701	0.0054
302.1318	0.0073

Neutral Loss	Score
**42.0113**	**0.0316**
**60.0221**	**0.0097**
77.9874	0.0224
85.0285	0.0316
87.0126	0.0754
**88.0154**	**0.7264**
144.0781	0.0046

Annotation explanation: Its calculated molecular formula was $C_{26}H_{30}ClNO_8$, because the molecule is acetylated, the moiety attached to the nitrogen has the deducted formula $C_5H_7O_4$.

Figure S60: MS/MS information of compound **57** (*m/z* 505.1750, −2.8 ppm, level 3) from molecular network. (**a**) MS/MS spectrum of compound **57**, (**b**) the mirror plot of MS/MS spectra from compound **57** against **54** with neutral losses from acylation loss (bold) (**c**) the common fragments and neutral losses with their contribution to cosine score.

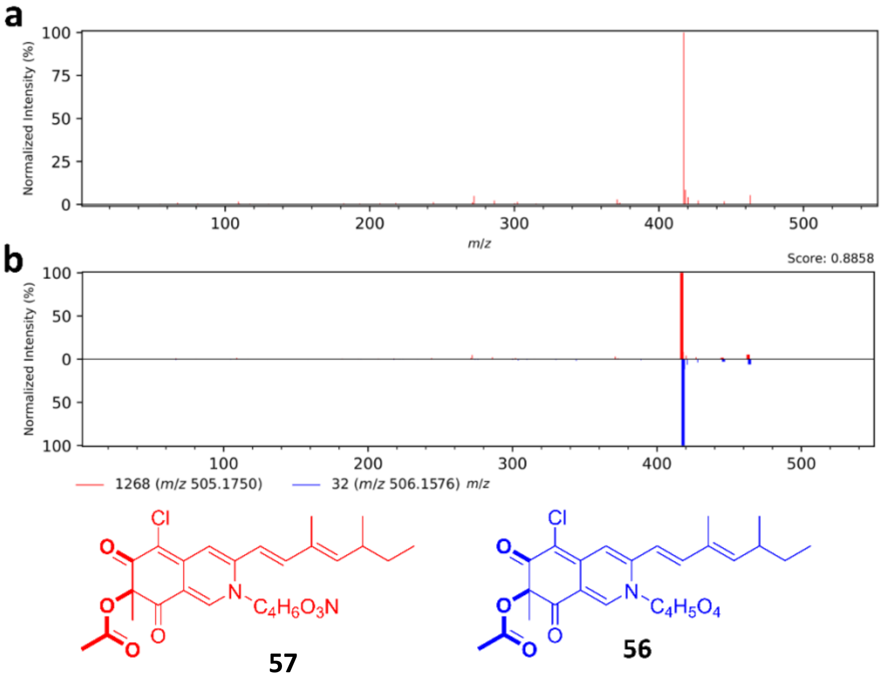

c

Fragment	Score
67.0544	0.0069
109.1006	0.006
218.0378	0.0055
300.1142	0.0052

Neutral Loss	Score
**42.012**	**0.0387**
**60.0221**	**0.0155**
77.9886	0.02
85.031	0.0356
87.0133	0.0678
**88.0167**	**0.6847**

Annotation explanation: Its calculated molecular formula was $C_{25}H_{29}ClN_2O_7$, because the molecule is acetylated, the moiety attached to the nitrogen has the deducted formula $C_4H_6O_3N$.

Figure S61: MS/MS information of compound **58** (*m/z* 519.1899, −1.2 ppm, level 3) from molecular network. (**a**) MS/MS spectrum of compound **58**, (**b**) the mirror plot of MS/MS spectra from compound **58** against **56** with neutral losses from acylation loss (bold) (**c**) the common fragments and neutral losses with their contribution to cosine score.

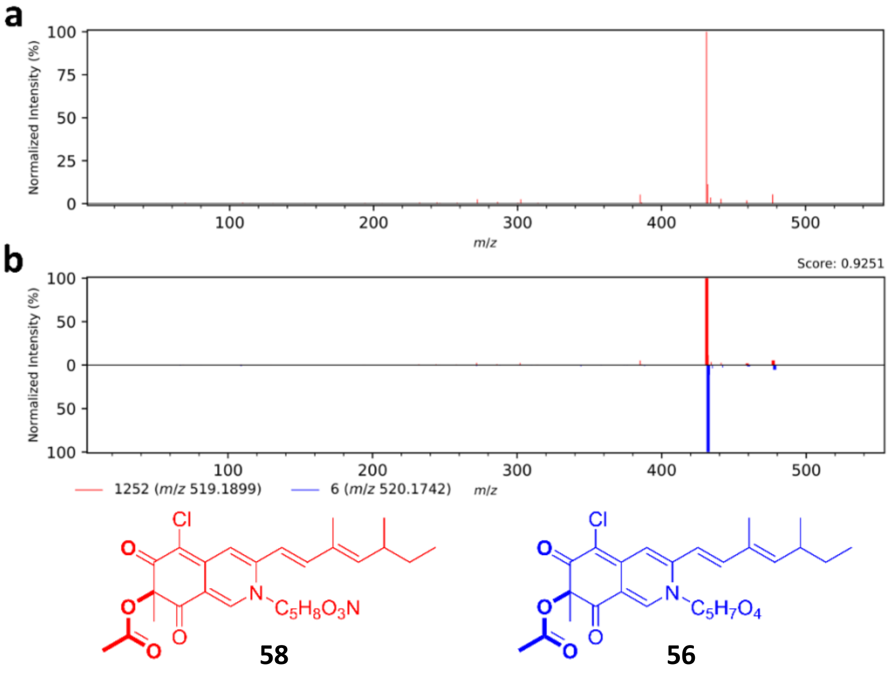

c

Fragment	Score
69.0702	0.0023
109.1007	0.0062
232.053	0.0032
258.0678	0.0029
272.0835	0.0125
302.1302	0.0081

Neutral Loss	Score
**42.0106**	**0.0384**
**60.0227**	**0.0118**
77.9884	0.02
85.0294	0.0258
87.013	0.0798
**88.0159**	**0.7141**

Annotation explanation: Its calculated molecular formula was $C_{26}H_{31}ClN_2O_7$, because the molecule is acetylated, the moiety attached to the nitrogen has the deducted formula $C_5H_8O_3N$.

Figure S62: MS/MS information of compound **59** (*m/z* 547.2219, −2.5 ppm, level 3) from molecular network. (**a**) MS/MS spectrum of compound **59**, (**b**) the mirror plot of MS/MS spectra from compound **59** against **58** with neutral losses from acylation loss from cluster B1 scaffold (bold) (**c**) the common fragments and neutral losses with their contribution to cosine score.

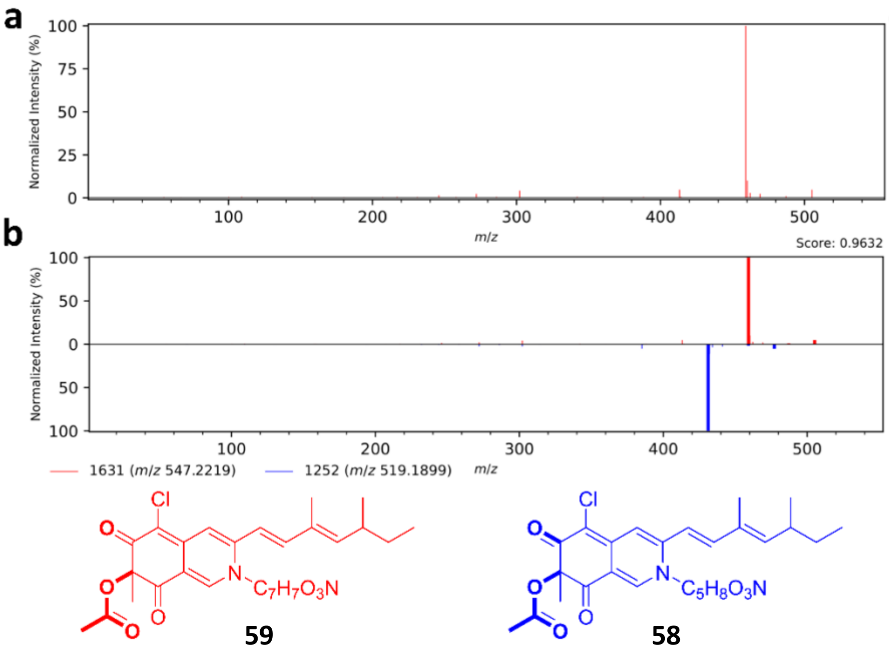

c

Fragment	Score
109.1009	0.0042
246.0318	0.0054
258.068	0.0037
272.0844	0.017
286.1016	0.0052
302.1309	0.023

Neutral Loss	Score
**42.0109**	**0.0353**
**60.0232**	**0.0094**
77.9872	0.0181
85.029	0.0224
87.0127	0.0747
**88.0161**	**0.701**
133.0209	0.0046
134.0227	0.0349
367.0504	0.0019
**389.1385**	**0.0023**

Annotation explanation: Its calculated molecular formula was $C_{28}H_{35}ClN_2O_7$, because the molecule is acetylated, the moiety attached to the nitrogen has the deducted formula $C_5H_7O_4$.

Figure S63: MS/MS information of compound **60** (*m/z* 561.2373, −2.0 ppm, level 3) from molecular network. (**a**) MS/MS spectrum of compound **60**, (**b**) the mirror plot of MS/MS spectra from compound **60** against **5** with neutral losses from acylation loss scaffold (bold) (**c**) the common fragments and neutral losses with their contribution to cosine score.

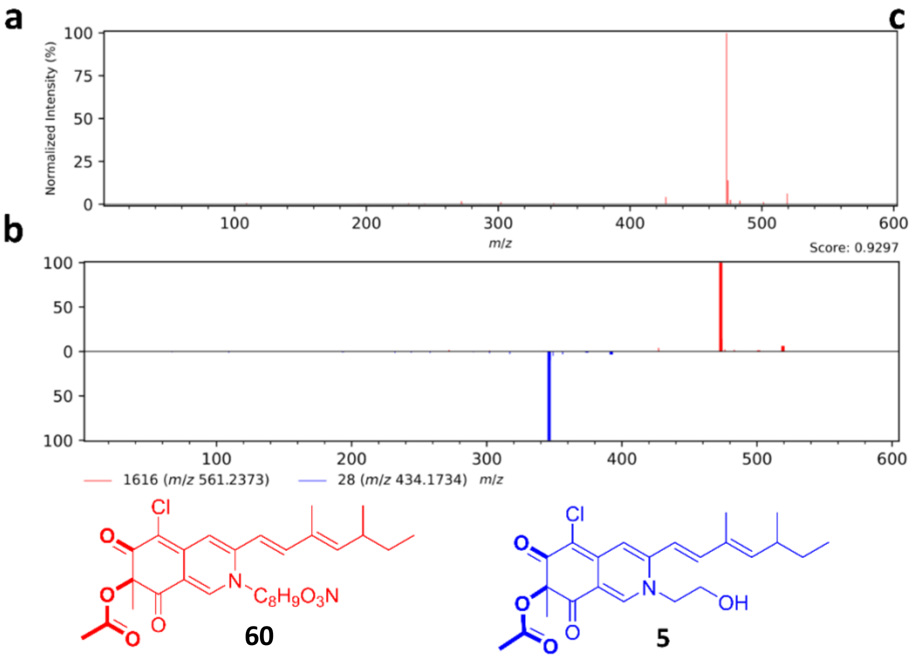

c

Fragment	Score
55.0534	0.0009
67.0545	0.0029
81.0705	0.0013
107.0853	0.0013
109.1017	0.0067
232.0523	0.0071
244.053	0.0056
302.1308	0.0107

Neutral Loss	Score
**42.0106**	**0.0336**
**60.0201**	**0.009**
77.9875	0.0193
85.0286	0.0257
87.0125	0.0845
**88.015**	**0.7211**

Annotation explanation: Its calculated molecular formula was $C_{29}H_{37}ClN_2O_7$, because the molecule is acetylated, the moiety attached to the nitrogen has the deducted formula $C_8H_9O_3N$.

Figure S64: MS/MS information of compound **61** (*m/z* 462.1695, −3.7 ppm, level 2) from molecular network. (**a**) MS/MS spectrum of compound **61**, (**b**) the mirror plot of MS/MS spectra from compound **61** against **6** with neutral losses from acylation and nitrogen chain loss (bold) (**c**) the common fragments and neutral losses with their contribution to cosine score.

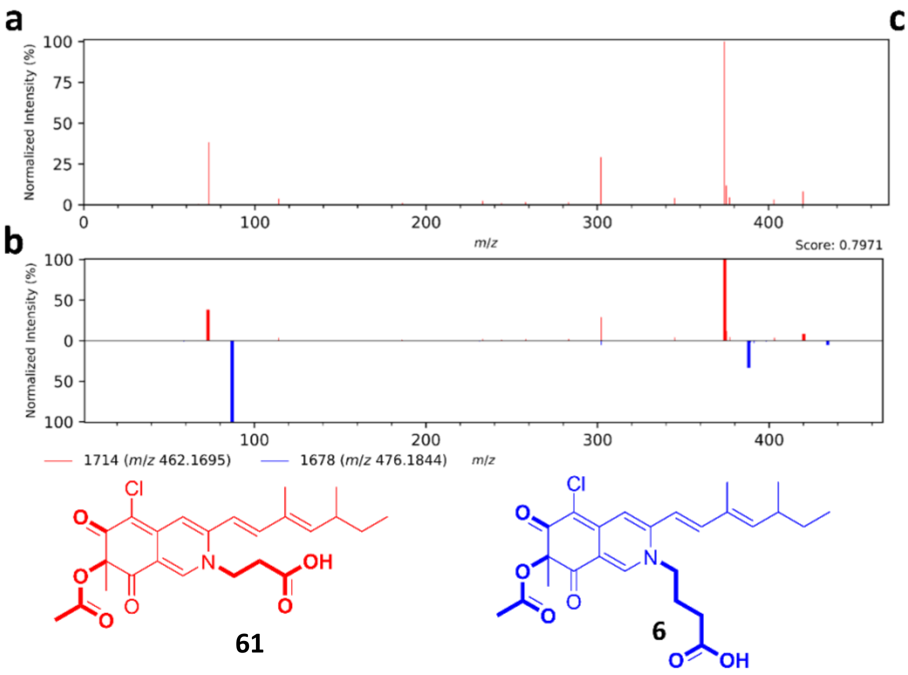

c

Fragment	Score
258.0692	0.0043
302.1305	0.0689

Neutral Loss	Score
**42.0122**	**0.0357**
85.031	0.0199
87.0147	0.0265
**88.0178**	**0.3113**
**389.1408**	**0.3306**

Figure S65: MS/MS information of compound **62** (*m/z* 500.1841, −1.3 ppm, level 3) from molecular network. (**a**) MS/MS spectrum of compound **62**, (**b**) the mirror plot of MS/MS spectra from compound **62** against **6** with neutral losses from acylation loss scaffold (bold) (**c**) the common fragments and neutral losses with their contribution to cosine score.

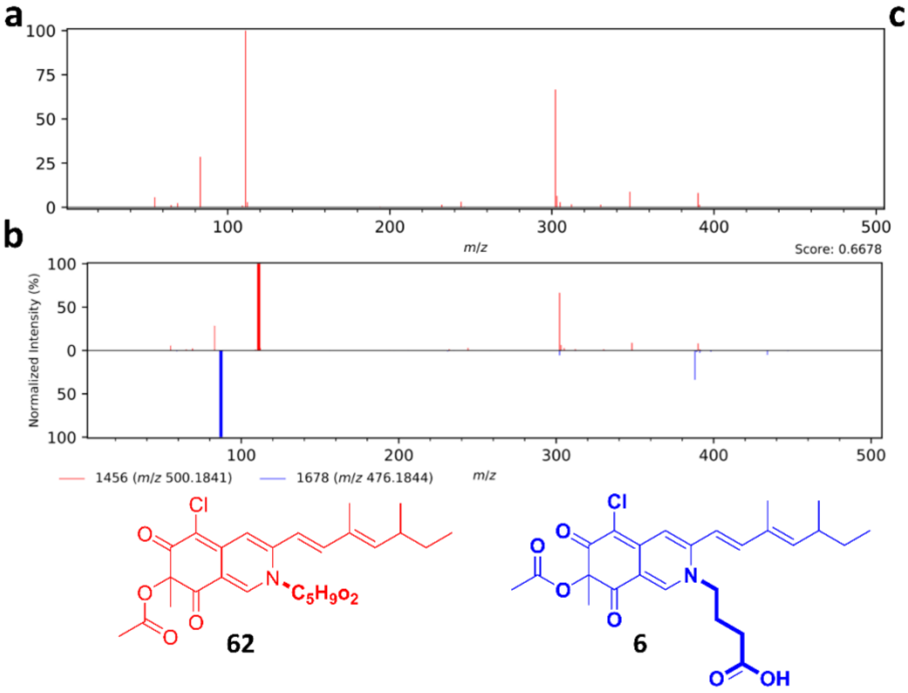

c

Fragment	Score
55.0541	0.0076
232.052	0.0042
302.1317	0.0974
303.1348	0.0099
312.16	0.0052
391.149	0.01

Neutral Loss	Score
388.1366	0.007
**389.1395**	**0.4999**
417.1346	0.0267

Annotation explanation: Its calculated molecular formula was $C_{27}H_{30}ClNO_6$, because the molecule is acetylated, the moiety attached to the nitrogen has the deducted formula $C_6H_7O_2$.

.

Figure S66: MS/MS information of compound **63** (*m/z* 451.1522, −0.9 ppm, level 0) from molecular network. (**a**) MS/MS spectrum of compound **63**, (**b**) the mirror plot of MS/MS spectra from compound **63** against **15** with the main contributors to cosine score (bold), and (**c**) the fragment list and their contribution to cosine score.

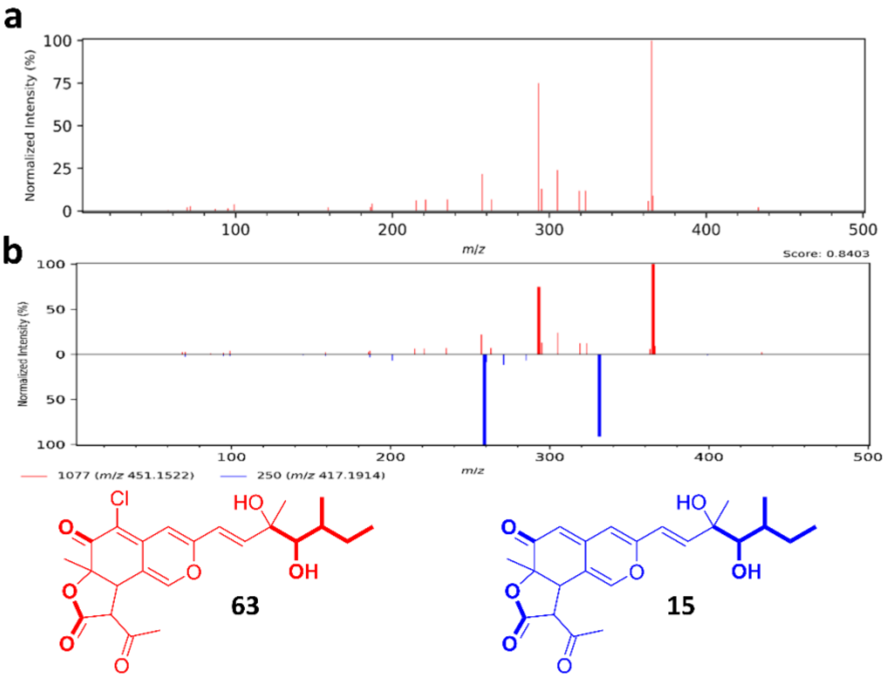

c

Fragment	Score
57.0696	0.0013
69.0702	0.0044
71.0496	0.0098
87.0805	0.0029
95.0496	0.0058
99.0449	0.0092
159.0808	0.0075

Neutral Loss	Score
18.0088	0.0059
85.0686	0.0284
**86.0716**	**0.3337**
132.0777	0.0315
146.0934	0.0578
**158.0566**	**0.3021**
216.0989	0.0242
230.1146	0.0159

Figure S67: MS/MS information of compound **64** (*m/z* 391.2121, −1.5 ppm, level 3) from molecular network. (**a**) MS/MS spectrum of compound **64**, (**b**) the mirror plot of MS/MS spectra from compound **64** against **14** with neutral losses from CO-OH and diol carbon chain (bold) (**c**) the common fragments and neutral losses with their contribution to cosine score.

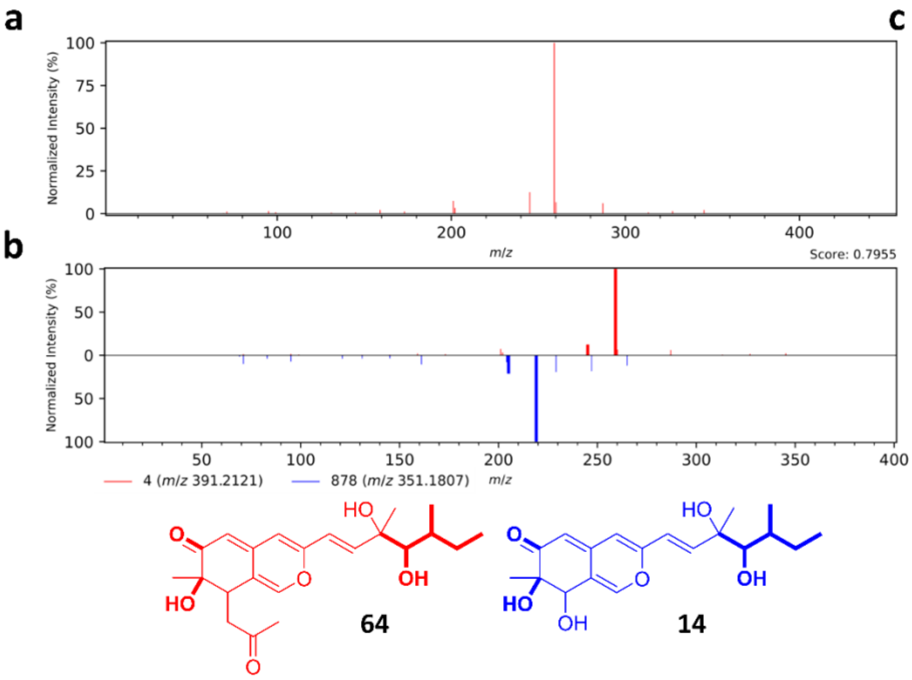

c

Fragment	Score
71.0492	0.0192
95.0494	0.0178
131.0856	0.0083
145.0649	0.0084

Neutral Loss	Score
**132.079**	**0.5451**
146.0947	0.0892
104.0843	0.0581
190.1209	0.0486
18.0107	0.001

Annotation explanation: the diol chain and the position of the modification were positionned thanks to 132.079 Da neutral loss. The more plausible acetone position was favoured. This azaphilone motive is also reported within litterature. [4]

Figure S68: MS/MS information of compound **65** (*m/z* 425.1731, −1.3 ppm, level 3) from molecular network. (**a**) MS/MS spectrum of compound **65**, (**b**) the mirror plot of MS/MS spectra from compound **65** against **64** with neutral losses from CO-OH and diol carbon chain (bold) (**c**) the common fragments and neutral losses with their contribution to cosine score.

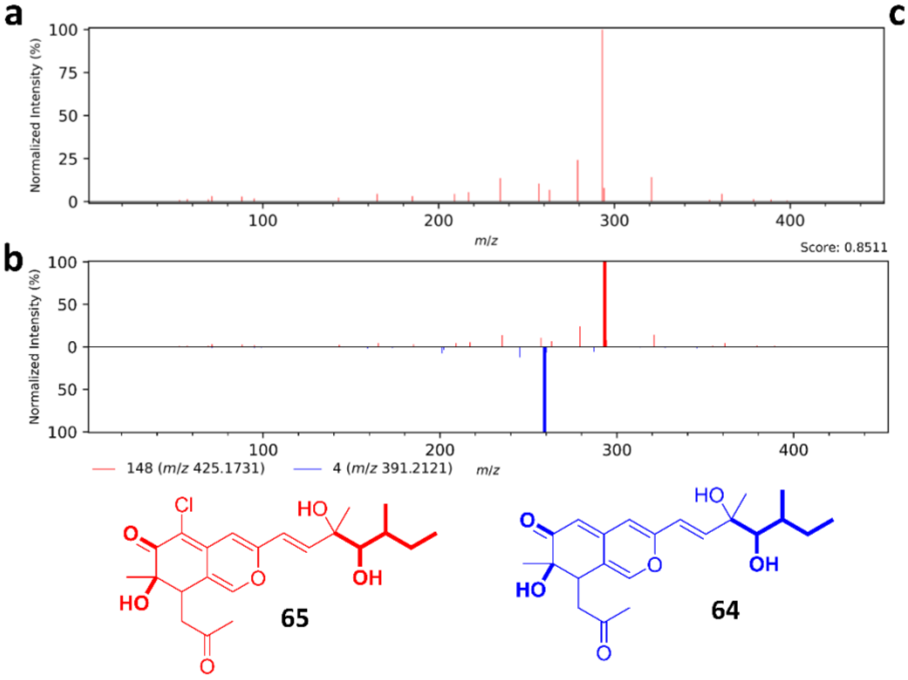

c

Fragment	Score
69.0695	0.0036
71.0482	0.0109
95.0501	0.0092

Neutral Loss	Score
46.0051	0.0097
64.0188	0.014
104.0835	0.0513
131.0768	0.04
**132.0789**	**0.5516**
146.095	0.0961
190.1232	0.0557
260.1276	0.0089

Annotation explanation: Its *m/z* difference with **65** was 33.9610, and its lack of ^{37}Cl isotope

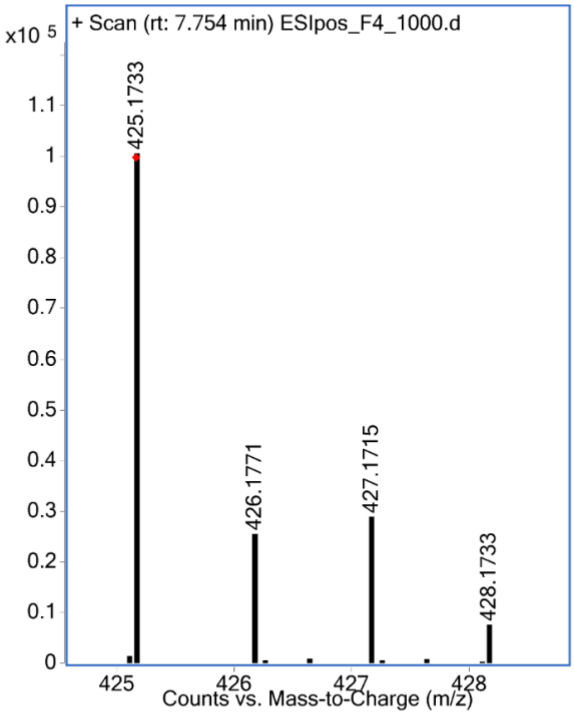

Figure S69: MS/MS information of compound **66** (*m/z* 393.2274, −0.6 ppm, level 3) from molecular network. (**a**) MS/MS spectrum of compound **66**, (**b**) the mirror plot of MS/MS spectra from compound **66** against **64** with neutral losses from CO-OH and diol carbon chain (bold) (**c**) the common fragments and neutral losses with their contribution to cosine score.

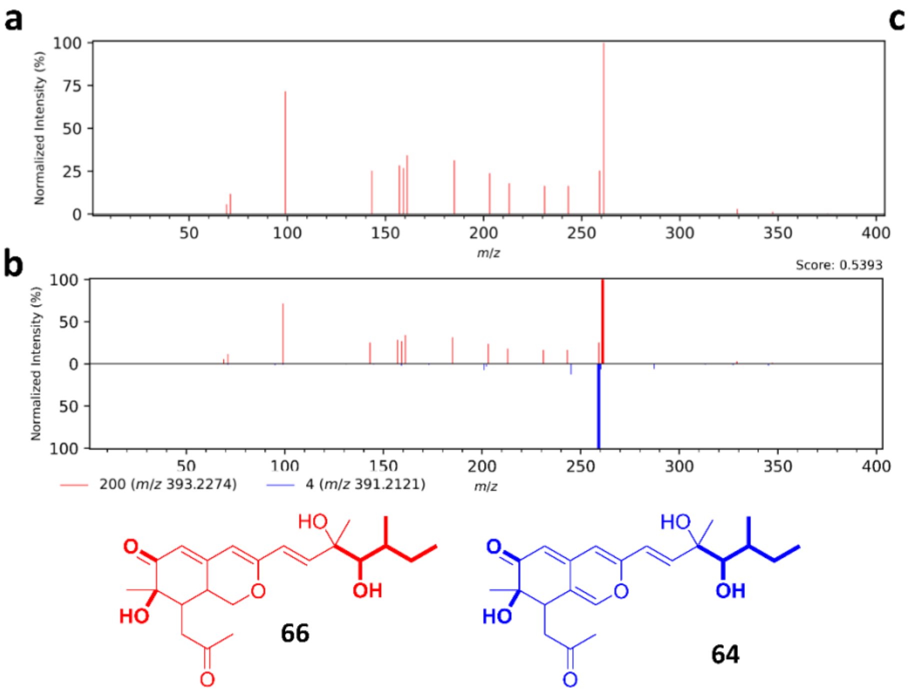

c

Fragment	Score
69.0704	0.0052
71.0496	0.0149
99.0447	0.0295

Neutral Loss	Score
46.0019	0.0069
64.0155	0.008
**132.0777**	**0.3894**
190.1212	0.0519
232.1309	0.0335

Annotation explanation: It clusterizes with **16**, **17** and **18** and its *m/z* difference of 2.0153 (corresponding to H_2 loss) from **64**.

Figure S70: MS/MS information of compound **67** (*m/z* 350.1964, −0.6 ppm, level 2) from molecular network. (**a**) MS/MS spectrum of compound **67**, (**b**) the mirror plot of MS/MS spectra from compound **67** against **14** with neutral losses from CO-OH and diol carbon chain (bold) (**c**) the common fragments and neutral losses with their contribution to cosine score.

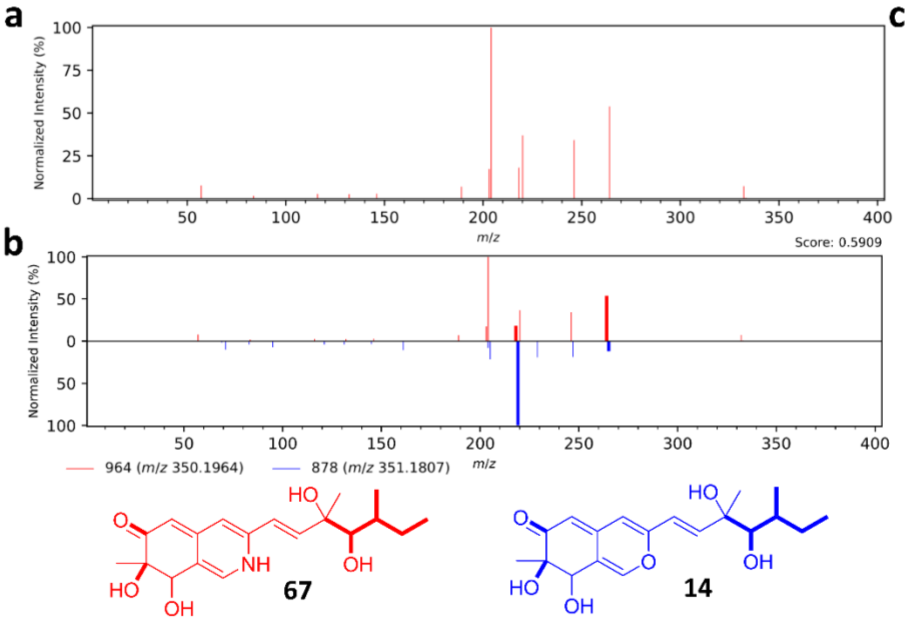

c

Neutral Loss	Score
18.0076	0.0048
**86.0732**	**0.0985**
104.0828	0.098
**132.0793**	**0.1648**
146.0945	0.1791
147.1015	0.0456

Annotation explanation: Its *m/z* difference with **14** was 0.9843, indicates the presence of NH instead of O atom.

Figure S71: MS/MS information of compound **68** (*m/z* 390.2282, −1.8 ppm, level 3) from molecular network. (a) MS/MS spectrum of compound **68**, (**b**) the mirror plot of MS/MS spectra from compound **68** against **64** with neutral losses from CO-OH and diol carbon chain (bold) (**c**) the common fragments and neutral losses with their contribution to cosine score.

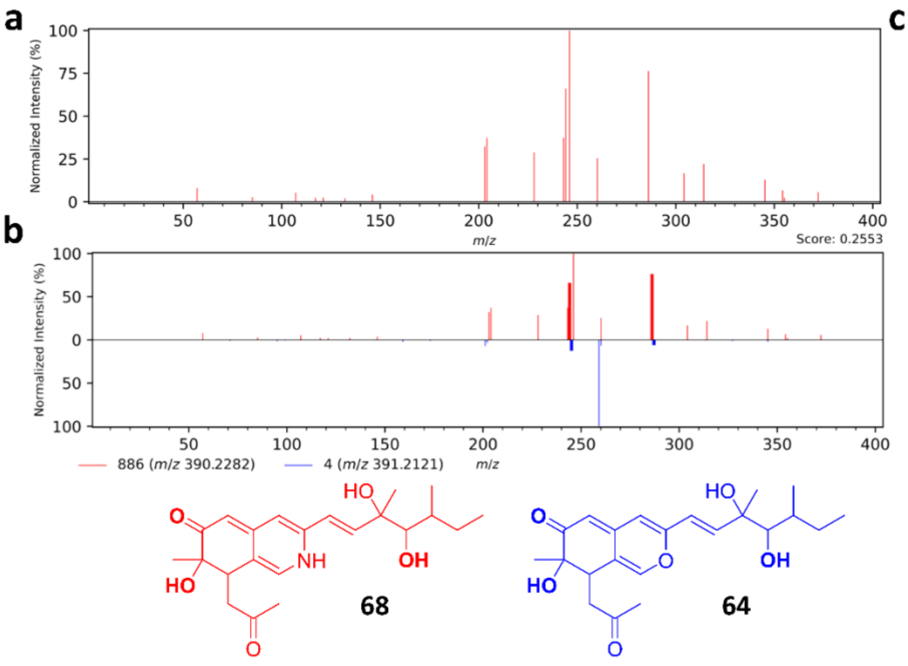

c

Fragment	Score
260.1284	0.0478
345.2049	0.0194

Neutral Loss	Score
18.0129	0.0034
104.0839	0.0791
146.0947	0.1057

Annotation explanation: Its *m/z* difference with **64** was 0.9843, indicates the presence of NH instead of O atom.

Figure S72: MS/MS information of compound **69** (*m/z* 339.1963, −2.5 ppm, level 3) from molecular network.

(a) MS/MS spectrum of compound **69**, (**b**) the mirror plot of MS/MS spectra from compound **69** against **3** with common fragment (**c**) the common fragments with their contribution to cosine score.

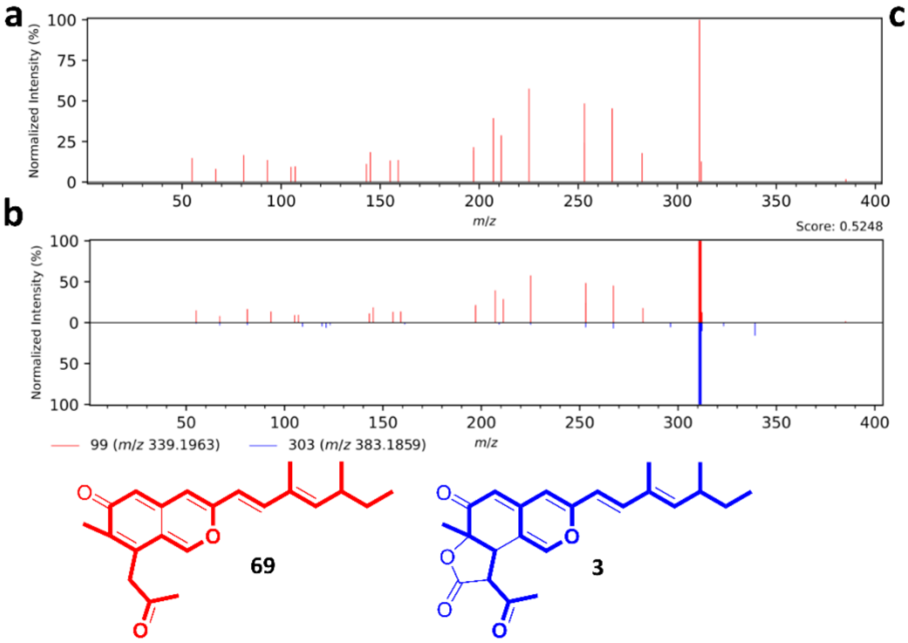

c

Fragment	Score
55.0538	0.0132
67.0532	0.0167
81.0692	0.0215
225.1271	0.0385
253.1221	0.0367
267.139	0.0558
**311.2006**	**0.3069**
312.2048	0.0354

Annotation explanation: The main common fragment with **3** has *m/z* 311.2006 and it corresponds to a CO loss from **69** aromatic ring.

Figure S73: MS/MS information of compound **70** (*m/z* 399.1805, −0.7 ppm, level 3) from molecular network. (**a**) MS/MS spectrum of compound **70**, (**b**) the mirror plot of MS/MS spectra from compound **70** against **3** with common neutral loss from lactone ring and CO (**c**) the common fragments with their contribution to cosine score.

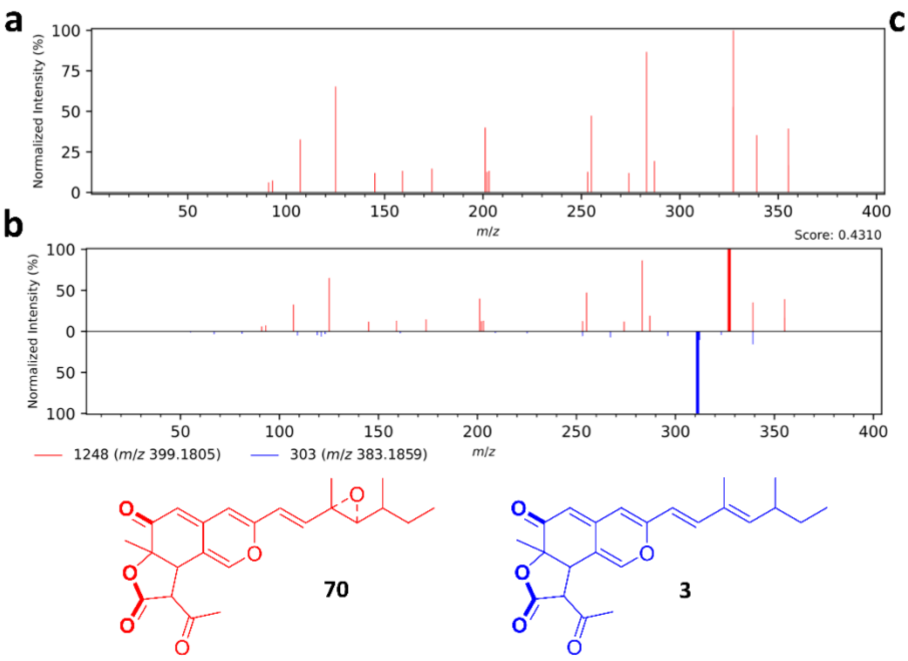

c

Fragment	Score
253.1225	0.0248
339.124	0.0679

Neutral Loss	Score
**71.9836**	**0.2865**
274.0843	0.0518

Annotation explanation: the fragmentation from lactone lactone ring followed by CO loss were common with **3**, and its *m/z* difference was 15.9946 (corresponding to O).

Figure S74: MS/MS information of compound **71** (*m/z* 433.1419, −1.5 ppm, level 3) from molecular network.(**a**) MS/MS spectrum of compound **71**, (**b**) the mirror plot of MS/MS spectra from compound **71** against **4** with common neutral loss from lactone ring and CO (**c**) the common fragments with their contribution to cosine score.

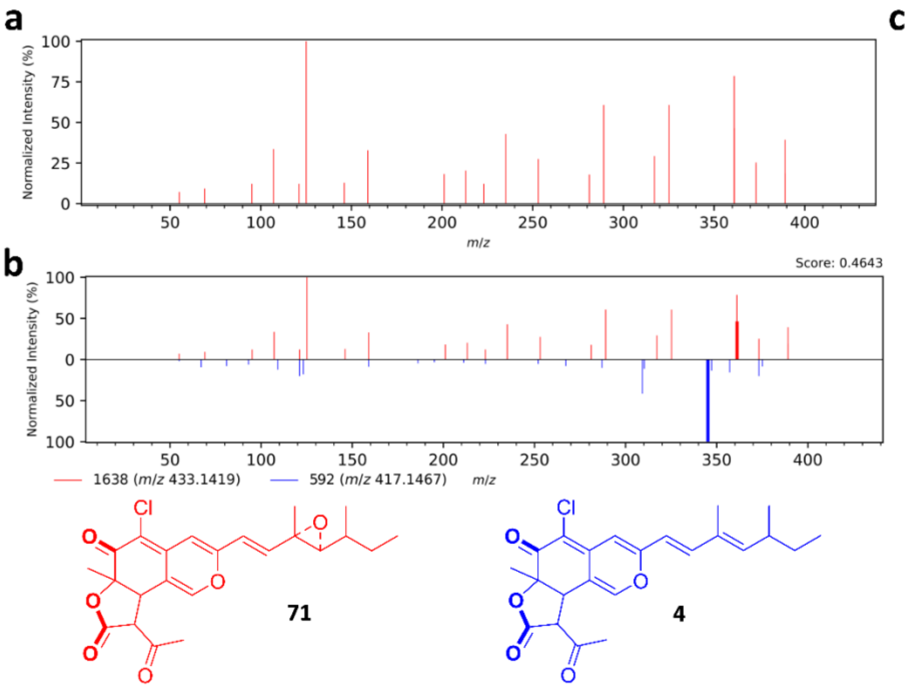

c

Fragment	Score
55.0537	0.008
121.1022	0.0313
159.0798	0.0341
373.0827	0.044

Neutral Loss	Score
60.0205	0.0399
**71.9844**	**0.1362**
107.9625	0.1004
308.0459	0.0703

Annotation explanation: The fragmentation from lactone ring followed by CO loss were common with **4**, and its *m/z* difference was 15.9946 (corresponding to O).

Figure S75: MS/MS information of compound **72** (*m/z* 381.1691, 1.5 ppm, level 2) from molecular network. (a) MS/MS spectrum of compound **72**, (**b**) the mirror plot of MS/MS spectra from compound **72** against **23** (**c**) the common fragments and neutral losses with their contribution to cosine score.

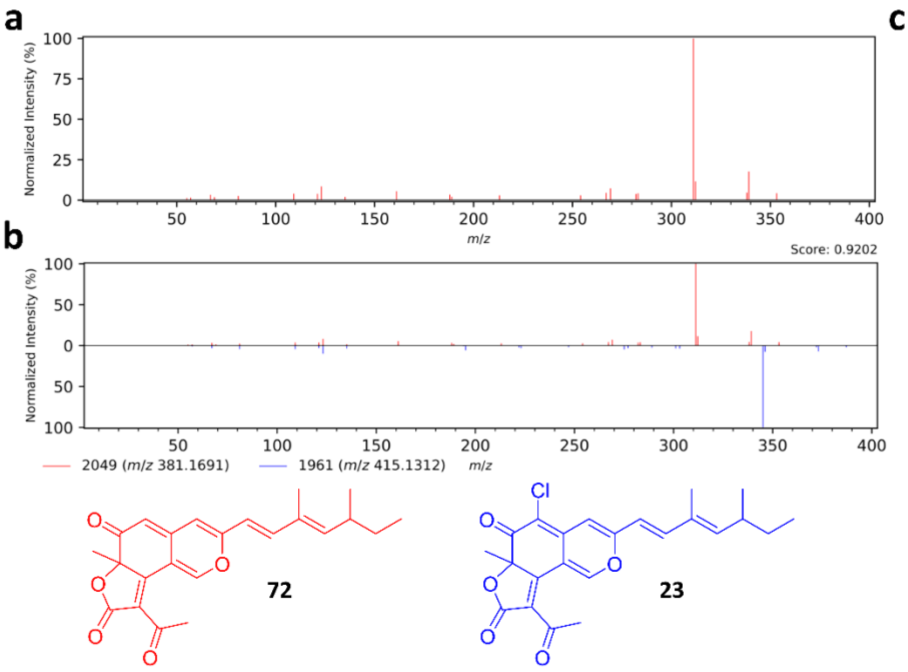

c

Fragment	Score
57.0698	0.0068
67.0538	0.0168
81.0699	0.0173
109.1011	0.0215
121.1011	0.0175
123.1171	0.048
135.1169	0.0139

Neutral Loss	Score
27.9945	0.0147
42.01	0.0595
43.0178	0.0156
69.0014	0.0491
70.0048	0.5222
112.0157	0.0269
114.0678	0.0211
168.0787	0.0122
192.1153	0.0137
193.1226	0.0144
220.1097	0.029

Annotation explanation: Its *m/z* differences with **23** of 33.9621 and the lack of typical ^{37}Cl isotopic peak indicating a H atom instead of a Cl.

Figure S76: MS/MS information of compound **73** (*m/z* 385.2017, −2.0 ppm, level 2) from molecular network. (**a**) MS/MS spectrum of compound **73**, (**b**) the mirror plot of MS/MS spectra from compound **73** against **3** with common neutral loss from lactone ring and CO (**c**) the common fragments and neutral losses with their contribution to cosine score.

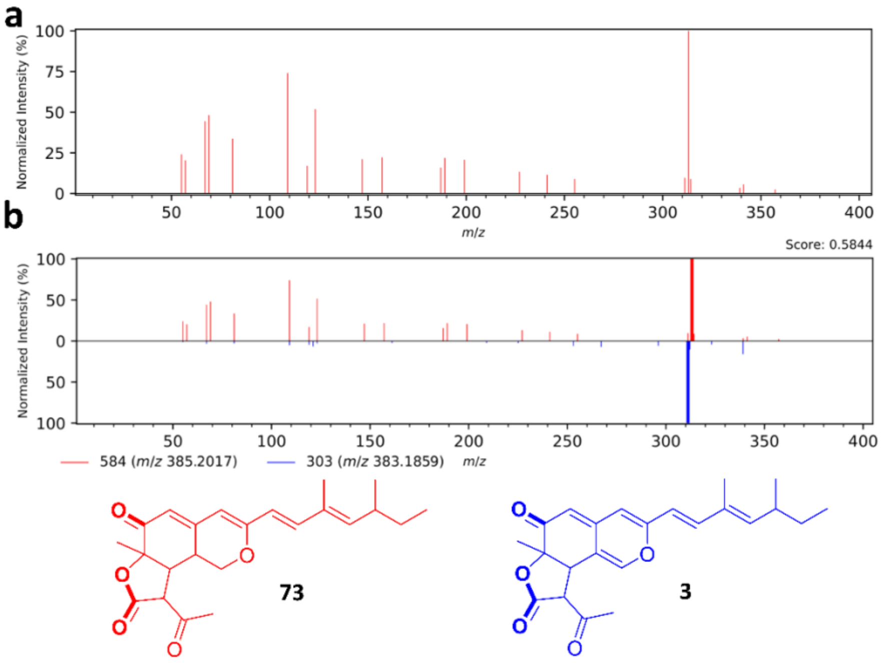

c

Fragment	Score
55.0539	0.0165
81.07	0.03
109.1013	0.0579
119.0857	0.0265
784.3416	0.0002

Neutral Loss	Score
44.0628	0.0283
70.9816	0.029
**71.9847**	**0.3007**
262.0846	0.0556
316.1316	0.0398

Annotation explanation: Its *m/z* difference of 2.0153 (corresponding to H_2) from **3** and its lactone ring fragmentation.

Figure S77: MS/MS information of compound **74** (*m/z* 380.1861, −1. 2 ppm, level 0) from molecular network. (**a**) MS/MS spectrum of compound **74**, (**b**) the mirror plot of MS/MS spectra from compound **74** against **3** with common neutral loss from lactone ring and CO (**c**) the common fragments and neutral losses with their contribution to cosine score.

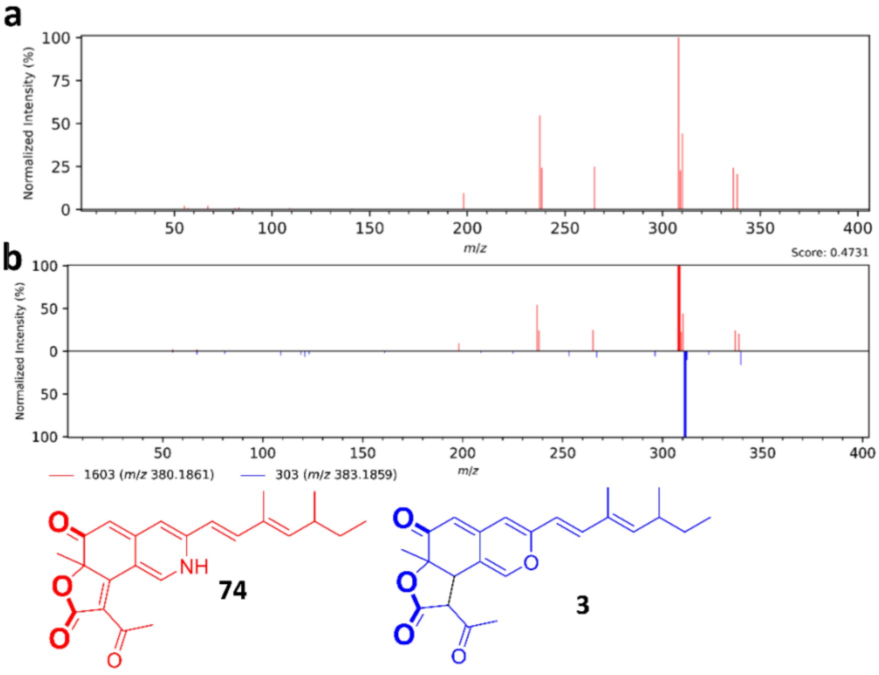

c

Fragment	Score
55.0545	0.0064
67.054	0.011
81.0682	0.006
109.1007	0.008

Neutral Loss	Score
71.0136	0.0442
**71.9847**	**0.3974**

Annotation explanation: Compound **74** possesses a neutral loss from its lactone ring fragmentation. The NH conversion from O was suggested by its even *m/z*.

Figure S78: MS/MS information of compound **75** (*m/z* 424.2123, −1.1 ppm, level 0) from molecular network. (**a**) MS/MS spectrum of compound **75**, (**b**) the mirror plot of MS/MS spectra from compound **75** against **74** with common neutral loss from lactone ring and CO (**c**) the common fragments and neutral losses with their contribution to cosine score.

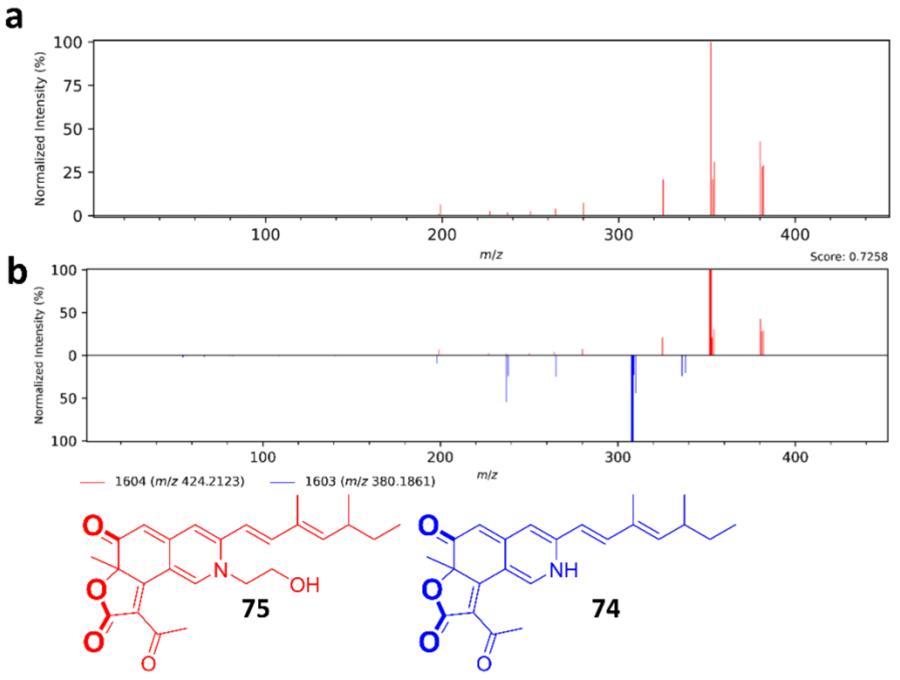

c

Fragment	Score
55.0549	55.0545
67.0545	67.054
198.092	198.0917
237.1151	237.1151

Neutral Loss	Score
42.0103	0.0771
43.9893	0.1014
70.0055	0.1167
71.0131	0.0689
**71.984**	**0.3161**

Annotation explanation: Its lactone ring fragmentation, its even *m/z* and the mass difference of *m/z* 44.0263 with **74** (correspondind to C_2H_4O)

Figure S79: MS/MS information of compound **76** (*m/z* 357.2065, −1.3 ppm, level 2) from molecular network. (**a**) MS/MS spectrum of compound **76**), (**b**) the mirror plot of MS/MS spectra from compound **76** against **64** with common neutral loss from CO and OH (**c**) the common fragments and neutral losses with their contribution to cosine score.

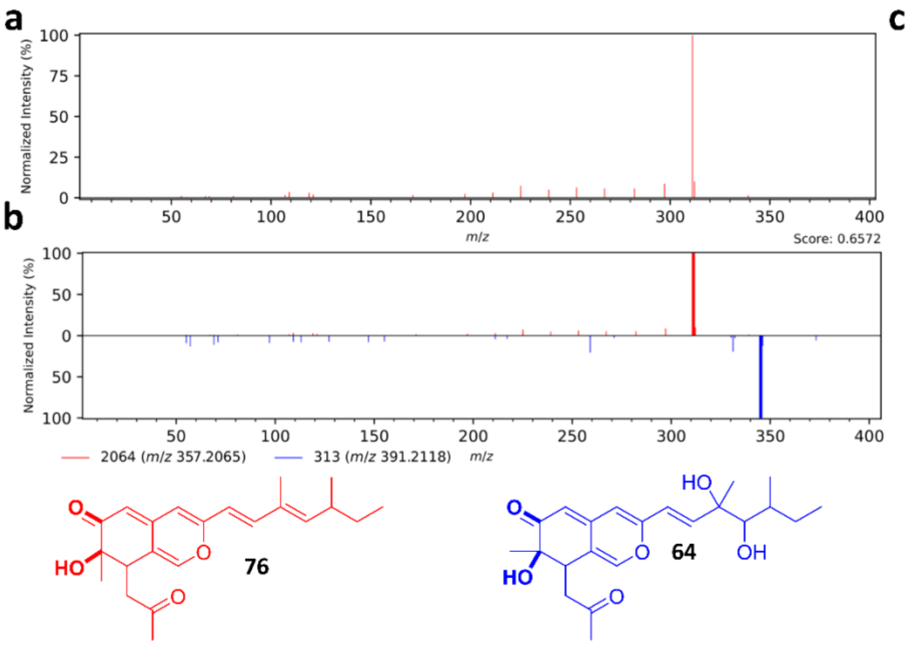

c

Fragment	Score
55.0538	0.0146
109.1016	0.0232

Neutral Loss	Score
**46.0055**	**0.4573**
60.021	0.0597
45.0018	0.0509
132.0787	0.0207
236.1049	0.0171
18.0106	0.0137

Annotation explanation: it mass difference from compound **64** was 34.0056 (corresponding to H_2O_2) as support the lack of diol of compound **76**. Moreover compound **76** was already described and was produced by genetically close strain of *Penicillium* sp. [4]

Figure S80: MS/MS information of compound **77** (*m/z* 391.1674, −0.9 ppm, level 3) from molecular network. (**a**) MS/MS spectrum of compound **77**, (**b**) the mirror plot of MS/MS spectra from compound **77** against **76** with common neutral loss from CO and OH (**c**) the common fragments and neutral losses with their contribution to cosine score.

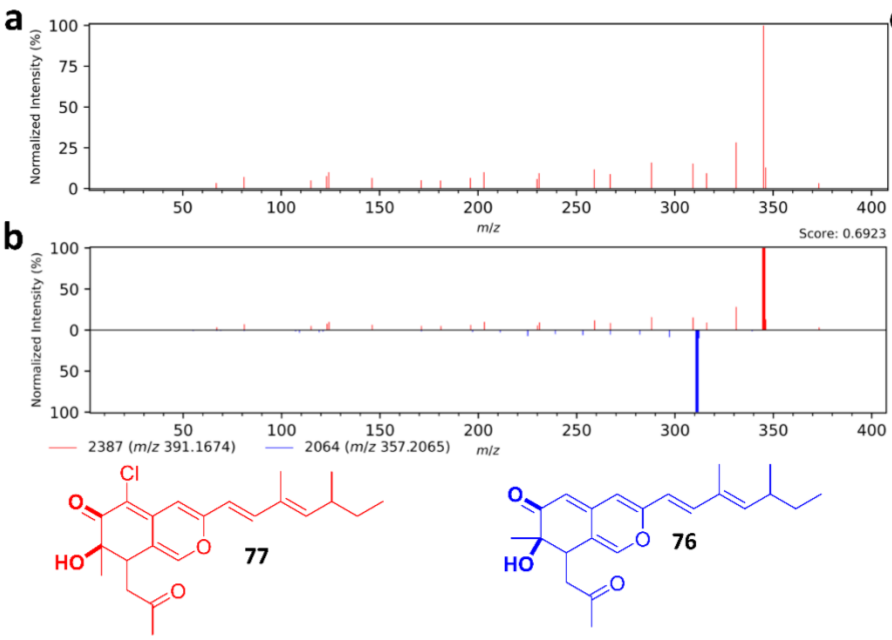

c

Fragment	Score
67.0549	0.0082
81.068	0.0128
171.1127	0.013

Neutral Loss	Score
45.0011	0.0505
**46.0064**	**0.4436**
60.0217	0.0695
75.0452	0.0324
132.0787	0.0414
160.1131	0.021

Annotation explanation: Its *m/z* difference with compound **76** was 33.9608. Moreover compound **77** was already described produced altogether with **76** by genetically close strain of *Penicillium* sp. [4]

Figure S81: MS/MS information of compound **78** (*m/z* 373.2016, −1.8 ppm, level 2) from molecular network. (**a**) MS/MS spectrum of compound **78**, (**b**) the mirror plot of MS/MS spectra from compound **78** against **64** with common neutral loss from CO and OH (**c**) common fragment and neutral loss with their contribution to cosine score.

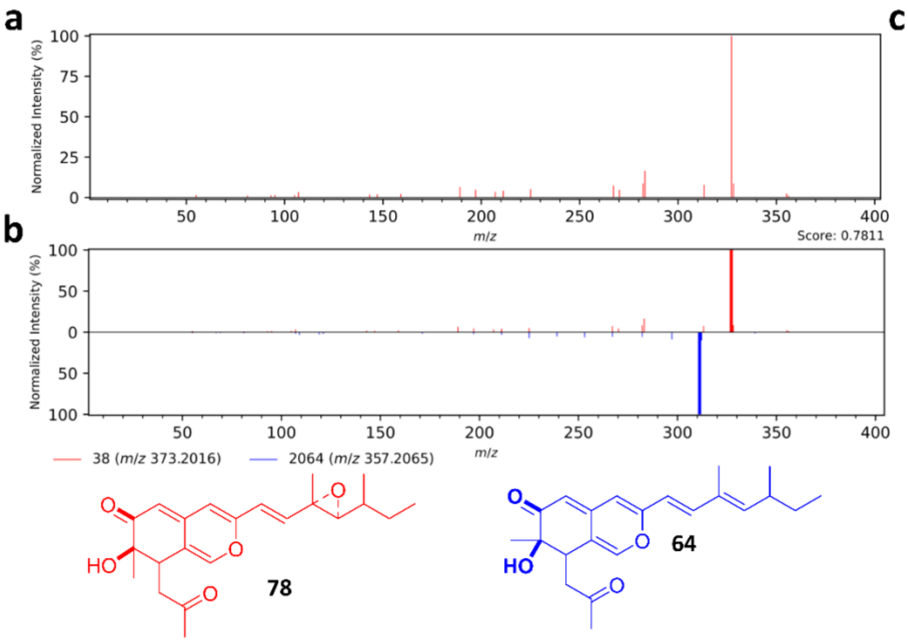

c

Fragment	Score
55.054	0.0068
81.0701	0.0062
107.085	0.0126
211.1488	0.0198
225.1264	0.0328
267.1382	0.0342
282.1608	0.0371

Neutral Loss	Score
18.0115	0.0101
45.0019	0.0493
**46.0057**	**0.5286**
60.0217	0.0436

Annotation explanation: compound **78** possesses a neutral loss of 46.0057 Da(corresponding to CO + H_2O) and its *m/z* diffrence from **64** is 15.9951 (corresponding to O).

Figure S82: MS/MS information of compound **79** (*m/z* 407.1627, −1.8 ppm, level 2) from molecular network. (a) MS/MS spectrum of compound **79**, (**b**) the mirror plot of MS/MS spectra from compound **79** against **78** with common neutral loss from CO and OH (**c**) common fragment and neutral loss with their contribution to cosine score.

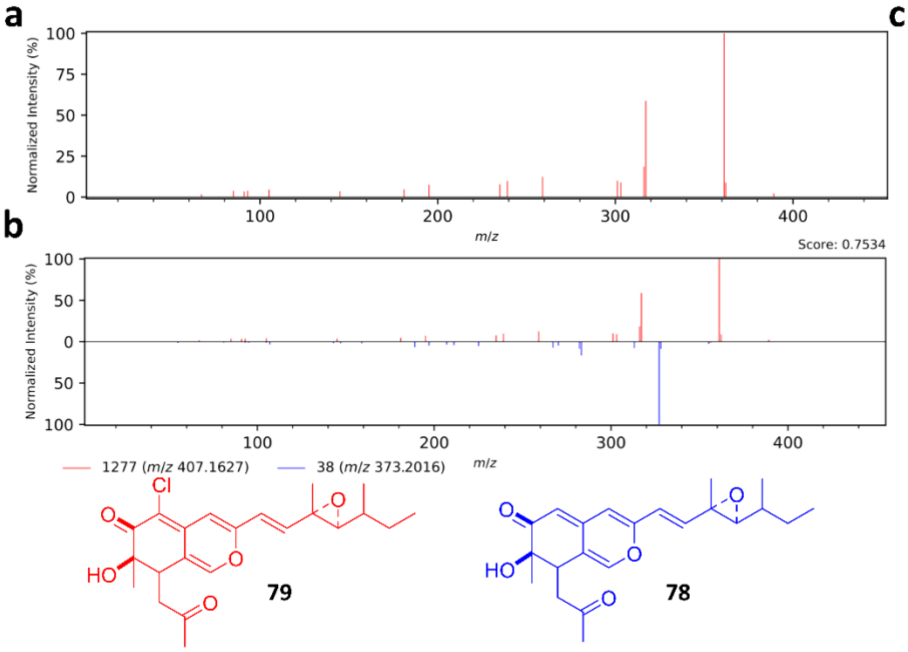

c

Fragment	Score
93.07	0.0098
105.0698	0.0109

Neutral Loss	Score
18.0126	0.0102
45.0017	0.0375
**46.005**	**0.4268**
90.0318	0.133
91.0401	0.0542
106.0636	0.0367
148.0732	0.0343

Annotation explanation: compound **79** possesses a neutral loss of 46.0057 Da (corresponding to CO + H_2O) and its *m/z* diffrence from **79** is 15.9951 (corresponding to O).

TableS1: ^{1}H NMR spectroscopic data for compounds **1**, **2**, **5**, **23, 75, 63, 80 and 74**

Position	δH (*J* in Hz)							
	**1**	**2**	**5**	**23**	**75**	**63**	**80**	**74**
	$CDCl_3$	$CDCl_3$	$CDCl_3$	MeOD	$CDCl_3$	$CDCl_3$	DMF-*d7*	$CDCl_3$
1	7.90, s	7.93, s	7.83, s	8.88, s	7.91, s	7.42, s	8.10, s	9.32, s
3								
4	6.61, s	6.86, s	6.99, s	6.81, s	6.77, s	6.56, s	7.00, s	6.8, s
5					6.57, s			6.78, s
8						3.84, d(12.5)		
9	6.51, d(15.9)	6.13, d(16.1)	6.23, d(15.9)	6.35, d(15.6)	6.28, d(15.5)	6.41, d(15.5)	6.70, d(15.8)	6.28, d(16.4)
10	7.03, d(15.9)	7.04, d(16.7)	6.91, d(15.6)	7.19, d(16.0)	6.95, d(15.5)	6.65, d(15.7)	7.23, d(15.7)	7.57, d(16.8)
11								
12	5.67, (d10.0)	5.69, d(9.9)	5.68, d(9.9)	5.77, d(9.8)	5.70, d(9.5)	3.49, d(1.2)	5.87, d(9.5)	5.82, d(10.4)
13	2.45, m	2.47, m	2.47, m	2.52, m	2.47, m	1.7, m	2.53, m	2.49, m
14	1.39/1.29, m	1.40/1.30, m	1.42/1.32, m	1.46/1.34, m	1.42/1.32, m	1.39/1.31, m	1.43/1.34, m	1.43/1.34, m
15	0.83, t(7.5)	0.84, t(7.5)	0.86, t(7.5)	0.89, d(7.6)	0.86, t(7.4)	0.9, t(7.4)	0.87, t(7.4)	0.86, t(7.4)
16	0.98, d(6.8)	0.99, d(6.6)	1.00, d(6.7)	1.03, d(6.6)	1.00, d(6.8)	0.95, d(6.7)	1.01, d(6.6)	0.99, d(6.7)
17	1.81, s	1.83, s	1.82, s	1.88, s	1.86, s	1.34, s	1.92, s	1.88, s
18	1.53, s	1.57, s	1.52, s	1.68, s	1.57, s	1.59, s	1.41, s	1.8, s
20	2.13, s	2.16, s	2.13, s					
1'			3.99, m		4.13, m		4.38, m	
2'			3.91, m		4.06, m		3.88, m	
3''						3.76, d(12.5)		
5''				2.55, s	2.35, s	2.45, s		2.49, s

Table S2: ^{13}C NMR spectroscopic data for compounds **1**, **2**, **5**, **23**, **63**, **74**, **75**, **80**

Position	δC, type						
	**1**[d]	**2**[a]	**5**[a]	**75**[a]	**63**[a]	**80**[b]	**74**[c]
1	152.8, CH	138.4, CH	142.0, CH	142.6, CH	146.5, CH	142.6, CH	141.3, CH
3	158.4, C	146.3, C	144.8, C	149.8, C	157.3, C	149.8, C	148.8, C
4	110.8, CH	110.3, CH	111.8, CH	117.0, CH	106.1, CH	109.7, CH	117.1, CH
4a	138.8, C	147.1, C	148.5, C	150.3, C	140.4, C	145.9, C	154, C
5	106.6, C	101.5, C	102.2, C	98.0, CH	113.6, C	98.8, C	99.1, CH
6	186.0, C	183.7, C	184.4, C	194.4, C	184.5, C	187.1, C	195.5, C
7	84.7, C	85.4, C	84.9, C	85.3, C	83.4, C	83.1, C	86.9, C
8	192.0, C	193.3, C	194.0, C	171.5, C	42.6, CH	197.5, C	172.5, C
8a	114.8, C	114.2, C	114.6, C	117.2, C	110.2, C	115.1, C	117.5, C
9	115.9, CH	116.4, CH	115.1, CH	114.8, CH	120.2, CH	117.2, CH	116.5, CH
10	143.0, CH	142.9, CH	145.1, CH	146.2, CH	145.5, CH	144.3, CH	144.6, CH
11	132.2, C	132.0, C	131.7, C	132.0, C	75.9, C	133.2, C	132.5, C
12	149.0, CH	148.7, CH	148.0, CH	149.0, CH	78.4, CH	146.8, CH	149.7, CH
13	35.3, CH	35.1, CH	35.0, CH	35.1, CH	35.5, CH	34.8, CH	35.4, CH
14	30.2, CH_2	30.6, CH_2	30.0, CH_2	28.6, CH_2	28.7, CH_2	29.8, CH_2	30.3, CH_2
15	12.1, CH_3	12.0, CH_3	12.0, CH_3	12.0, CH_3	12.0, CH_3	11.8, CH_3	12.2, CH_3
16	20.2, CH_3	20.1, CH_3	20.2, CH_3	20.2, CH_3	24.0, CH_3	20.1, CH_3	20.5, CH_3
17	12.5, CH_3	12.4, CH_3	12.6, CH_3	12.6, CH_3	13.5, CH_3	12.3, CH_3	12.4, CH_3
18	22.7, CH_3	23.6, CH_3	23.3, CH_3	30.2, CH_3	23.4, CH_3	28.2, CH_3	29.9, CH_3
19	170.3, C	170.9, C	170.2, C				
20	20.3, CH_3	20.6, CH_3	20.3, CH_3				
1'			55.4, CH_2	57.1, CH_2		56.6, CH_2	
2'			60.9, CH_2	60.4, CH_2		60.5, CH_2	
2''				172.3, C	168.1, C		175, C
3''				103.6, C	57.3, CH		101.2, C
4''				193.9, C	199.7, C		193.5, C
5''				30.0, CH_3	30.3, CH_3		28.6, CH_3

[a] Recorded at 500 MHz in $CDCl_3$
[b] Recorded at 700 MHz in DMF-*d7*
[b] Recorded at 300 MHz in $CDCl_3$
[d] Recorded at 600 MHz in $CDCl_3$

Figure S83: ^{1}H NMR spectrum ($CDCl_3$) of compound **1**

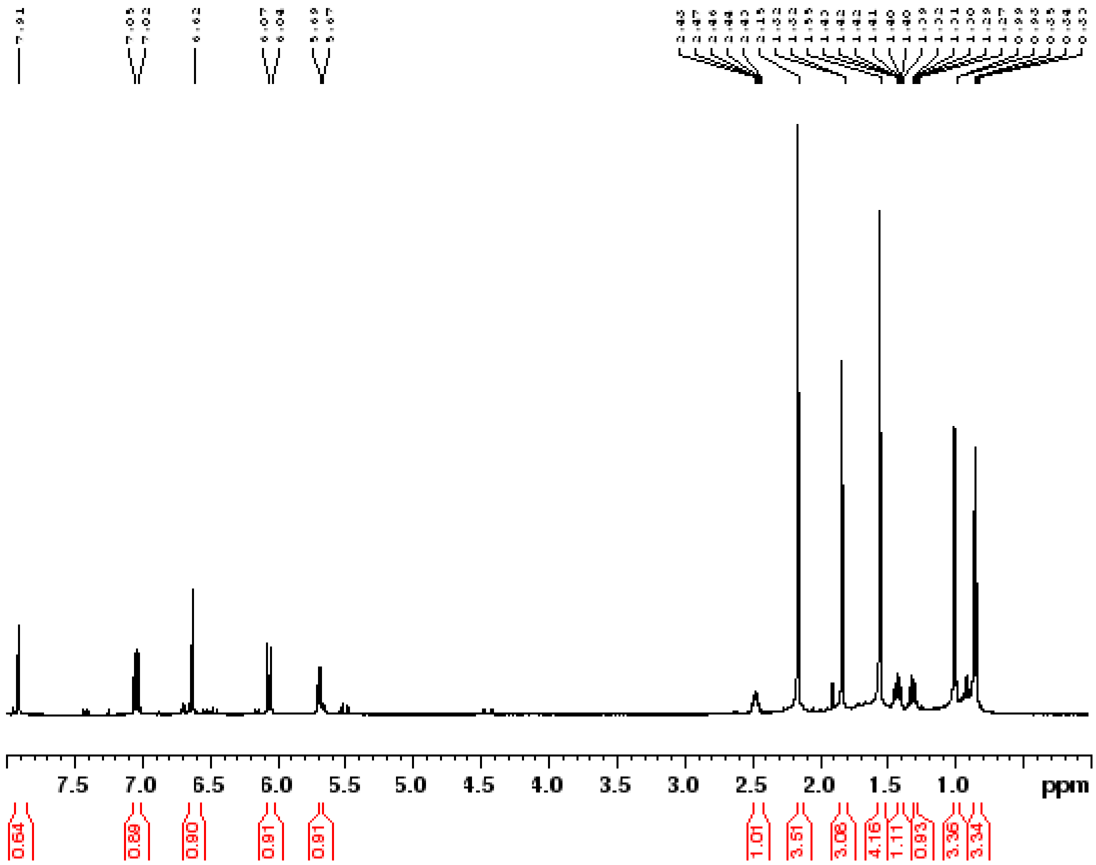

Figure S84: 13C NMR spectrum ($CDCl_3$) of compound **1**

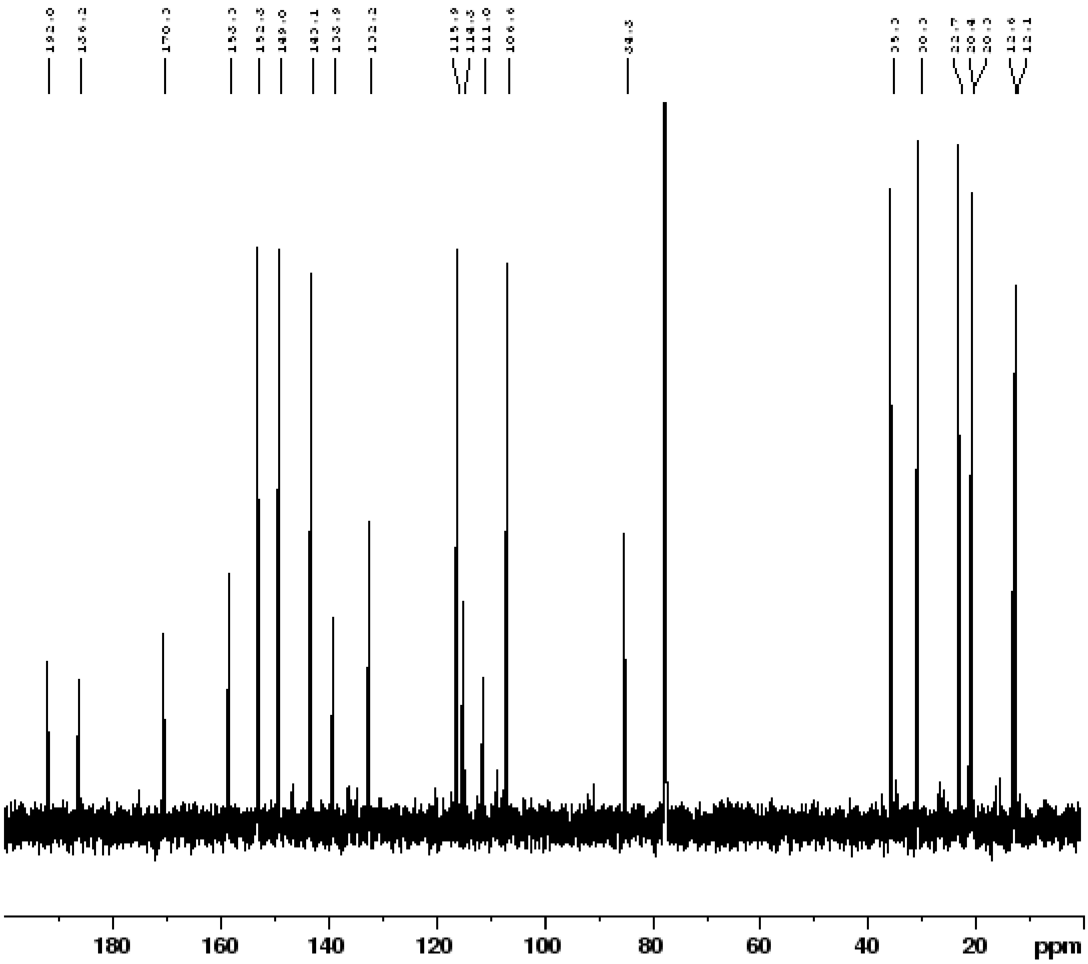

Figure S85: HRMS of compound **1**

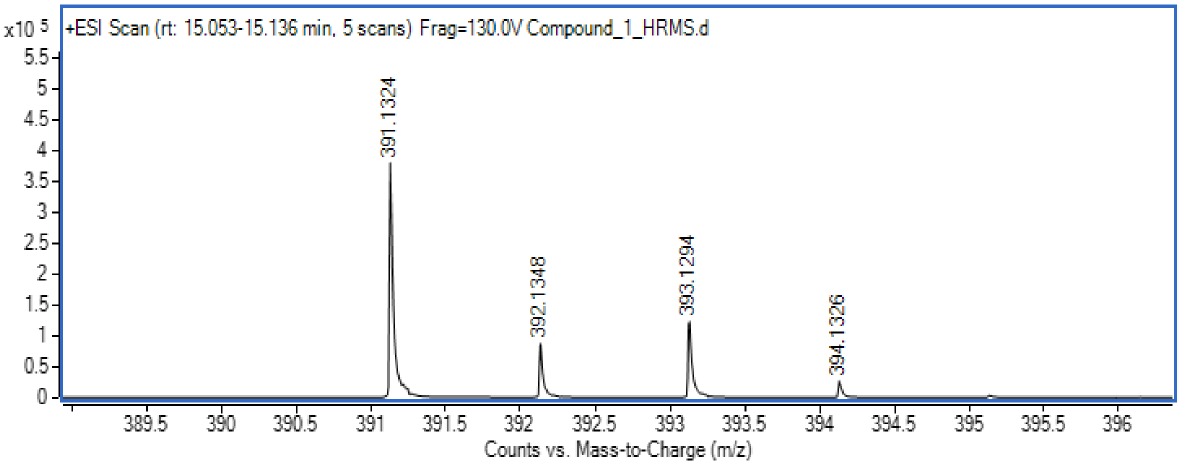

Figure S86: ^{1}H NMR spectrum ($CDCl_3$) of compound **2**

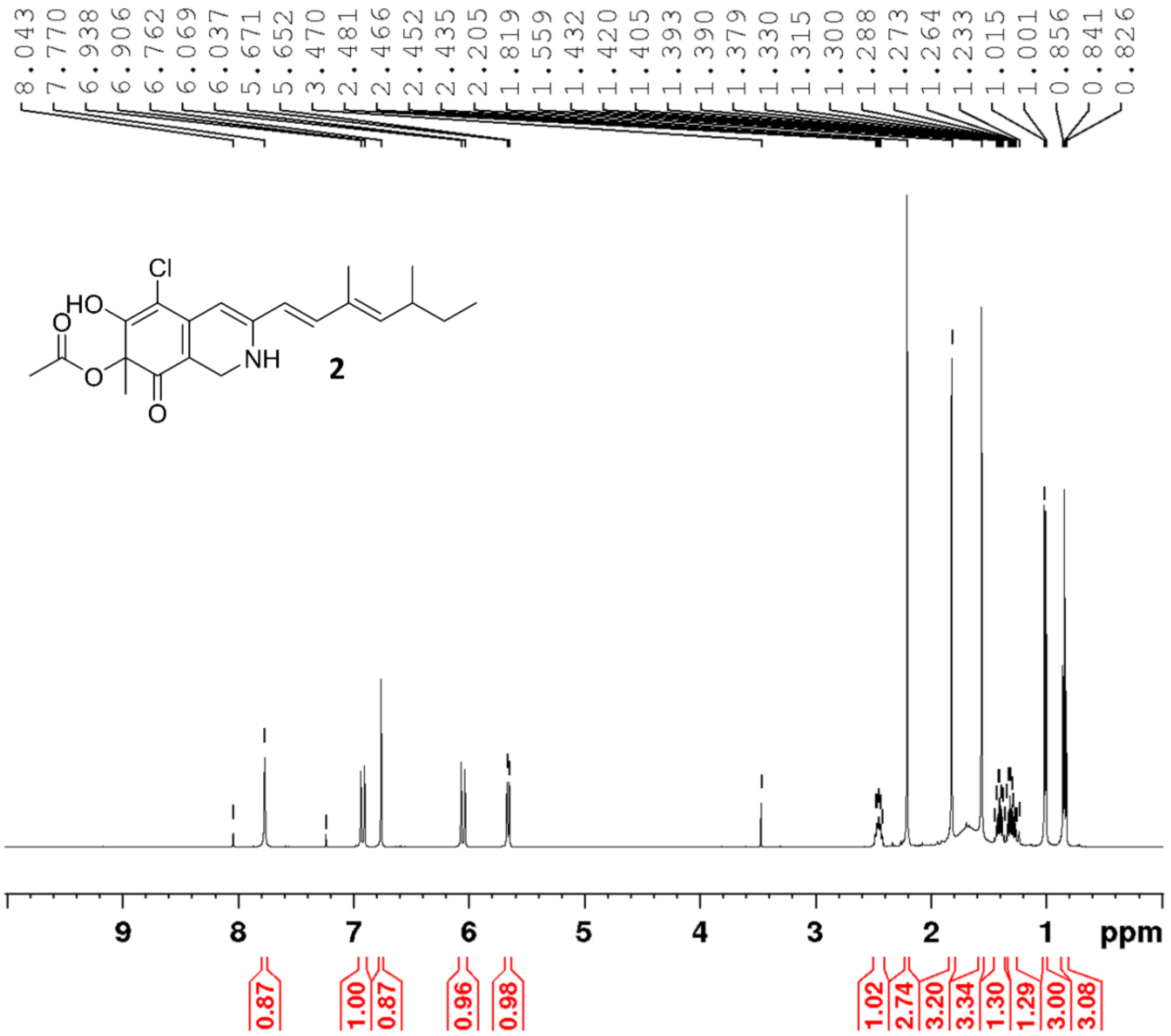

Figure 87: ^{13}C NMR spectrum ($CDCl_3$) of compound **2**

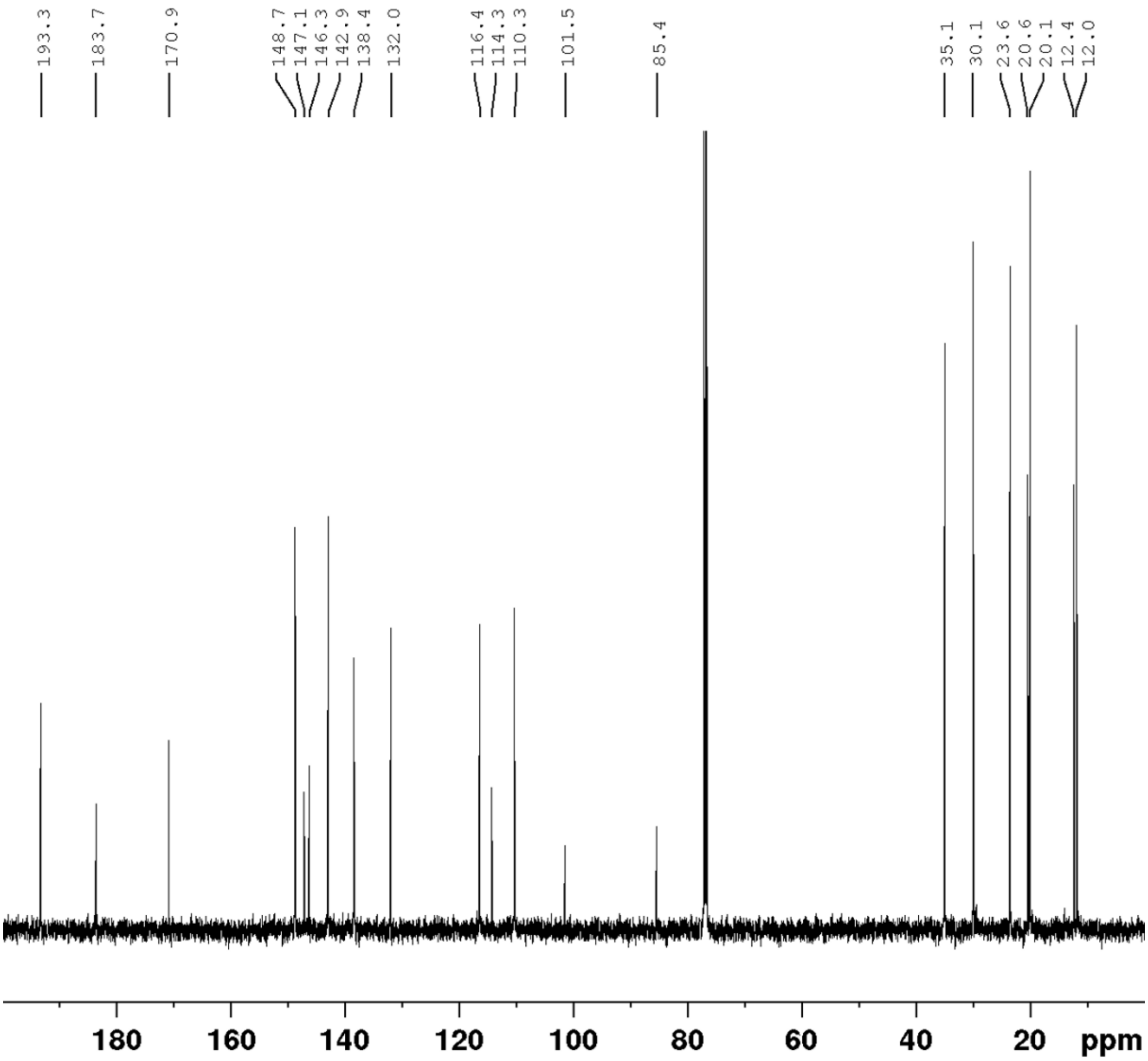

Figure 88: HRMS of compound **2**

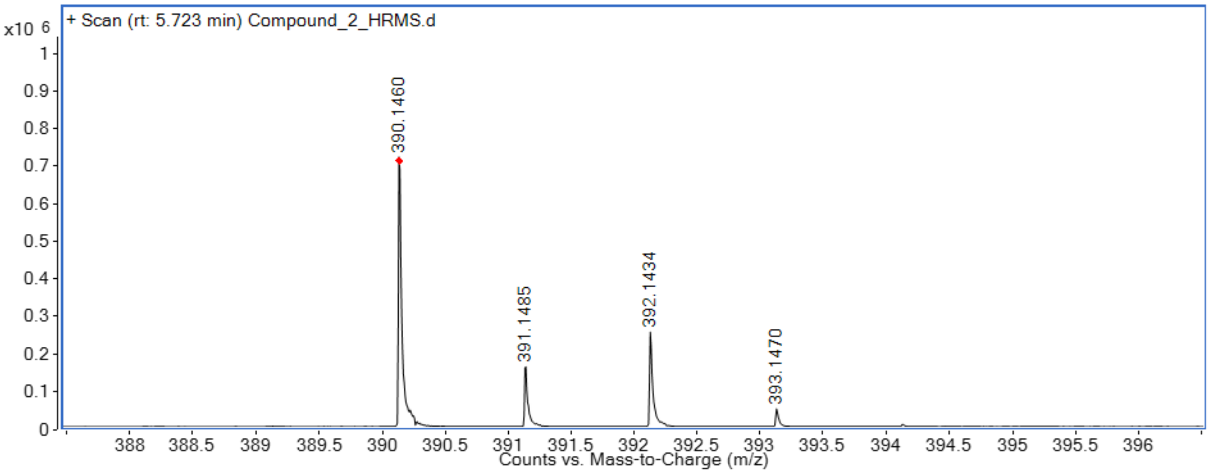

Figure S89: ^{1}H NMR spectrum ($CDCl_3$) of compound **5**

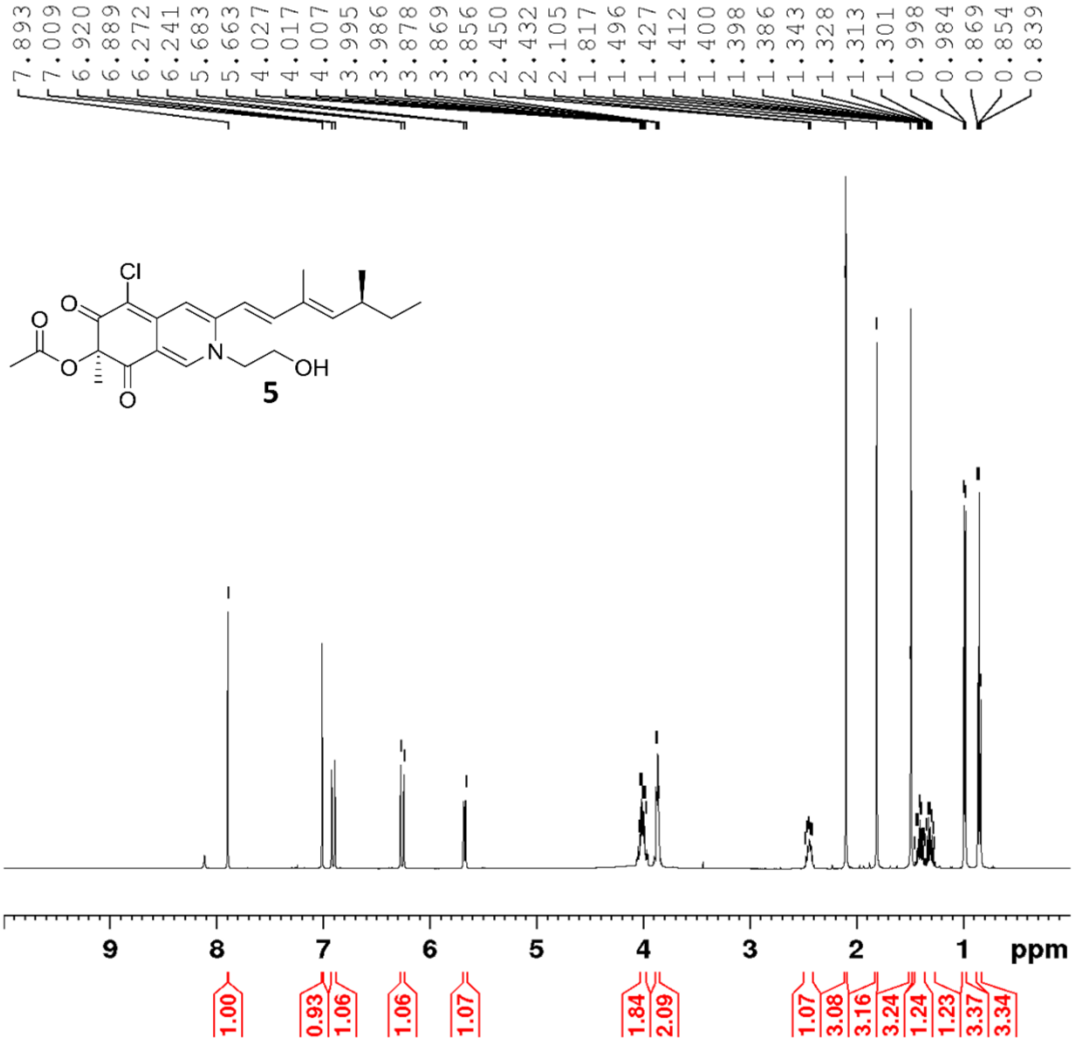

Figure S90: ^{13}C NMR spectrum ($CDCl_3$) of compound **5**

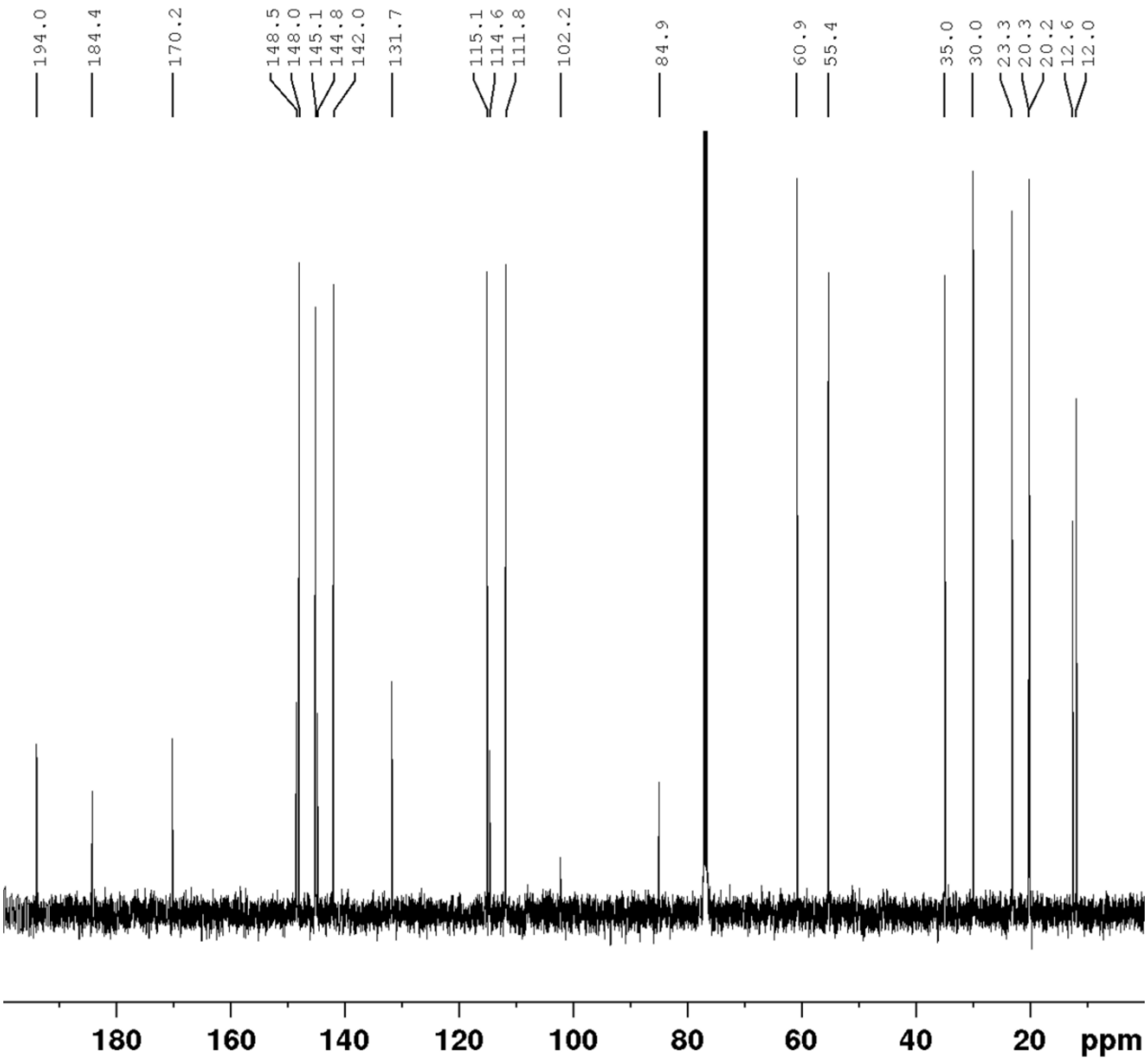

Figure S91: HRMS of compound **5**

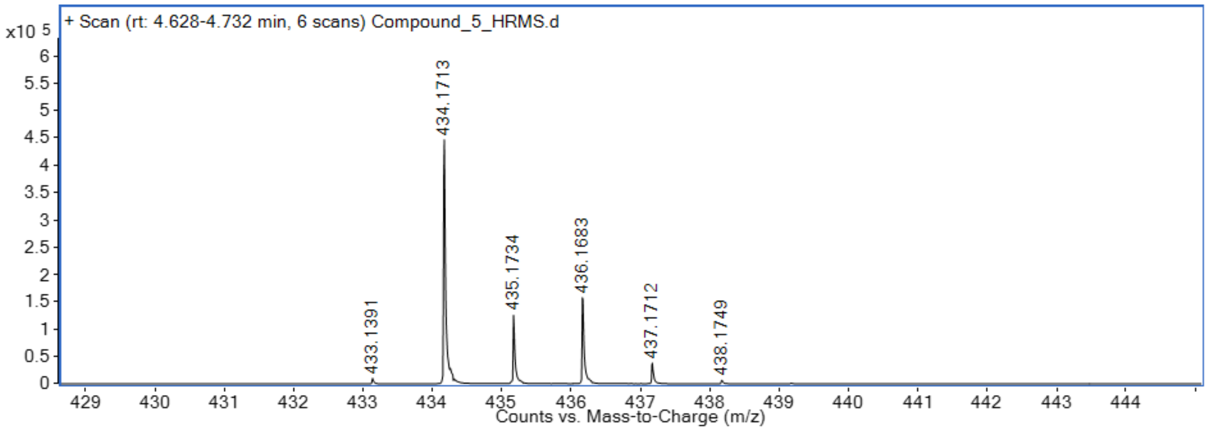

Figure S92: ^{1}H NMR (MeOD) spectrum of compound compound **23**

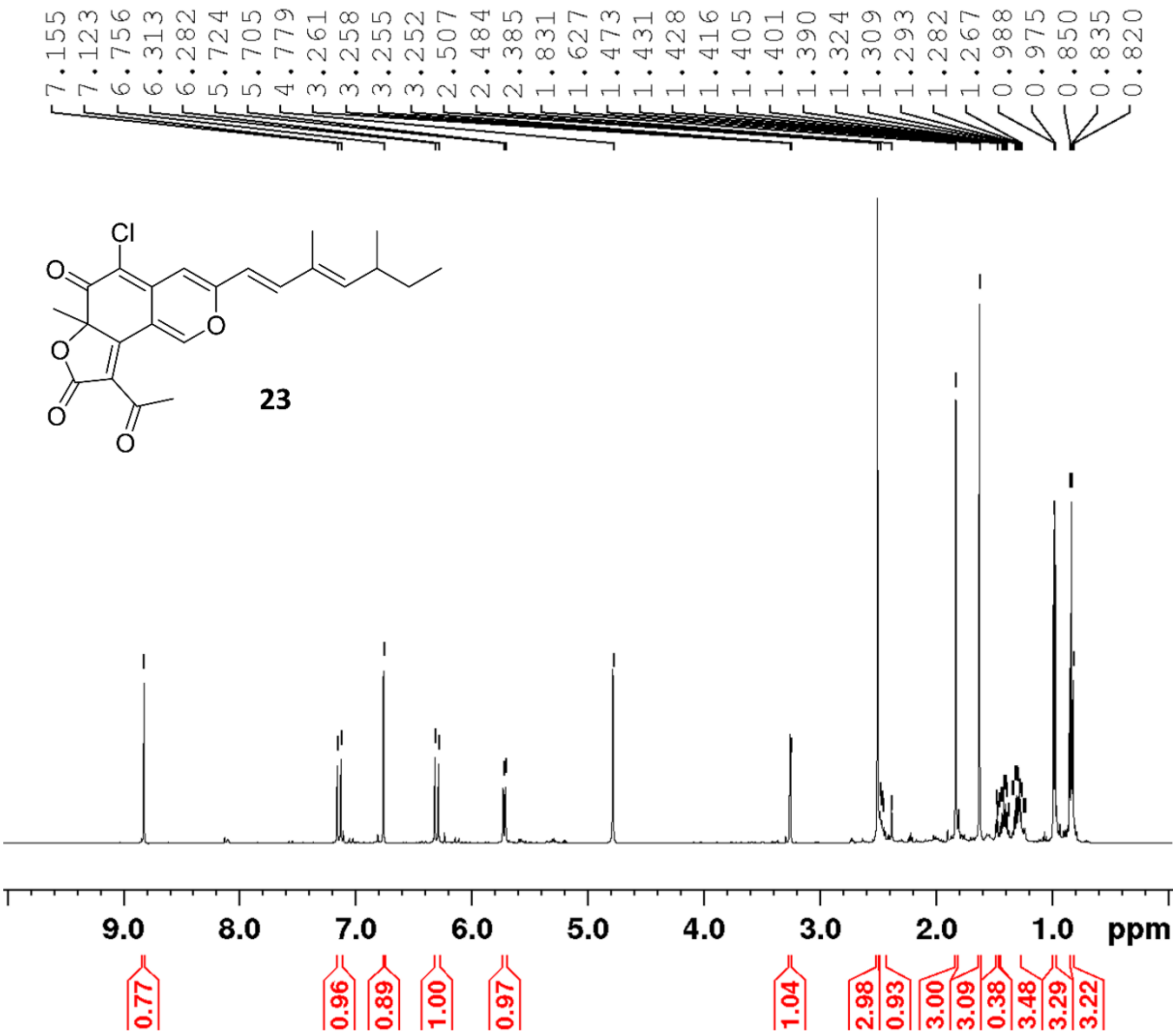

Figure S93: HRMS of compound **23**

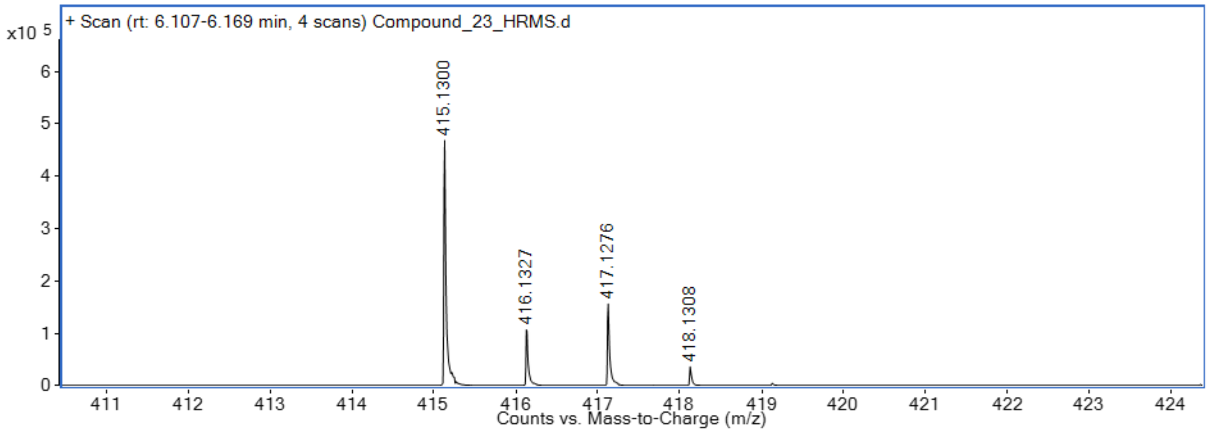

Figure S94: ^{1}H NMR spectrum ($CDCl_3$) of compound **75**

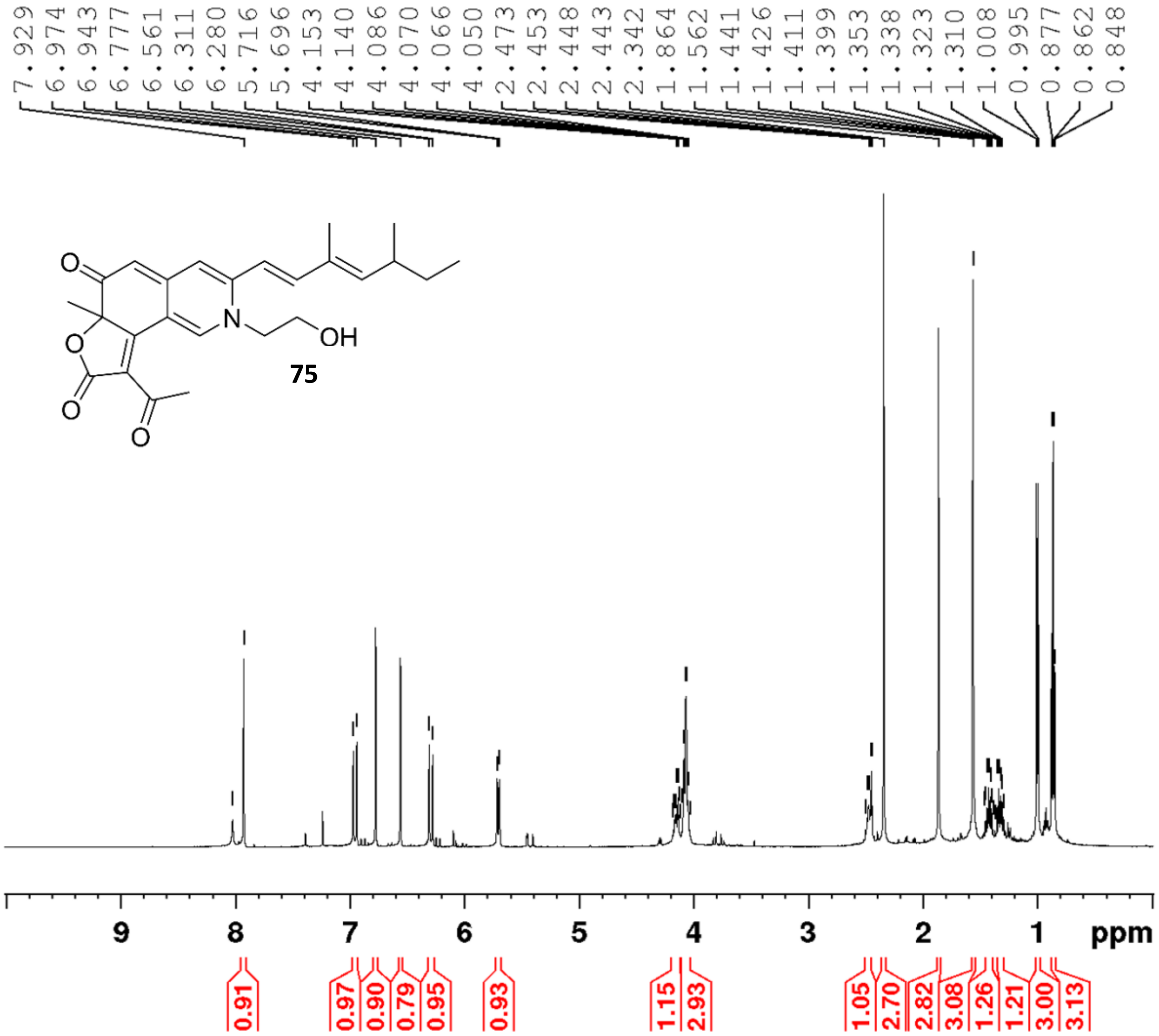

Figure S95: ^{13}C NMR ($CDCl_3$) spectrum of compound **75**

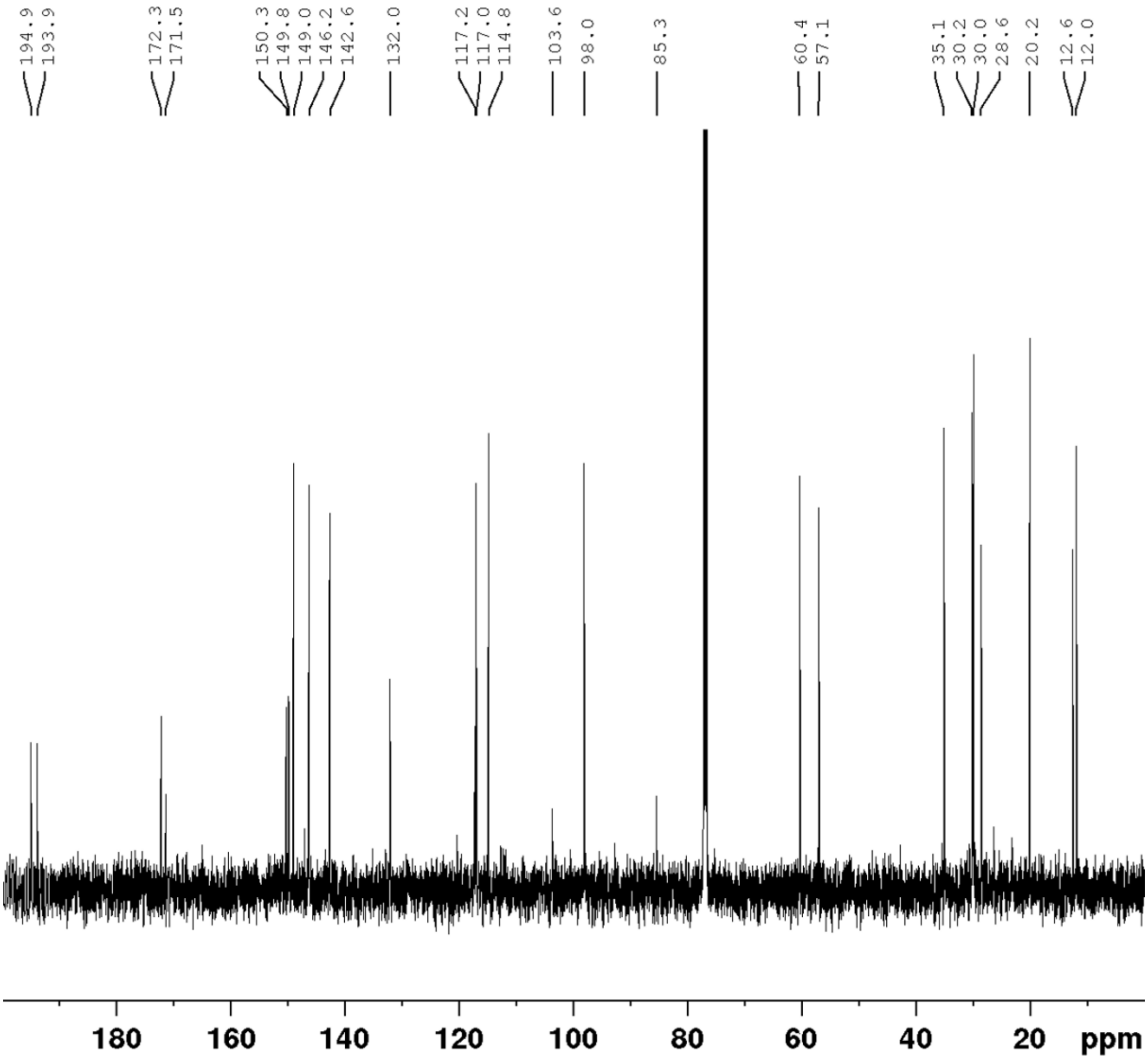

Figure S96: HRMS of compound **75**

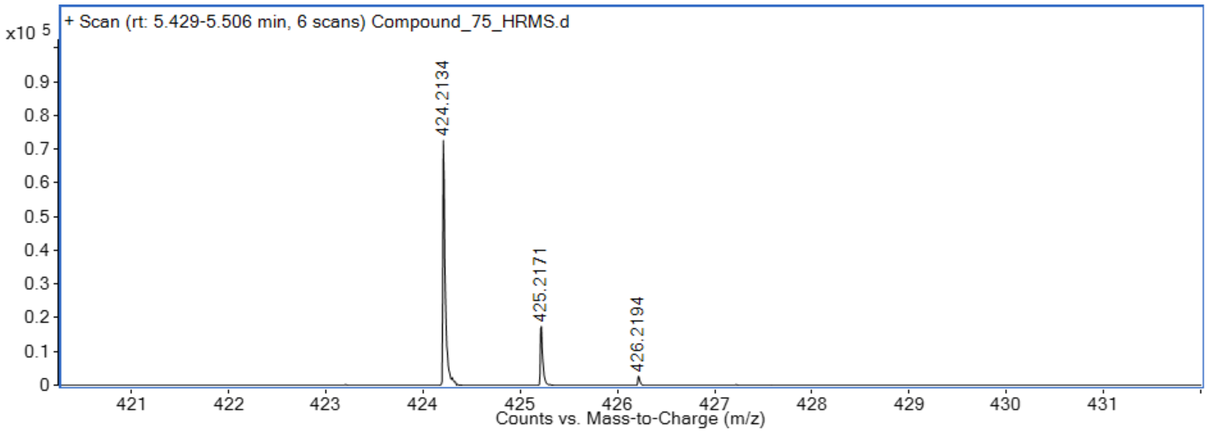

Figure S97: ^{1}H NMR spectrum ($CDCl_3$) of compound **63**

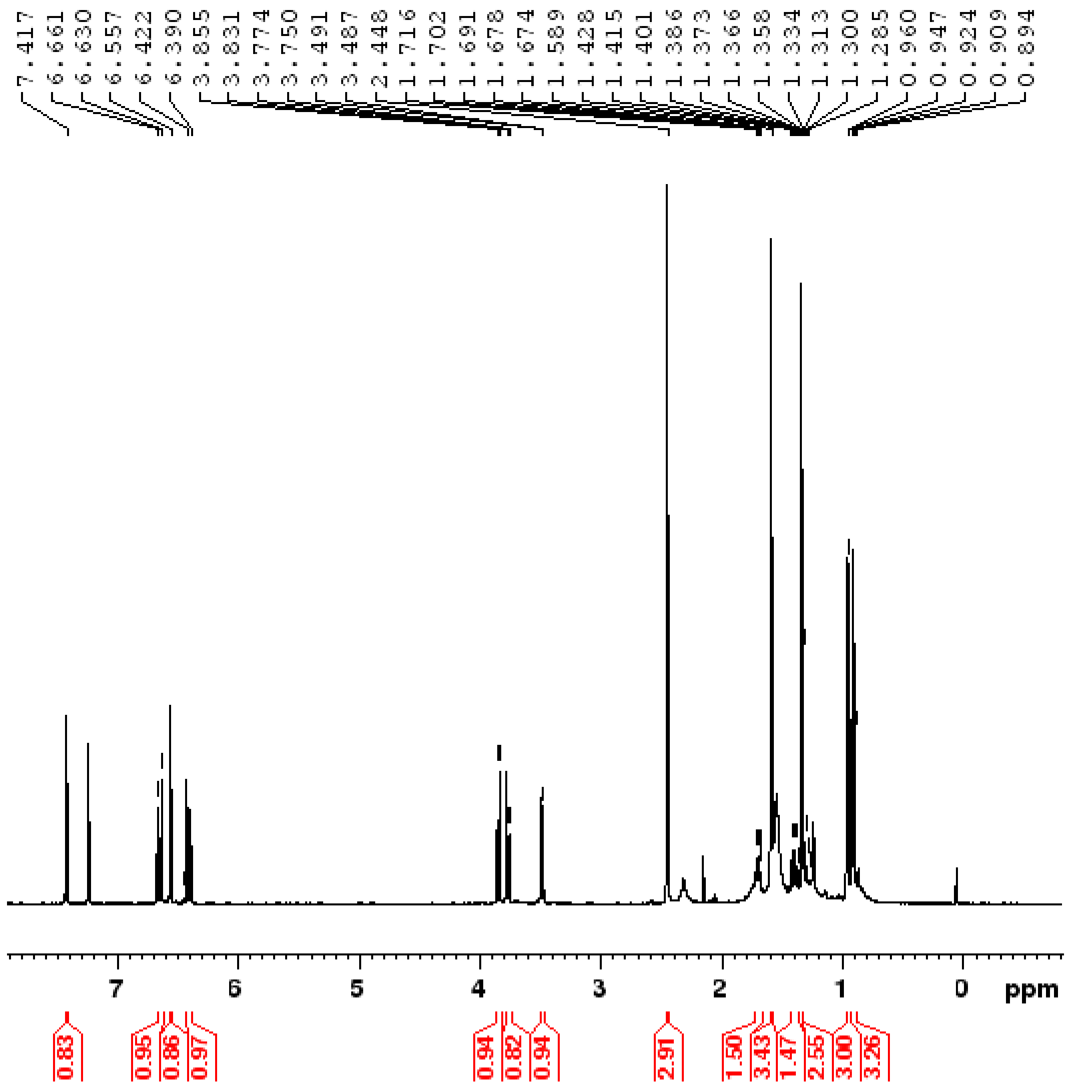

Figure S98: ^{13}C NMR spectrum ($CDCl_3$) of compound **63**

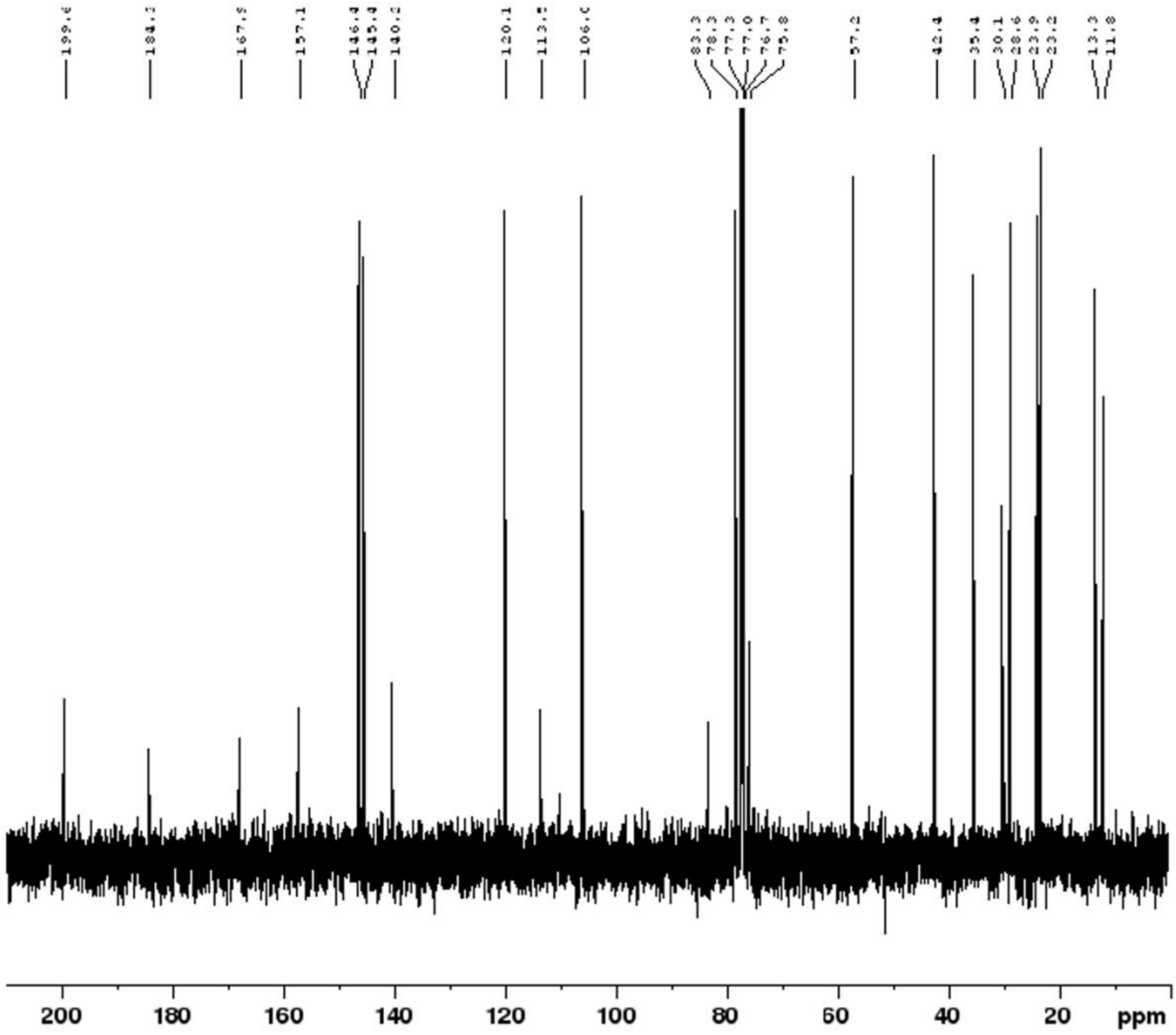

Figure S99: COSY NMR spectrum ($CDCl_3$) of compound **63**

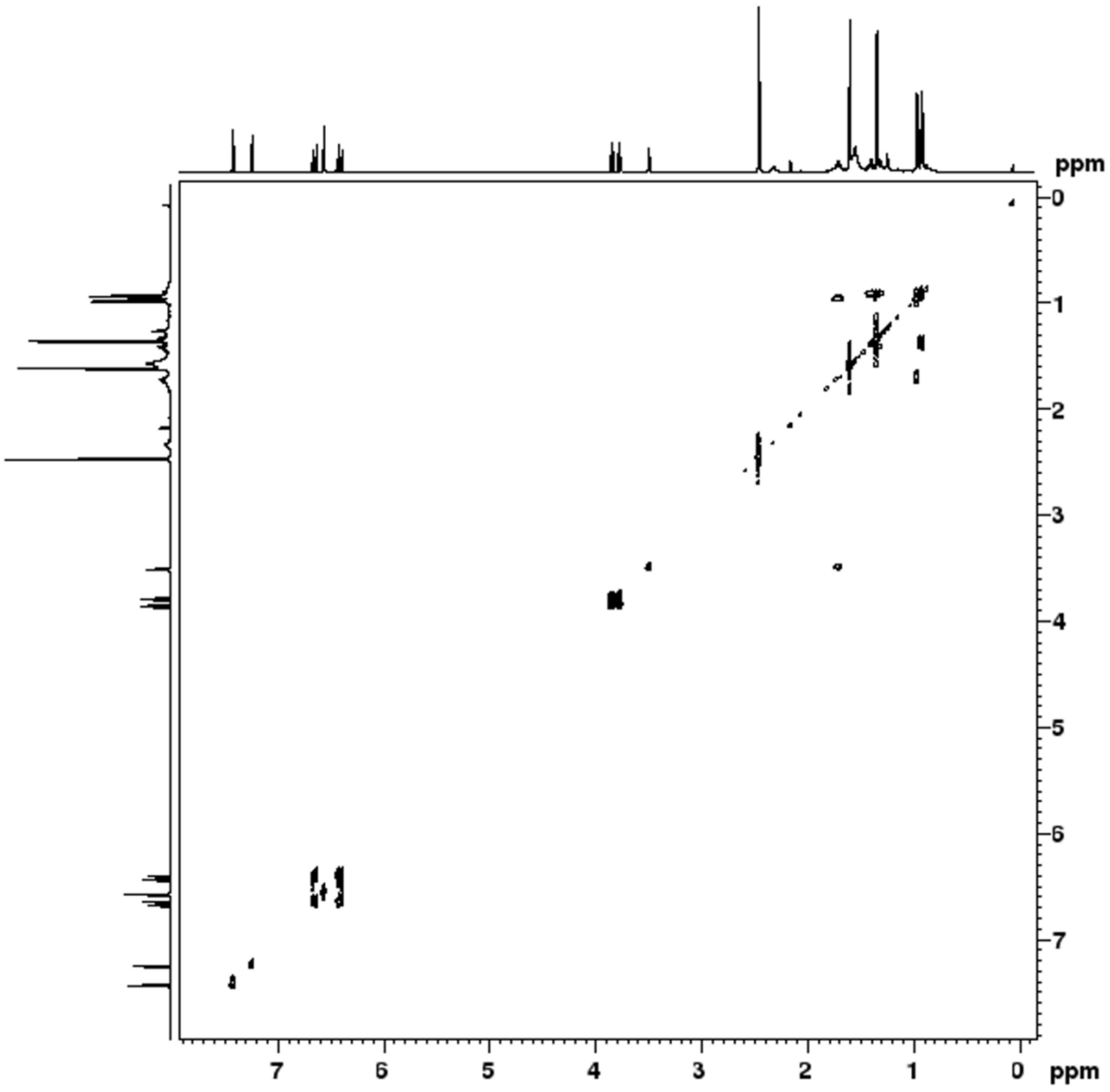

Figure S100: HSQC NMR spectrum ($CDCl_3$) of compound **63**

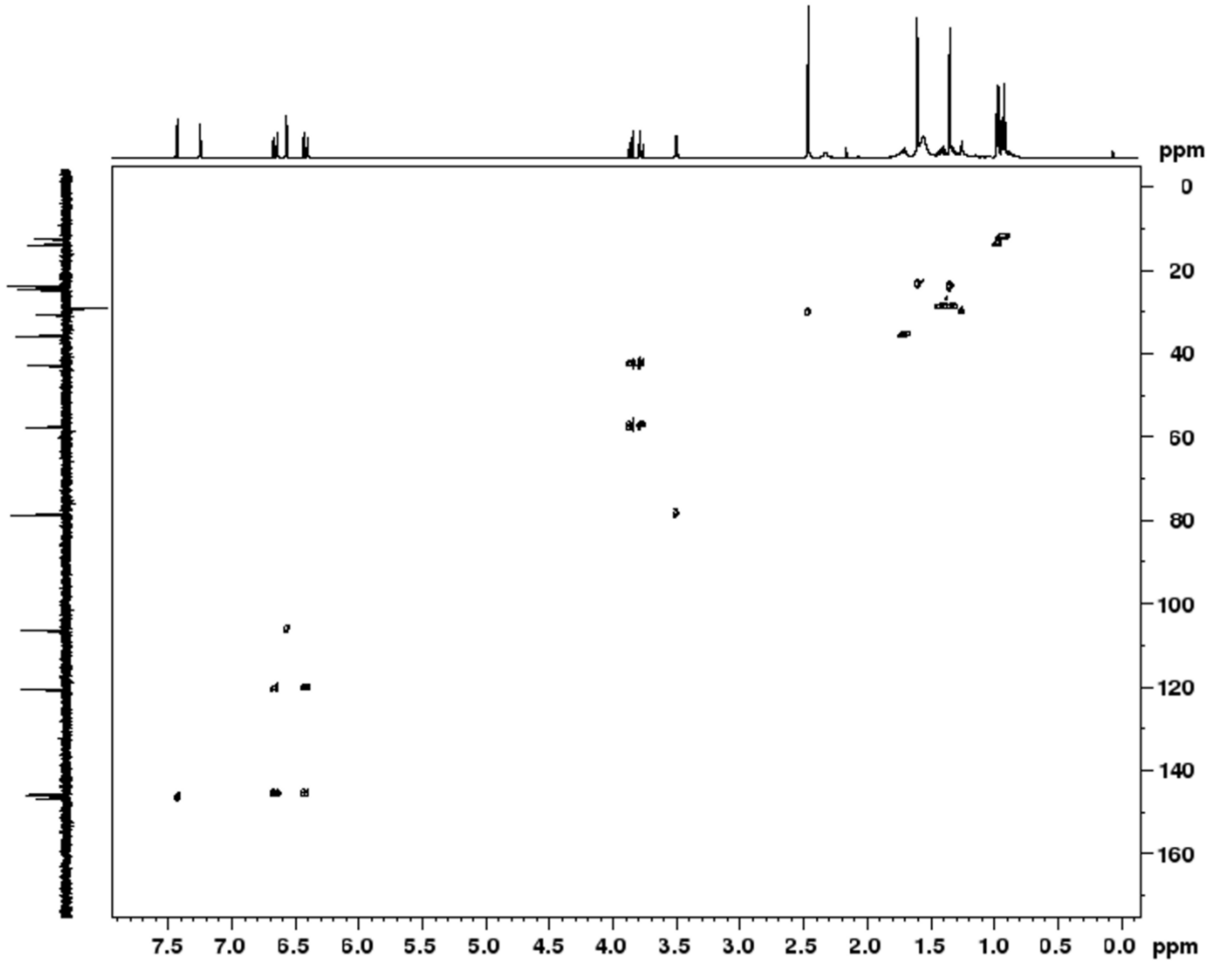

Figure S101: HMBC NMR spectrum ($CDCl_3$) of compound **63**

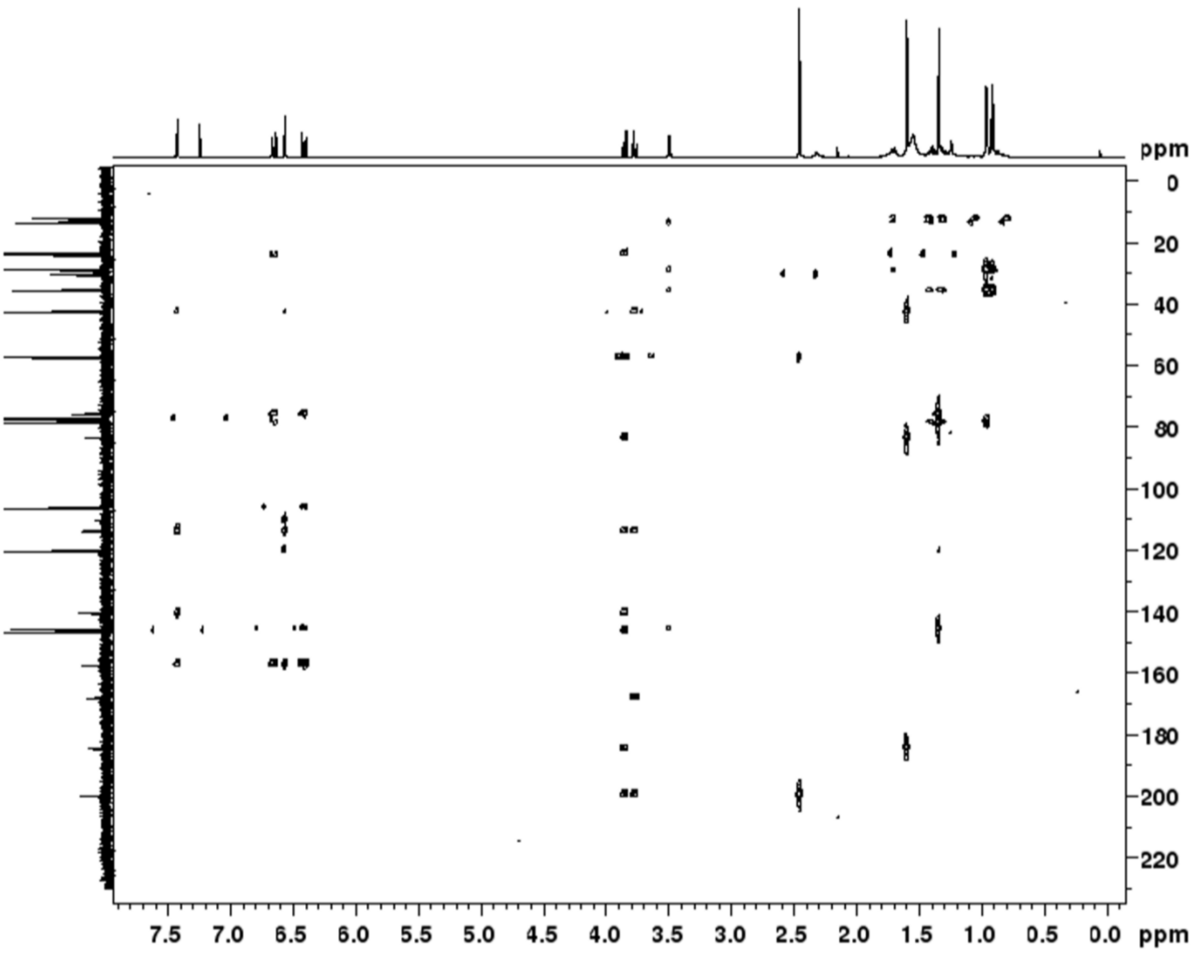

Figure S102: HRMS of compound **63**

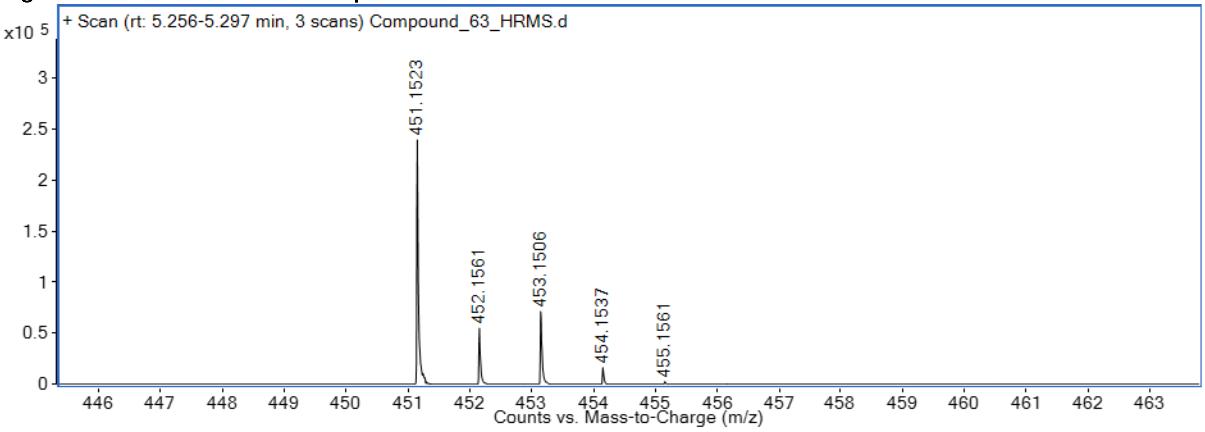

Figure S103: ^{1}H NMR spectrum ($CDCl_3$) of compound **74**

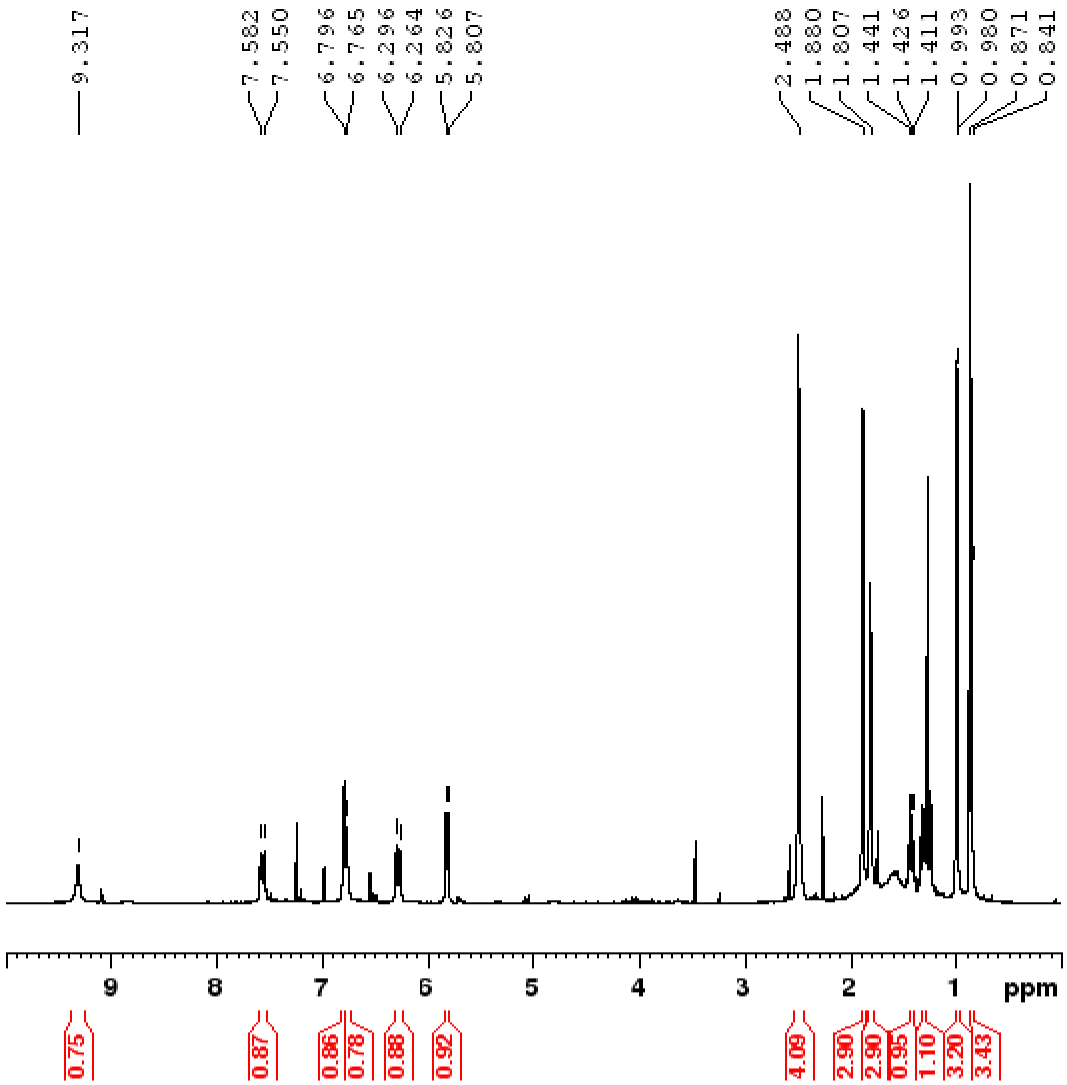

Figure S104: ^{13}C NMR spectrum ($CDCl_3$) of compound **74**

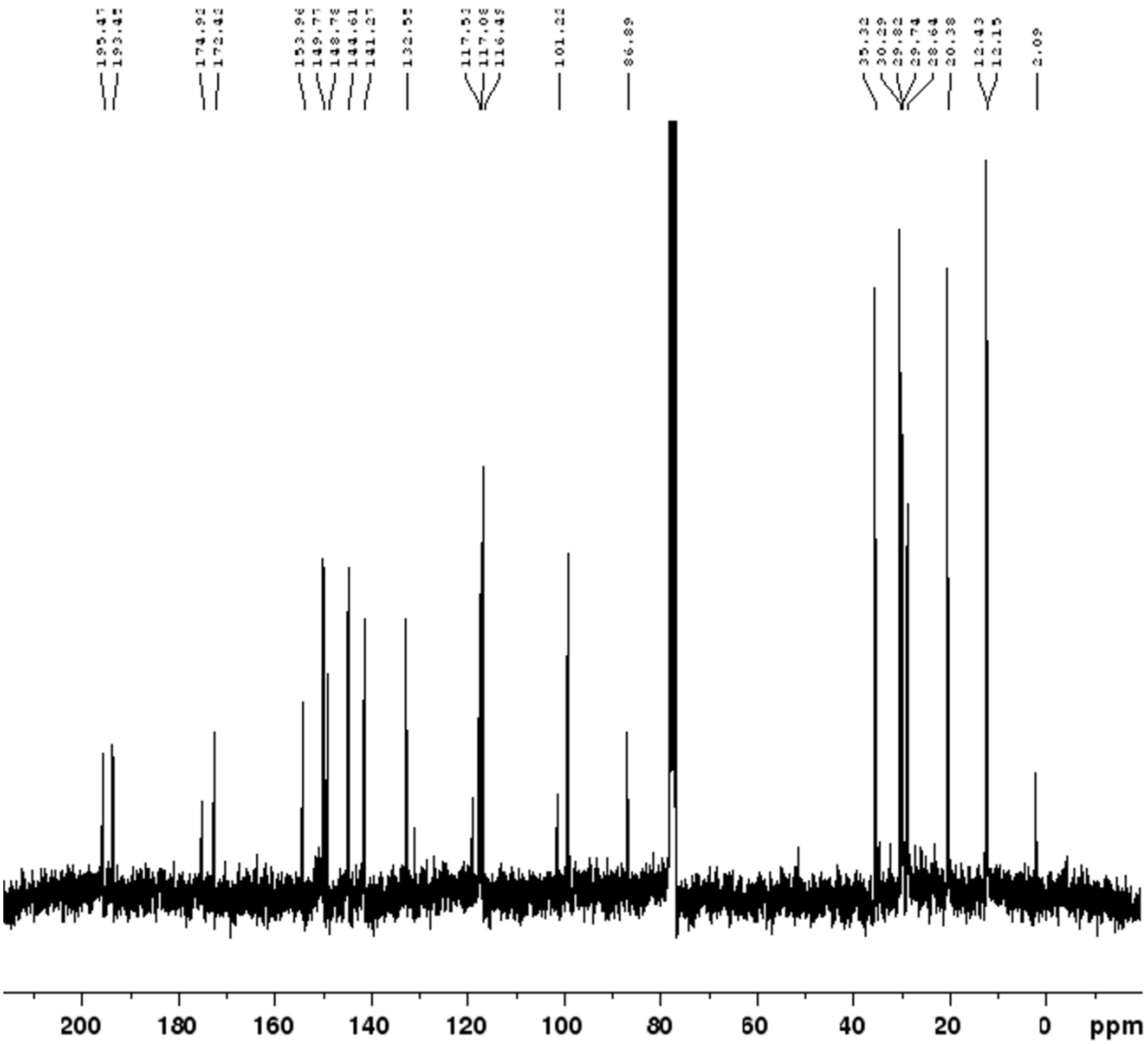

Figure S105: COSY NMR spectrum ($CDCl_3$) of compound **74**

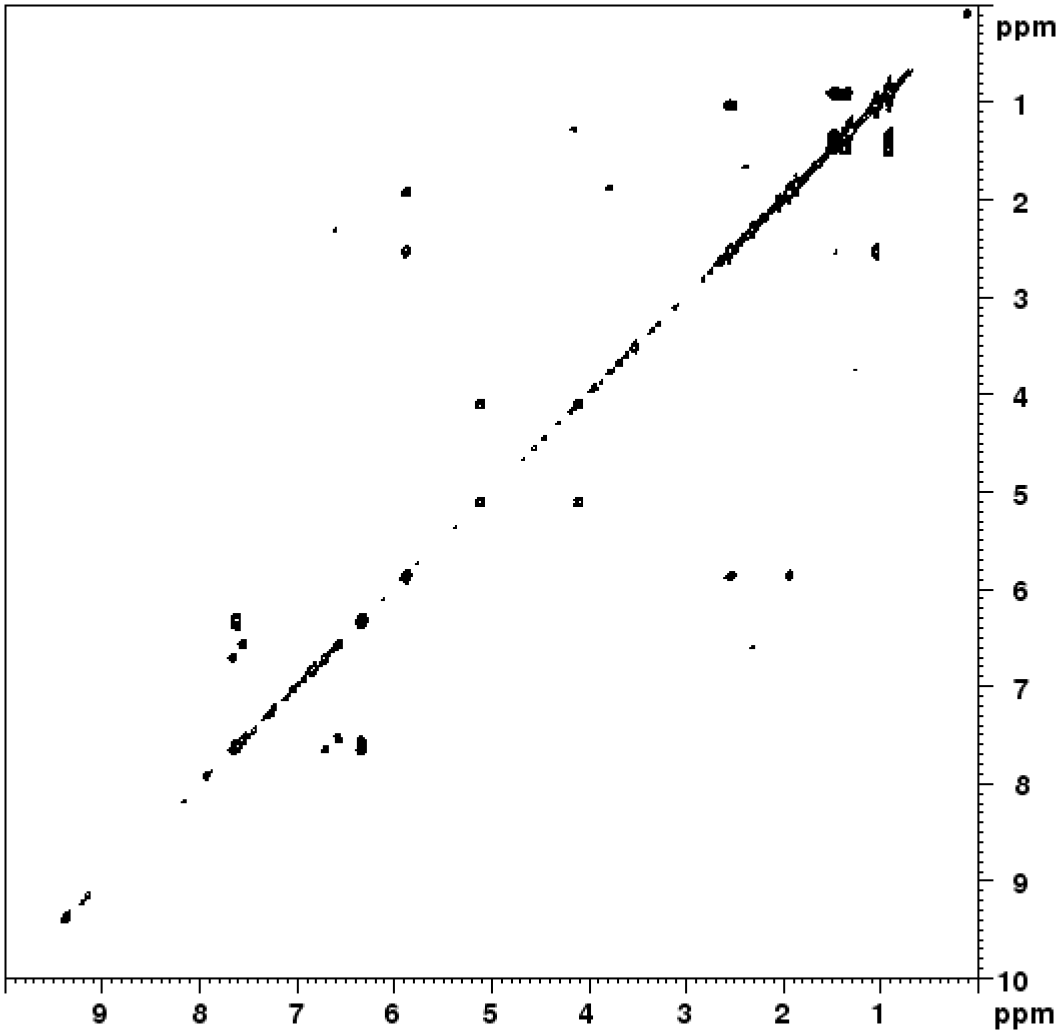

Figure S106: HSQC NMR spectrum ($CDCl_3$) of compound **74**

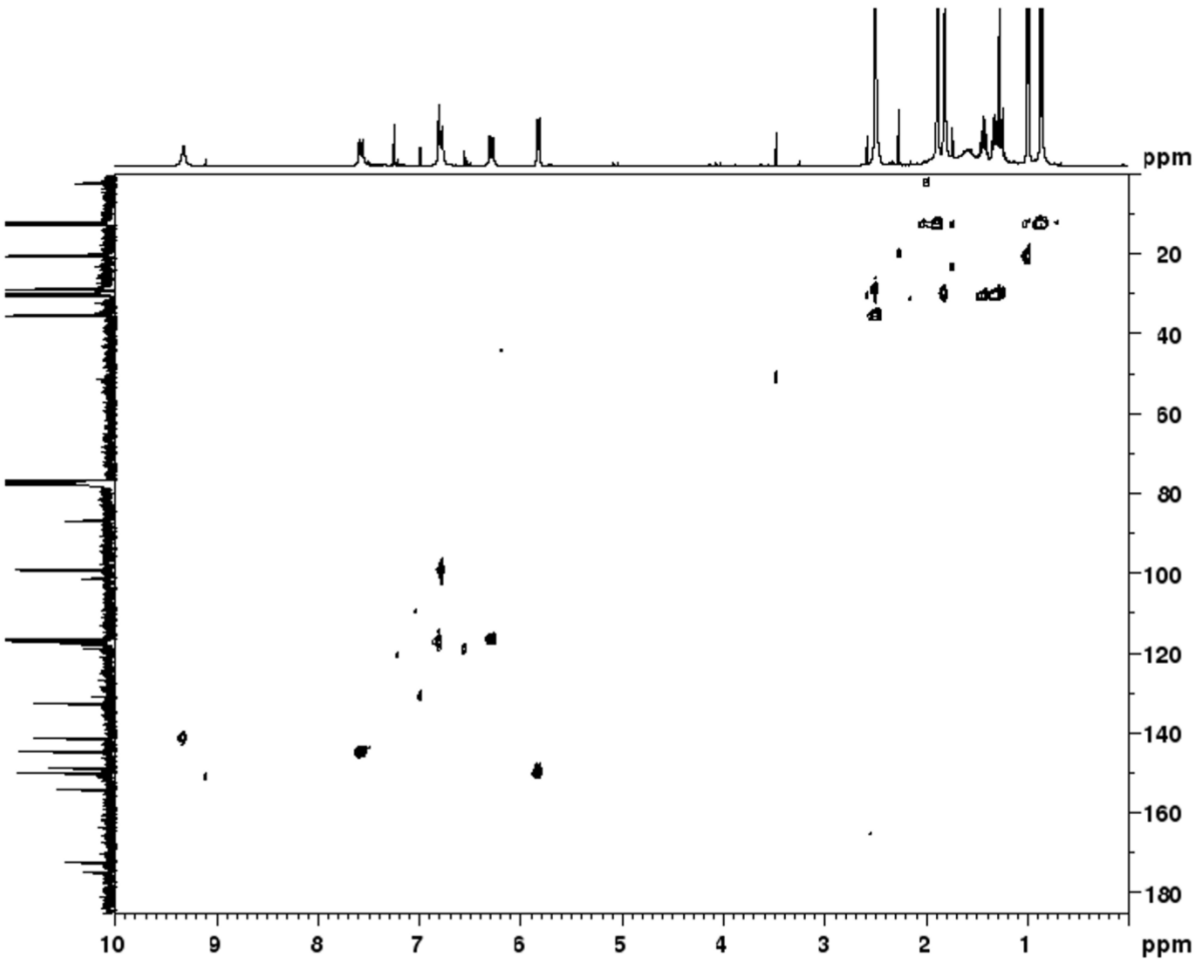

Figure S107: HMBC NMR spectrum ($CDCl_3$) of compound **74**

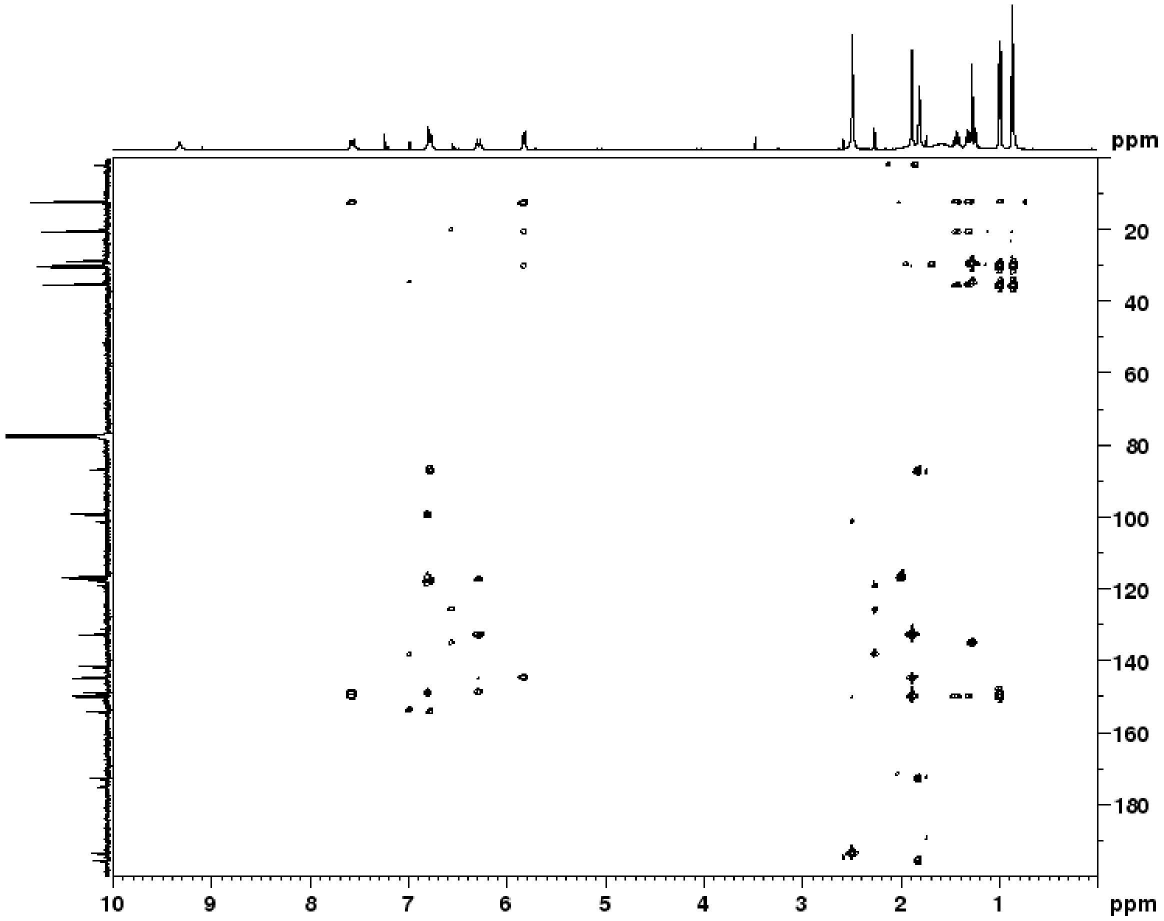

Figure S108: HRMS of compound **74**

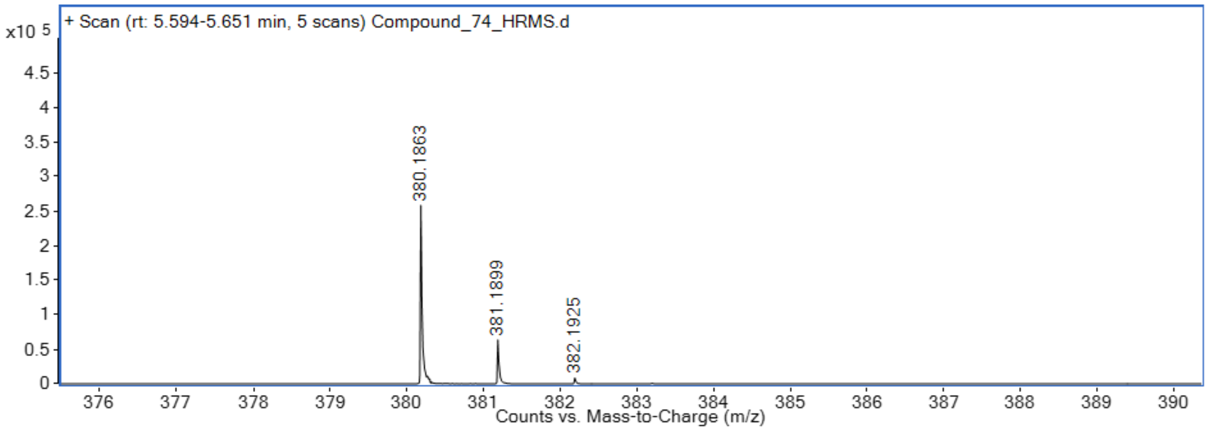

Figure S109: Compound **80** position within t-SNE molecular network

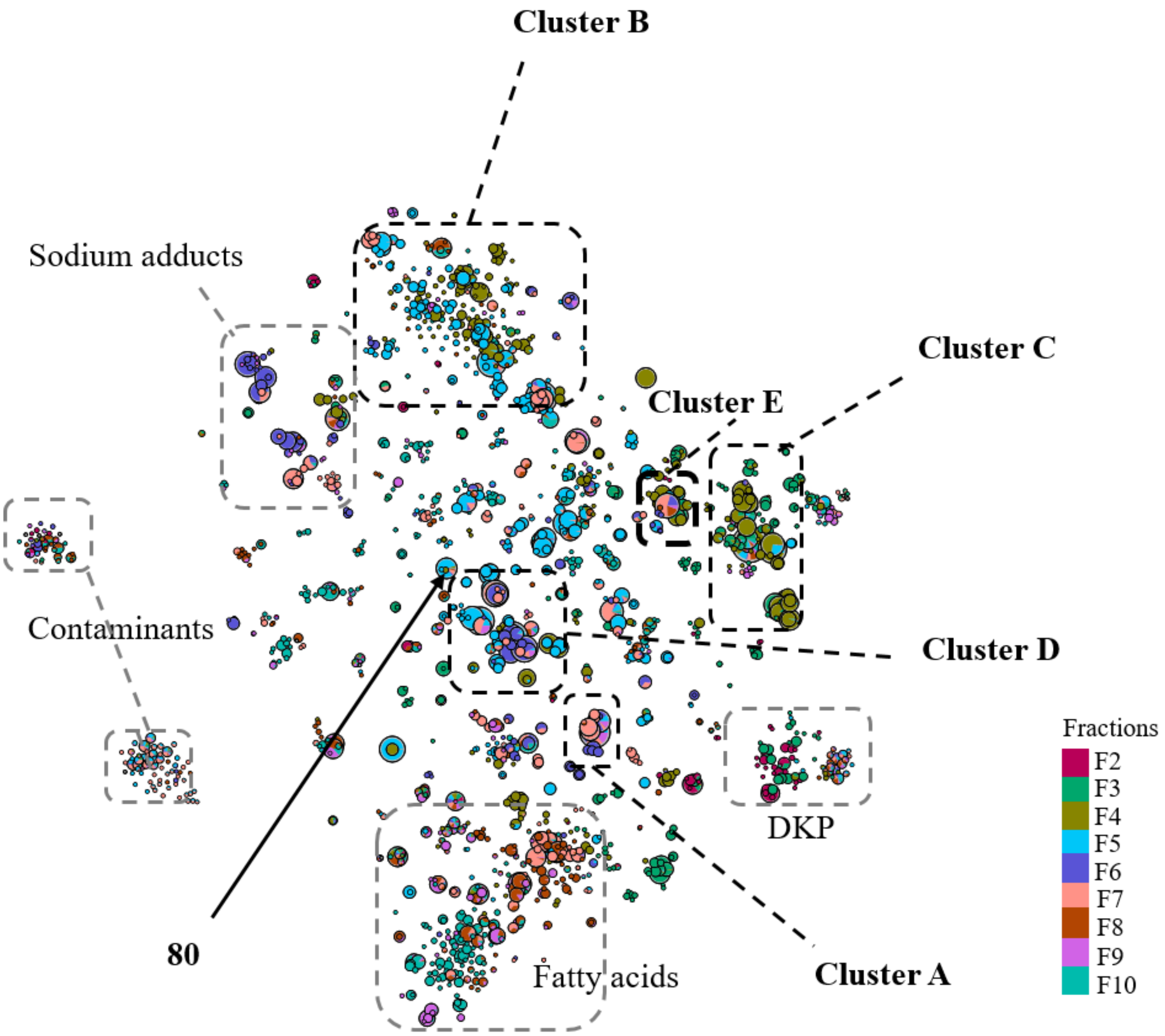

Figure S110: MS/MS information of compound **80** (*m/z* 392.1628, 1.3 ppm, level 0) from molecular network. (a) MS/MS spectrum of compound **80**, (**b**) the mirror plot of MS/MS spectra from compound **80**) against **5** with common neutral loss from CO and OH (**c**) common fragment and neutral loss with their contribution to cosine score.

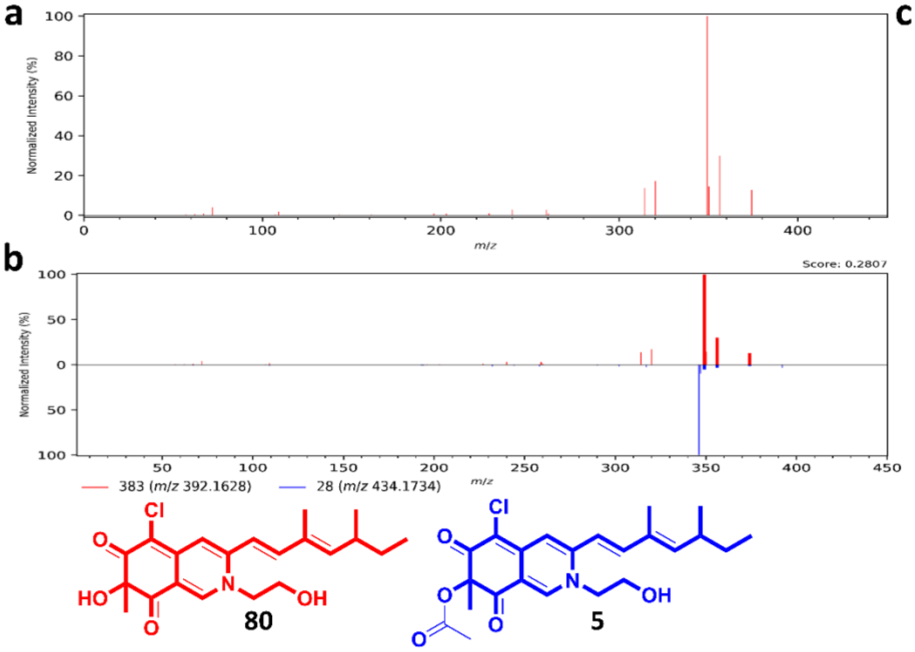

c

Fragment	Score
57.0693	0.0012
67.0543	0.005
72.045	0.0053
107.0858	0.0015
109.1013	0.0097
349.1451	0.1333
356.1864	0.0596
374.1522	0.0231

Neutral Loss	Score
42.0129	0.0421

Figure S111: ^{1}H NMR spectrum (DMF-*d6*) of compound **80**

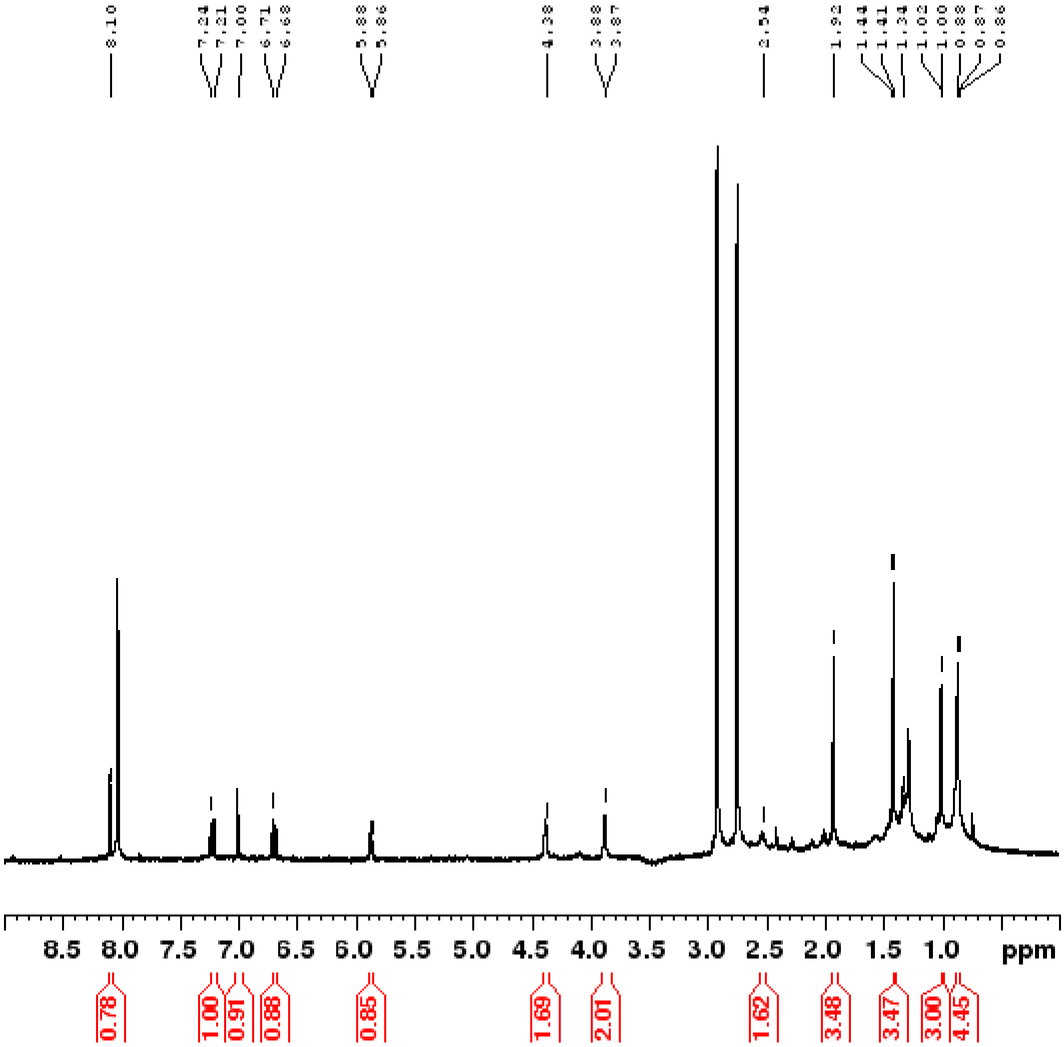

Figure S112: ^{13}C NMR spectrum (DMF-*d6*) of compound **80**

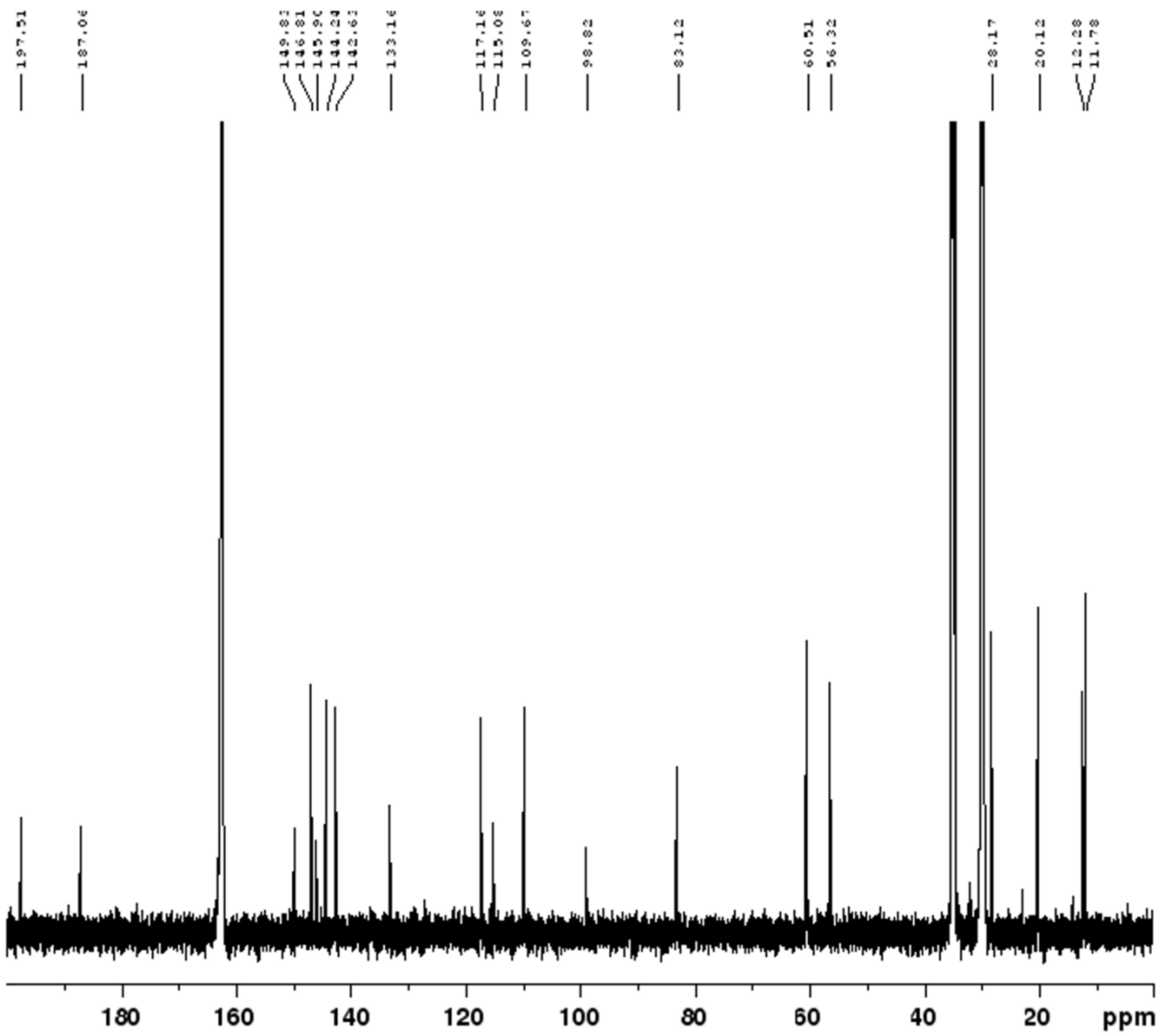

Figure S113: COSY NMR spectrum (DMF-*d6*) of compound **80**

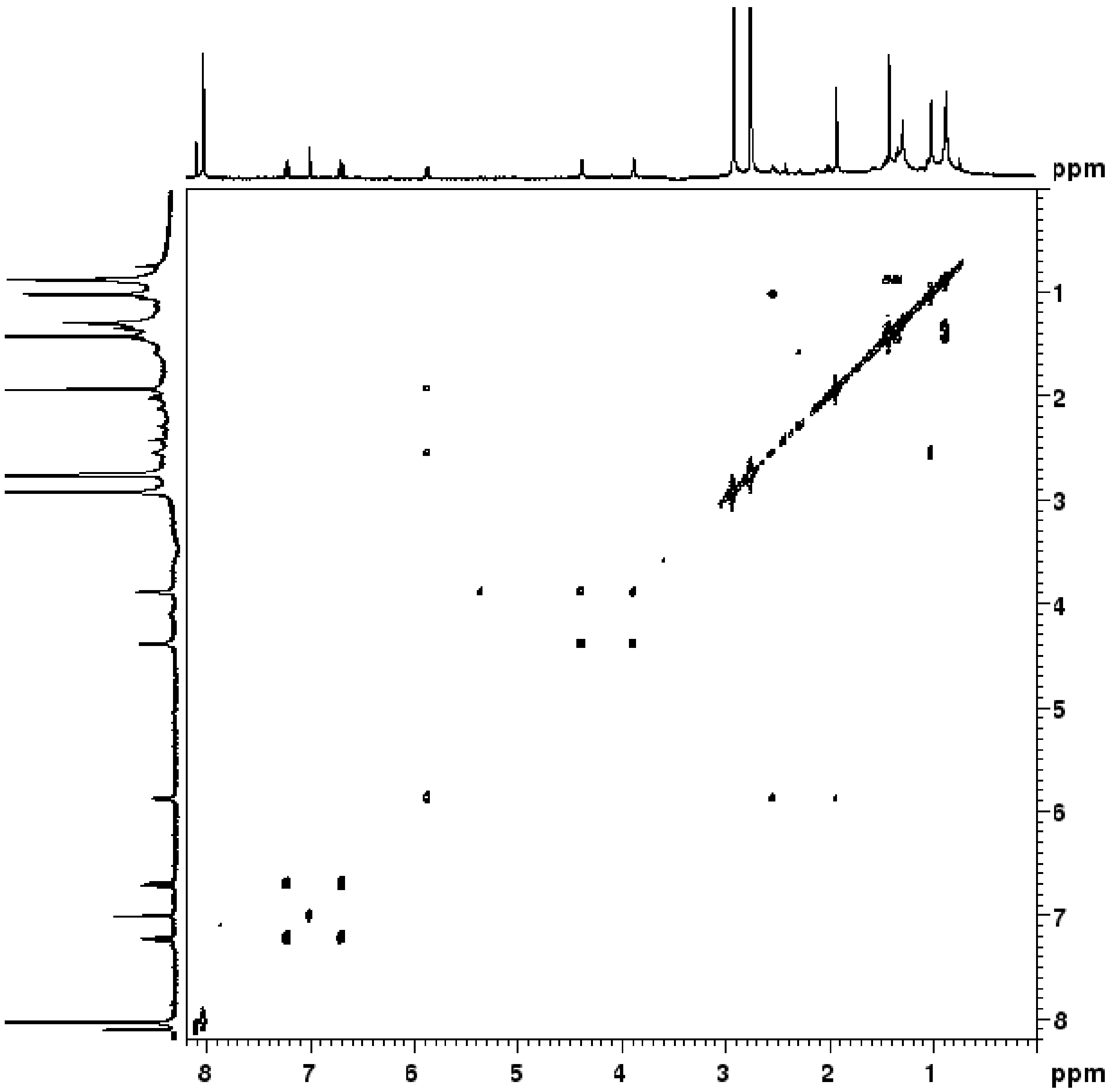

Figure S114: HSQC NMR spectrum (DMF-*d6*) of compound **80**

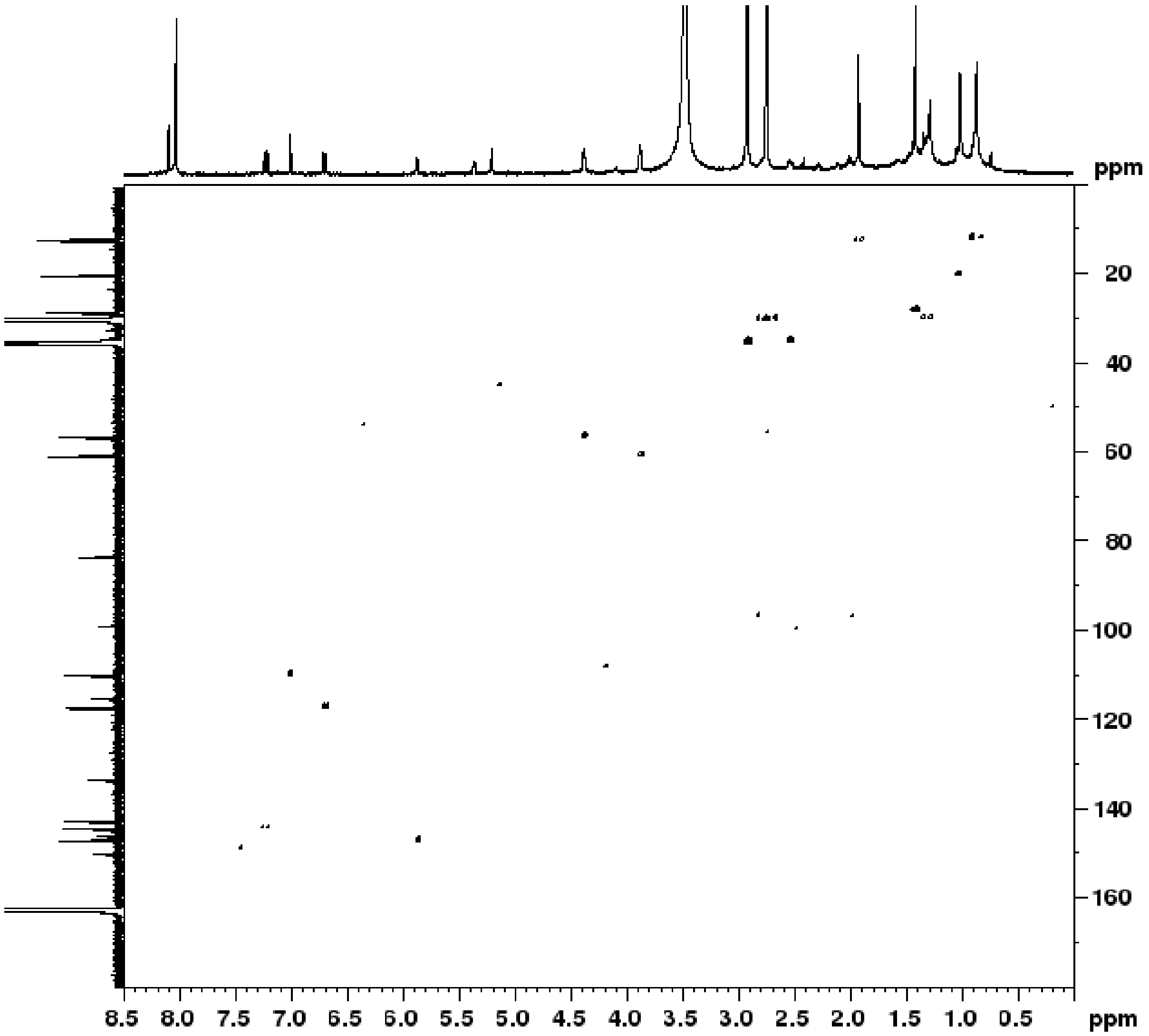

Figure S115: HMBC NMR spectrum (DMF-d6) of compound **80**

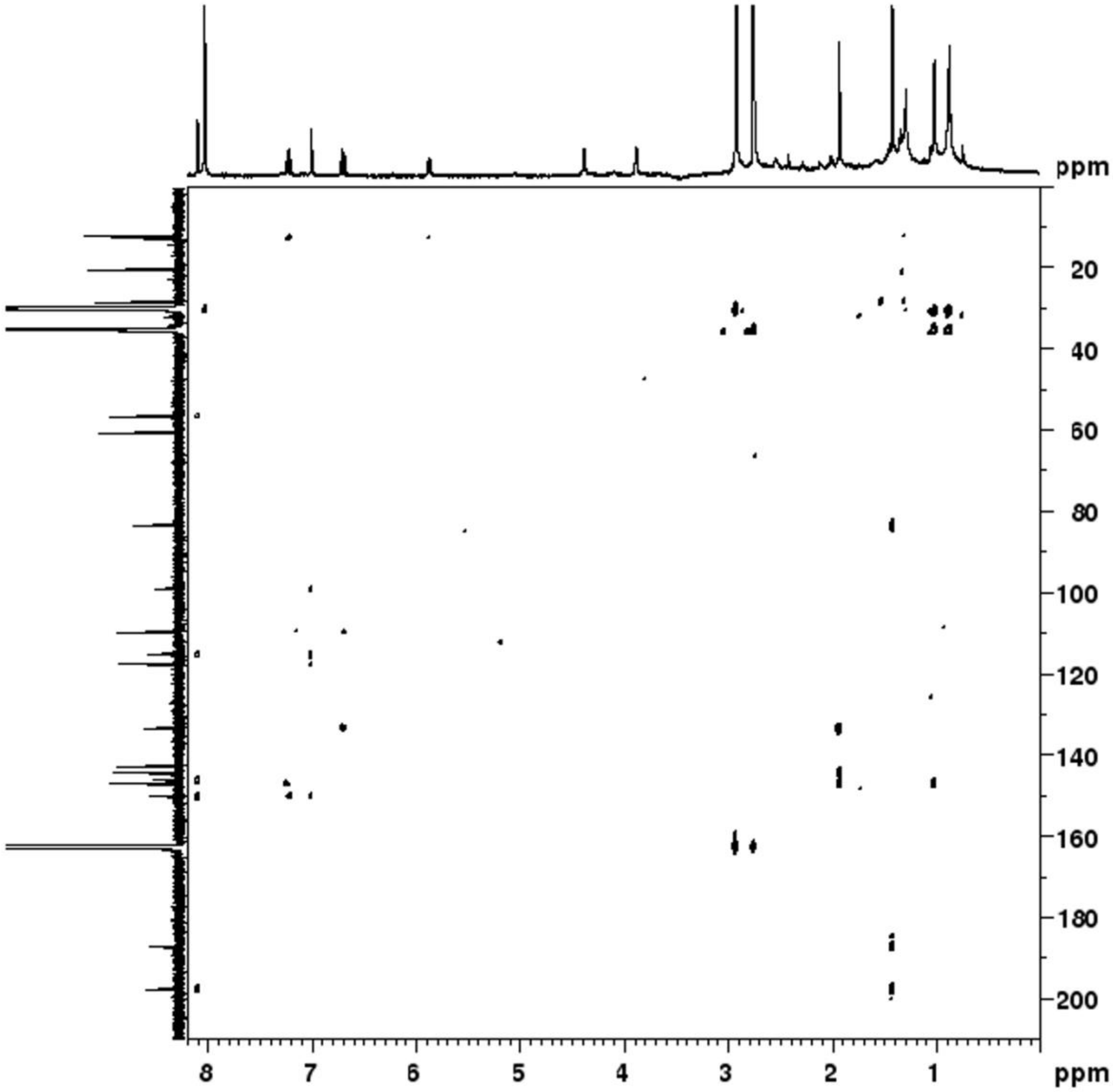

Figure S116: HRMS of compound **80**

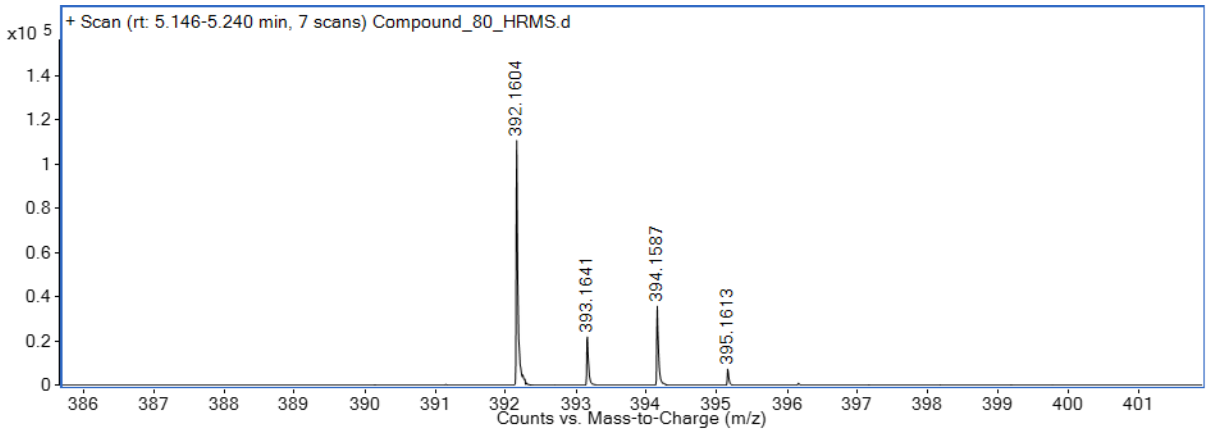

FigS117: Structural elucidation of isolated azaphilones, (a) COSY (in bod) and HMBC (arrow) correlation of compounds **63**, **74** and **80**

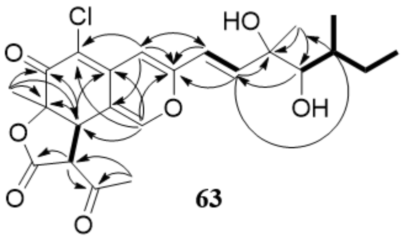

63

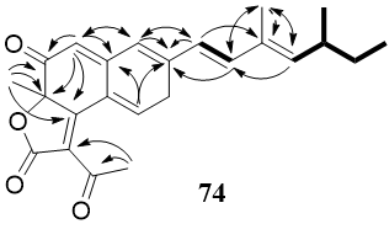

74

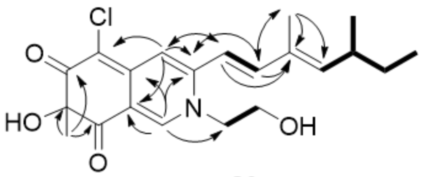

80

Figure S118: UV spectra in acetonitrile of isolated azaphilones **1**, **2**, **5**, **23**, **63**, **74**, **75** and **80**

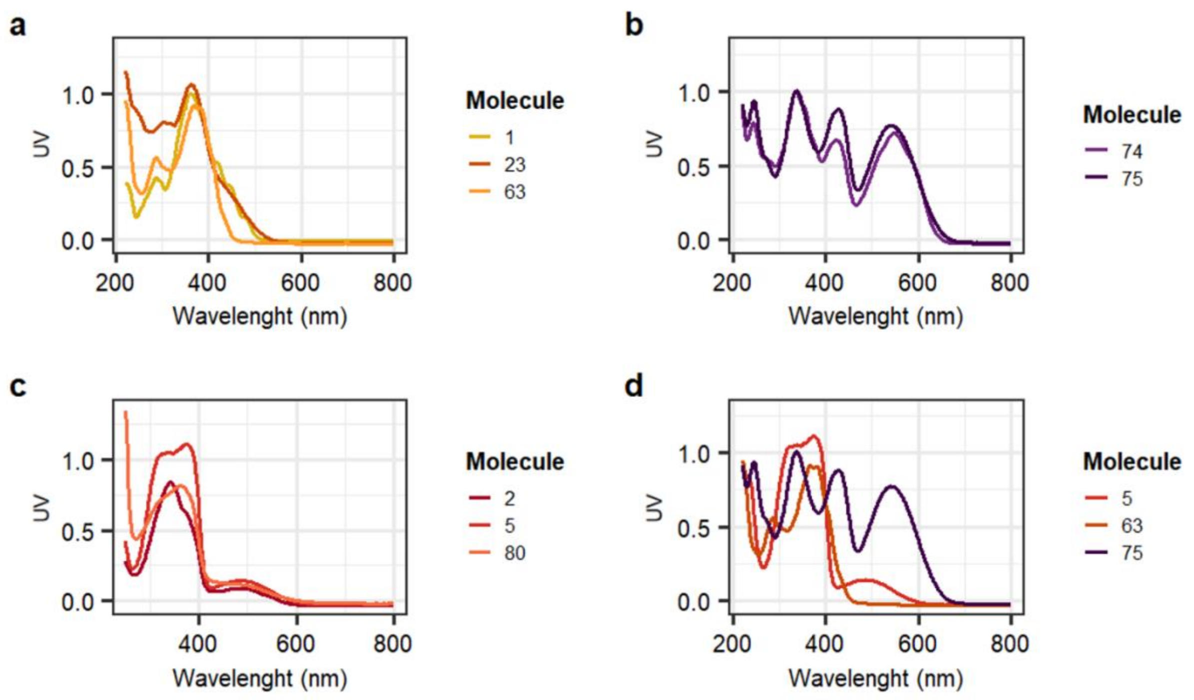

Figure S119: UV spectra in methanol of isolated azaphilones **1**, **2**, **5**, **23**, **63**, **74**, **75** and **80**

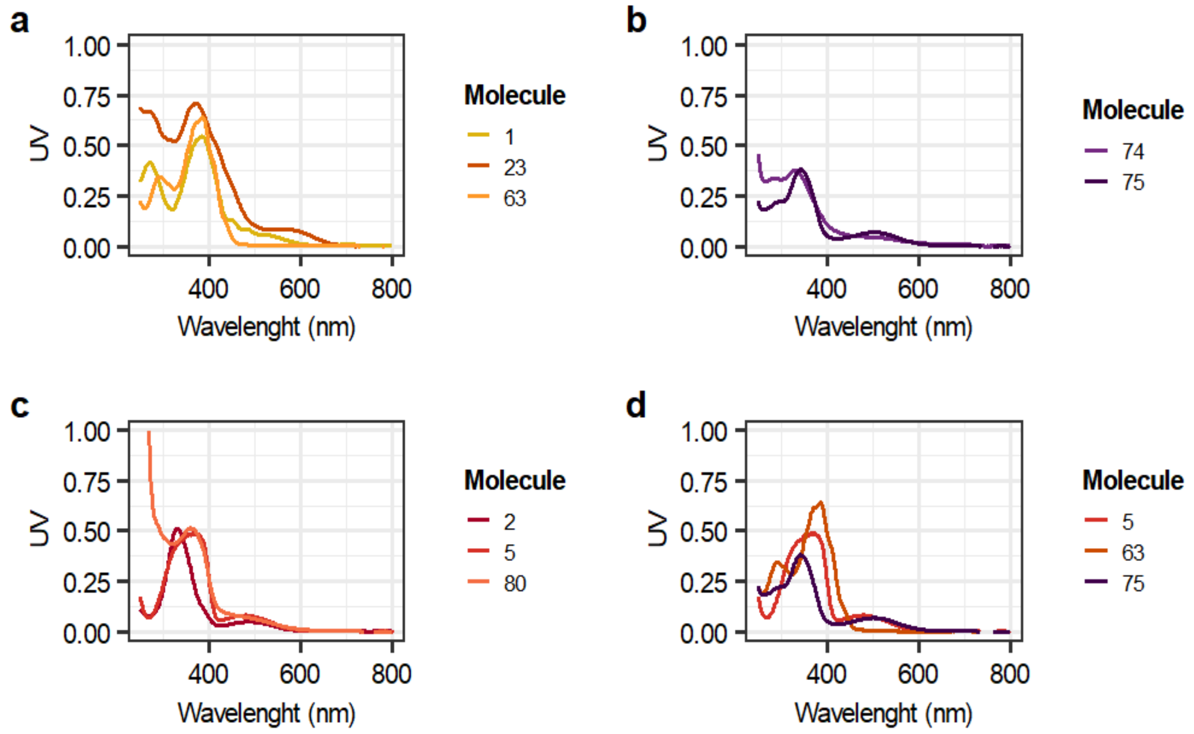

Figure S120: CD spectra of isolated azaphilones **2**, **5**, **23**, **63**, **74**, **75** and **80**

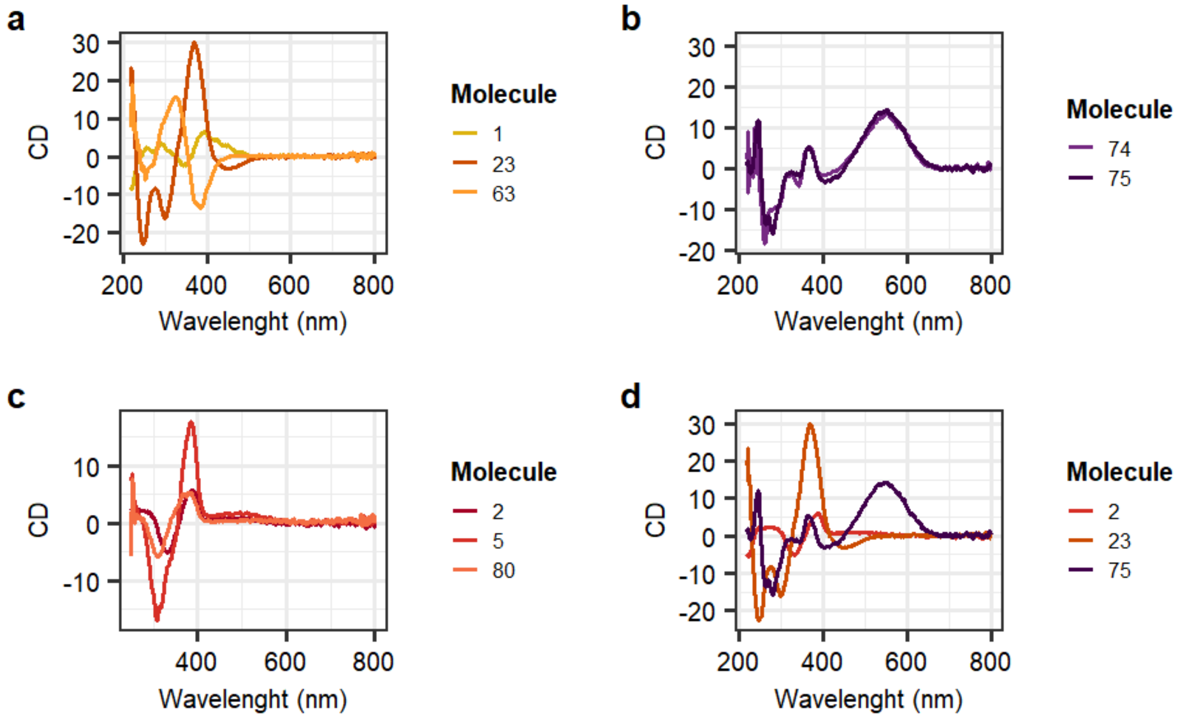

Figure S121: Absolute configuration of compound 1 and 5 based on (a) ORTEP drawing of compounds **1** and **5** with thermal ellipsoids drawn at the 50% probability level. Only the major disorder fragments are shown and for clarity and (b) compound **1** and **5** structure with asymmetric centers.

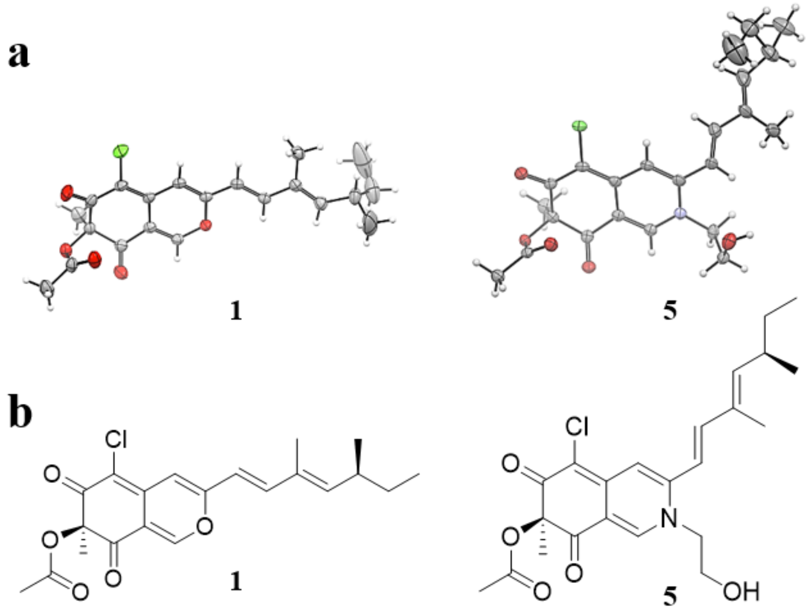

Table S3: Crystal data and structure refinement

Identification code		Compound 1	Compound 5
		A. Crystal Data	
**Empirical Formula**		$C_{21}H_{23}ClO_5$	$C_{23}H_{27}ClNO_5$
**Formula Weight**		390.84	432.90
**Crystal Color, Habit**		[clear light orange, prism]	[clear light orange, prism]
**Crystal Dimensions (mm^3)**		0.350 × 0.02 × 0.02	0.500 × 0.075 × 0.05
**Crystal System**		orthorhombic	orthorhombic
**Space Group**		$P2_12_12_1$	$P2_12_12_1$
**Unit cell dimensions**	a (Å)	7.1822 (3)	6.6642 (2)
	b (Å)	16.6035 (6)	8.5313 (2)
	c (Å)	34.6011 (13)	39.7195 (11)
	α (°)	90	90
	β (°)	90	90
	γ (°)	90	90
**Volume (Å^3)**		4126.2 (3)	2258.22 (11)
**Z value**		8	4
**Calculated density $D_{calc.}$ (g.cm^{-3})**		1.258	1.273
**Absorption coefficient μ (mm^{-1})**		0.213	0.202
**F (000)**		1648.0	916.0
		B. Intensity Measurements	
**Diffractometer**		Rigaku XtaLAB PRO	
**Radiation type**		Mo K_α	
**Wavelength (Å)**		0.71073	
**Voltage, Current (kV, mA)**		(50, 0.6)	
***T* (K)**		153 (10)	175 (10)
**2θ range for data collection (°)**		6.614 to 51.362	6.846 to 51.356
**Limiting indices**		$-8 \le h \le 8$,<br>$-20 \le k \le 17$,<br>$-42 \le l \le 42$	$-8 \le h \le 8$,<br>$-10 \le k \le 10$,<br>$-48 \le l \le 47$
**Reflections collected/unique**		39852/7808	19887/4177
**Completeness to θ full (%)**		99.7	96.7
		C. Structure Solution and Refinement	
**R_{int}**		0.0699	0.0361
**Absorption correction**		Semi-empirical from equivalents	
**Refinement method**		Full-matrix least-squares on F^2	
**Data/restraints/parameters**		7808/0/539	4177/0/328
**Goodness-of-fit on F^2**		1.033	1.065
**Final R indices [I>2σ(I)]**	R_1<br>wR_2	0.0424<br>0.0851	0.0336<br>0.0805
**R indices (all data)**	R_1<br>wR_2	0.0644<br>0.0910	0.0345<br>0.0810
Absolute structure parameters<br>**Flack Parameter**<br>**Hooft Parameter**		0.03 (3)<br>0.04 (3)	-0.007(19)<br>0.002 (17)
**Largest Δ peak and hole (e.Å^{-3})**		0.18/-0.16	0.27/-0.22
**CCDC Deposit Number**		2085749	2085750

Table S4: Fractional Atomic Coordinates (×10^4) and Equivalent Isotropic Displacement Parameters (Å^2×10^3) for compound **1**. U_{eq} is defined as 1/3 of the trace of the orthogonalised U_{ij}.

Atom	x	y	z	$U_{(eq)}$
Cl1	6025.5(11)	795.7(5)	5808.1(2)	35.8(2)
Cl1'	11014.2(15)	4548.7(5)	6737.2(3)	51.8(3)
O1'	10323(3)	1381.5(11)	6918.9(6)	31.0(5)
O4	7183(3)	3760.3(12)	5436.7(6)	29.0(5)
O1	5364(3)	2639.2(12)	7061.4(6)	34.3(5)
O5	9700(3)	3301.4(13)	5746.8(6)	35.7(5)
O4'	10687(3)	3898.6(12)	8183.5(6)	35.7(5)
O2	6970(3)	2143.4(14)	5288.3(6)	40.6(6)
O5'	13445(3)	3563.9(14)	7925.4(7)	45.1(6)
O3'	10225(4)	2304.3(14)	8031.8(7)	50.2(6)
O3	6721(3)	4213.6(14)	6180.0(6)	45.8(6)
O2'	10929(4)	4920.0(13)	7570.3(7)	52.8(6)
C4A'	10490(4)	3064.4(17)	7040.5(9)	26.1(7)
C4A	5807(4)	2059.2(17)	6295.1(9)	25.5(7)
C8A'	10255(4)	2491.2(17)	7353.7(9)	27.4(7)
C8A	5904(4)	2909.9(17)	6394.6(8)	26.4(6)
C3	5300(4)	1819.3(17)	6983.1(9)	28.0(7)
C9	5023(4)	1341.9(18)	7325.4(9)	31.2(7)
C4	5502(4)	1542.6(18)	6621.0(9)	29.2(7)
C19	9031(4)	3748.3(18)	5510.4(8)	27.5(7)
C6	6479(4)	2359.7(19)	5607.1(9)	28.0(7)
C5	6068(4)	1813.1(17)	5923.5(8)	27.3(7)
C1'	10181(4)	1698.9(18)	7277.7(9)	29.6(7)
C20	10047(4)	4340.4(19)	5267.9(9)	35.1(7)
C11'	10969(4)	308.4(18)	5816.2(9)	31.6(7)
C10'	10760(4)	701.1(18)	6188.2(8)	28.7(7)
C19'	12563(4)	3812.4(19)	8195.1(10)	36.4(8)
C1	5683(4)	3141.9(18)	6762.7(9)	32.7(7)
C4'	10611(4)	2709.8(17)	6664.3(9)	28.5(7)
C3'	10537(4)	1901.2(17)	6612.1(9)	27.2(7)
C8	6311(4)	3523.1(17)	6099.6(9)	29.3(7)
C5'	10619(4)	3873.5(17)	7112.9(9)	33.0(7)
C8'	10160(4)	2756.7(18)	7759.1(9)	32.4(7)
C9'	10695(4)	1504.8(18)	6244.9(9)	29.2(7)
C6'	10559(4)	4215(2)	7497.0(10)	36.1(8)
C10	4923(5)	1638.9(19)	7683.7(9)	34.1(7)
C7	6085(4)	3258.6(18)	5683.6(8)	29.1(7)
C11	4664(5)	1171.1(19)	8030.4(9)	38.6(8)
C12'	11143(5)	-499.4(19)	5808.1(10)	38.2(8)
C7'	9862(4)	3664.6(18)	7823.2(9)	30.9(7)
C17'	11034(5)	834(2)	5463.4(9)	40.4(8)
C18	4062(4)	3421(2)	5567.4(10)	43.9(9)
C13'	11486(5)	-1035(2)	5466.1(11)	49.6(10)
C18'	7772(4)	3805(2)	7872.6(11)	47.1(9)
C20'	13323(5)	4027(2)	8583.9(11)	55.3(10)
C12	4819(8)	1529(2)	8373.2(11)	70.4(13)
C14'	9805(8)	-1547(3)	5368.3(15)	85.1(16)
C16'	13198(8)	-1559(4)	5552.8(18)	116(2)
C15'	8200(8)	-1074(6)	5217(2)	157(4)
C17	4204(6)	293.3(19)	7981.5(10)	49.8(10)
C13A	5170(20)	1140(9)	8777(5)	53(4)
C16A	7216(19)	1248(9)	8937(5)	79(4)
C14A	3820(20)	1499(9)	9048(4)	66(4)
C15A	1816(18)	1353(8)	8936(3)	81(3)

Atom	x	y	z	$U_{(eq)}$
C15B	2260(20)	1301(6)	9360(3)	99(4)
C13B	4130(20)	1187(10)	8751(5)	58(4)
C16B	6030(30)	1031(11)	8952(6)	98(7)
C14B	2910(20)	1712(9)	8989(4)	61(4)

Table S5: Anisotropic Displacement Parameters (×10^4) for compound **1**. The anisotropic displacement factor exponent takes the form: $-2\pi^2[h^2a^{*2} \times U_{11}+2hka^* \times b^* \times U_{12}+ ...]$.

Atom	U_{11}	U_{22}	U_{33}	U_{23}	U_{13}	U_{12}
Cl1	45.9(4)	29.5(4)	32.0(4)	-6.1(3)	-0.6(4)	-2.4(4)
Cl1'	79.9(6)	27.6(4)	48.0(5)	9.5(4)	2.0(5)	-3.3(4)
O1'	41.8(12)	23.7(11)	27.3(12)	1.5(9)	0.1(9)	-2.6(9)
O4	24.9(10)	34.1(12)	28.0(12)	8.3(9)	-1.4(9)	2.1(9)
O1	52.1(13)	25.0(11)	25.8(12)	0.0(9)	6.1(10)	1.6(10)
O5	28.2(11)	43.5(13)	35.3(13)	12.4(11)	-3.7(10)	3.6(10)
O4'	32.4(12)	37.7(12)	37.0(13)	-9.5(10)	-0.2(10)	1.4(9)
O2	54.7(13)	42.6(14)	24.7(13)	-3.0(11)	5.1(11)	-3.4(11)
O5'	32.8(12)	53.2(15)	49.3(16)	-16.0(12)	0.3(11)	5.3(11)
O3'	80.1(17)	38.5(13)	32.2(14)	0.5(12)	-3.9(13)	-8.6(13)
O3	70.9(16)	28.8(13)	37.7(14)	2.2(11)	15.1(12)	-1.4(12)
O2'	76.9(17)	25.7(13)	55.8(16)	-7.8(11)	2.1(14)	0.2(13)
C4A'	21.7(15)	24.5(15)	32.2(18)	3.4(13)	-1.9(13)	1.5(12)
C4A	20.0(14)	27.0(15)	29.6(17)	0.5(13)	-0.8(13)	0.7(12)
C8A'	25.3(15)	26.9(16)	30.1(17)	2.3(13)	-1.7(14)	-1.1(12)
C8A	25.0(14)	28.3(15)	25.9(17)	-0.1(13)	2.2(13)	0.5(13)
C3	29.2(16)	23.1(16)	31.7(18)	0.4(14)	-1.0(14)	1.7(12)
C9	35.2(15)	27.3(16)	31.1(19)	3.8(14)	3.0(15)	-0.4(14)
C4	31.7(16)	24.5(15)	31.6(18)	-1.7(14)	-2.1(13)	-1.8(13)
C19	25.7(15)	30.3(16)	26.6(17)	-1.0(14)	-0.5(14)	5.4(14)
C6	22.8(15)	36.1(17)	25.0(18)	-0.8(14)	-1.7(13)	1.2(13)
C5	25.3(15)	28.4(16)	28.2(17)	-3.6(13)	-1.8(14)	-0.3(14)
C1'	34.0(16)	28.3(16)	26.6(17)	3.2(14)	-0.8(14)	-1.1(14)
C20	32.2(16)	38.6(19)	34.5(18)	9.4(15)	0.6(15)	-1.1(15)
C11'	30.3(15)	35.4(18)	29.1(17)	-2.5(14)	-0.3(15)	-0.2(14)
C10'	26.3(15)	33.2(17)	26.5(16)	3.8(13)	1.5(13)	-2.2(13)
C19'	33.1(18)	34.0(18)	42(2)	-11.0(16)	-3.0(16)	-0.3(14)
C1	41.2(18)	21.9(15)	34.9(19)	2.6(14)	3.1(15)	3.0(13)
C4'	28.7(16)	27.8(16)	29.0(18)	5.7(13)	-2.1(13)	0.9(12)
C3'	23.9(15)	28.3(16)	29.4(18)	5.2(14)	-0.3(13)	-0.5(12)
C8	30.0(16)	24.1(17)	33.9(18)	1.9(13)	6.0(14)	5.6(13)
C5'	37.4(18)	25.1(16)	36.7(19)	6.5(14)	-1.6(15)	0.5(13)
C8'	33.1(16)	30.0(17)	34.1(19)	2.0(15)	-3.2(15)	-4.1(14)
C9'	29.1(16)	31.2(17)	27.1(17)	4.6(13)	-1.7(13)	0.5(13)
C6'	33.7(18)	28.0(17)	47(2)	-2.7(16)	-1.8(14)	8.2(14)
C10	44.9(17)	25.1(16)	32.3(19)	3.1(14)	6.5(16)	-1.1(14)
C7	25.4(15)	34.1(16)	27.9(17)	5.9(13)	-0.3(14)	3.8(14)
C11	59(2)	26.7(16)	29.6(19)	1.4(14)	6.3(16)	0.8(15)
C12'	42.9(18)	39.0(18)	32.7(18)	-2.0(15)	4.9(17)	0.3(16)
C7'	29.7(15)	30.9(16)	32.1(18)	-5.2(14)	-2.2(14)	1.6(14)
C17'	43.5(17)	47.2(19)	30.4(18)	1.3(15)	5.1(16)	1.9(18)
C18	24.9(16)	58(2)	49(2)	10.0(18)	-4.6(16)	3.1(17)
C13'	62(2)	39(2)	48(2)	-13.3(17)	12.0(19)	1.8(18)
C18'	34.5(18)	61(2)	46(2)	-8.5(19)	-0.1(16)	2.9(17)
C20'	52(2)	65(3)	49(2)	-18(2)	-9.4(18)	-2.8(19)
C12	145(4)	31(2)	35(2)	2.4(17)	12(3)	-15(2)
C14'	96(4)	80(3)	79(3)	-39(3)	21(3)	-34(3)

Atom	U_{11}	U_{22}	U_{33}	U_{23}	U_{13}	U_{12}
C16'	119(5)	120(5)	108(5)	-58(4)	-16(4)	70(4)
C15'	64(3)	259(10)	147(6)	-138(7)	-17(4)	12(5)
C17	83(3)	31.6(18)	34(2)	4.6(15)	5(2)	-12.4(18)
C13A	102(13)	33(6)	25(6)	-6(5)	-3(10)	1(9)
C16A	105(10)	76(8)	56(8)	-10(6)	-21(8)	-4(8)
C14A	109(11)	50(8)	40(7)	6(5)	0(8)	4(7)
C15A	104(9)	80(8)	60(7)	2(6)	5(6)	6(7)
C15B	174(11)	73(7)	50(6)	19(5)	50(7)	-1(7)
C13B	107(11)	39(6)	29(7)	-1(4)	2(9)	-6(9)
C16B	150(20)	93(12)	54(9)	13(9)	21(14)	44(12)
C14B	94(12)	49(8)	38(7)	10(5)	19(8)	0(7)

Table S6: Bond Lengths in Å for compound **1**.

Atom	Atom	Length/Å	Atom	Atom	Length/Å
Cl1	C5	1.736(3)	C6	C5	1.452(4)
Cl1'	C5'	1.740(3)	C6	C7	1.542(4)
O1'	C1'	1.353(4)	C11'	C10'	1.451(4)
O1'	C3'	1.377(3)	C11'	C12'	1.347(4)
O4	C19	1.352(4)	C11'	C17'	1.501(4)
O4	C7	1.430(4)	C10'	C9'	1.350(4)
O1	C3	1.389(3)	C19'	C20'	1.495(5)
O1	C1	1.348(4)	C4'	C3'	1.356(4)
O5	C19	1.204(3)	C3'	C9'	1.435(4)
O4'	C19'	1.356(4)	C8	C7	1.514(4)
O4'	C7'	1.434(4)	C5'	C6'	1.445(5)
O2	C6	1.212(4)	C8'	C7'	1.539(4)
O5'	C19'	1.201(4)	C6'	C7'	1.536(5)
O3'	C8'	1.207(4)	C10	C11	1.441(4)
O3	C8	1.216(4)	C7	C18	1.531(4)
O2'	C6'	1.227(4)	C11	C12	1.331(5)
C4A'	C8A'	1.452(4)	C11	C17	1.504(4)
C4A'	C4'	1.431(4)	C12'	C13'	1.501(5)
C4A'	C5'	1.370(4)	C7'	C18'	1.528(4)
C4A	C8A	1.455(4)	C13'	C14'	1.515(6)
C4A	C4	1.434(4)	C13'	C16'	1.535(6)
C4A	C5	1.362(4)	C12	C13A	1.558(18)
C8A'	C1'	1.343(4)	C12	C13B	1.510(17)
C8A'	C8'	1.472(4)	C14'	C15'	1.490(9)
C8A	C1	1.340(4)	C13A	C16A	1.58(2)
C8A	C8	1.471(4)	C13A	C14A	1.47(2)
C3	C9	1.439(4)	C14A	C15A	1.511(19)
C3	C4	1.343(4)	C15B	C14B	1.525(16)
C9	C10	1.336(4)	C13B	C16B	1.56(2)
C19	C20	1.484(4)	C13B	C14B	1.48(2)

Table S7: Bond Angles in for compound **1**.

Atom	Atom	Atom	Angle/°	Atom	Atom	Atom	Angle/°
C1'	O1'	C3'	118.2(2)	O3	C8	C7	121.1(3)
C19	O4	C7	114.8(2)	C8A	C8	C7	116.0(3)
C1	O1	C3	117.6(2)	C4A'	C5'	Cl1'	120.4(3)
C19'	O4'	C7'	114.1(2)	C4A'	C5'	C6'	123.4(3)
C4'	C4A'	C8A'	114.6(2)	C6'	C5'	Cl1'	116.1(2)
C5'	C4A'	C8A'	120.9(3)	O3'	C8'	C8A'	123.8(3)
C5'	C4A'	C4'	124.4(3)	O3'	C8'	C7'	120.2(3)
C4	C4A	C8A	113.7(3)	C8A'	C8'	C7'	115.9(3)
C5	C4A	C8A	120.5(3)	C10'	C9'	C3'	125.8(3)
C5	C4A	C4	125.7(3)	O2'	C6'	C5'	123.9(3)
C4A'	C8A'	C8'	121.4(3)	O2'	C6'	C7'	119.1(3)
C1'	C8A'	C4A'	120.0(3)	C5'	C6'	C7'	116.9(3)
C1'	C8A'	C8'	118.6(3)	C9	C10	C11	125.5(3)
C4A	C8A	C8	121.1(3)	O4	C7	C6	111.1(2)
C1	C8A	C4A	119.9(3)	O4	C7	C8	109.9(2)
C1	C8A	C8	119.0(3)	O4	C7	C18	105.3(2)
O1	C3	C9	112.6(3)	C8	C7	C6	115.1(2)
C4	C3	O1	120.9(3)	C8	C7	C18	107.5(3)
C4	C3	C9	126.5(3)	C18	C7	C6	107.4(3)
C10	C9	C3	124.6(3)	C10	C11	C17	117.2(3)
C3	C4	C4A	123.1(3)	C12	C11	C10	119.4(3)
O4	C19	C20	111.5(3)	C12	C11	C17	123.5(3)
O5	C19	O4	121.9(3)	C11'	C12'	C13'	128.5(3)
O5	C19	C20	126.6(3)	O4'	C7'	C8'	109.5(2)
O2	C6	C5	124.0(3)	O4'	C7'	C6'	110.1(2)
O2	C6	C7	119.8(3)	O4'	C7'	C18'	105.5(3)
C5	C6	C7	116.0(3)	C6'	C7'	C8'	115.6(3)
C4A	C5	Cl1	120.4(2)	C18'	C7'	C8'	107.6(3)
C4A	C5	C6	123.5(3)	C18'	C7'	C6'	108.2(3)
C6	C5	Cl1	116.0(2)	C12'	C13'	C14'	112.2(3)
C8A'	C1'	O1'	123.9(3)	C12'	C13'	C16'	108.2(3)
C10'	C11'	C17'	117.6(3)	C14'	C13'	C16'	111.4(4)
C12'	C11'	C10'	118.4(3)	C11	C12	C13A	128.8(6)
C12'	C11'	C17'	124.0(3)	C11	C12	C13B	125.2(7)
C9'	C10'	C11'	125.2(3)	C15'	C14'	C13'	113.5(5)
O4'	C19'	C20'	111.4(3)	C12	C13A	C16A	114.6(10)
O5'	C19'	O4'	122.5(3)	C14A	C13A	C12	107.4(12)
O5'	C19'	C20'	126.1(3)	C14A	C13A	C16A	109.9(13)
C8A	C1	O1	124.8(3)	C13A	C14A	C15A	113.3(13)
C3'	C4'	C4A'	121.7(3)	C12	C13B	C16B	99.3(11)
O1'	C3'	C9'	113.8(2)	C14B	C13B	C12	117.0(12)
C4'	C3'	O1'	121.5(3)	C14B	C13B	C16B	111.6(14)
C4'	C3'	C9'	124.7(3)	C13B	C14B	C15B	112.7(12)
O3	C8	C8A	122.8(3)				

Table S8: Torsion Angles in ° for compound **1**.

A	B	C	D	Angle/°	A	B	C	D	Angle/°
Cl1'	C5'	C6'	O2'	-6.7(4)	C5	C4A	C4	C3	178.3(3)
Cl1'	C5'	C6'	C7'	169.1(2)	C5	C6	C7	O4	156.4(2)
O1'	C3'	C9'	C10'	-4.2(4)	C5	C6	C7	C8	30.7(4)
O1	C3	C9	C10	-2.9(4)	C5	C6	C7	C18	-88.9(3)
O1	C3	C4	C4A	0.5(4)	C1'	O1'	C3'	C4'	0.1(4)
O2	C6	C5	Cl1	-8.0(4)	C1'	O1'	C3'	C9'	179.3(2)
O2	C6	C5	C4A	169.1(3)	C1'	C8A'	C8'	O3'	8.5(5)
O2	C6	C7	O4	-28.7(4)	C1'	C8A'	C8'	C7'	-168.4(3)
O2	C6	C7	C8	-154.4(3)	C11'	C10'	C9'	C3'	-178.3(3)
O2	C6	C7	C18	85.9(3)	C11'	C12'	C13'	C14'	-110.5(5)
O3'	C8'	C7'	O4'	31.6(4)	C11'	C12'	C13'	C16'	126.2(5)
O3'	C8'	C7'	C6'	156.5(3)	C10'	C11'	C12'	C13'	-176.1(3)
O3'	C8'	C7'	C18'	-82.6(4)	C19'	O4'	C7'	C8'	64.3(3)
O3	C8	C7	O4	26.3(4)	C19'	O4'	C7'	C6'	-63.7(3)
O3	C8	C7	C6	152.6(3)	C19'	O4'	C7'	C18'	179.8(3)
O3	C8	C7	C18	-87.8(3)	C1	O1	C3	C9	177.8(2)
O2'	C6'	C7'	O4'	-32.2(4)	C1	O1	C3	C4	-1.6(4)
O2'	C6'	C7'	C8'	-156.8(3)	C1	C8A	C8	O3	10.8(4)
O2'	C6'	C7'	C18'	82.6(4)	C1	C8A	C8	C7	-165.8(3)
C4A'	C8A'	C1'	O1'	0.3(4)	C4'	C4A'	C8A'	C1'	0.5(4)
C4A'	C8A'	C8'	O3'	-169.3(3)	C4'	C4A'	C8A'	C8'	178.3(2)
C4A'	C8A'	C8'	C7'	13.8(4)	C4'	C4A'	C5'	Cl1'	-1.7(4)
C4A'	C4'	C3'	O1'	0.7(4)	C4'	C4A'	C5'	C6'	-177.7(3)
C4A'	C4'	C3'	C9'	-178.4(2)	C4'	C3'	C9'	C10'	174.9(3)
C4A'	C5'	C6'	O2'	169.5(3)	C3'	O1'	C1'	C8A'	-0.6(4)
C4A'	C5'	C6'	C7'	-14.7(4)	C8	C8A	C1	O1	-177.8(3)
C4A	C8A	C1	O1	-0.1(5)	C5'	C4A'	C8A'	C1'	-178.2(3)
C4A	C8A	C8	O3	-166.9(3)	C5'	C4A'	C8A'	C8'	-0.4(4)
C4A	C8A	C8	C7	16.5(4)	C5'	C4A'	C4'	C3'	177.7(3)
C8A'	C4A'	C4'	C3'	-1.0(4)	C5'	C6'	C7'	O4'	151.8(2)
C8A'	C4A'	C5'	Cl1'	176.9(2)	C5'	C6'	C7'	C8'	27.1(4)
C8A'	C4A'	C5'	C6'	0.8(4)	C5'	C6'	C7'	C18'	-93.4(3)
C8A'	C8'	C7'	O4'	-151.4(2)	C8'	C8A'	C1'	O1'	-177.5(3)
C8A'	C8'	C7'	C6'	-26.5(4)	C10	C11	C12	C13A	-160.8(9)
C8A'	C8'	C7'	C18'	94.4(3)	C10	C11	C12	C13B	163.7(8)
C8A	C4A	C4	C3	0.8(4)	C7	O4	C19	O5	5.4(4)
C8A	C4A	C5	Cl1	177.9(2)	C7	O4	C19	C20	-174.6(2)
C8A	C4A	C5	C6	0.9(4)	C7	C6	C5	Cl1	166.7(2)
C8A	C8	C7	O4	-157.0(2)	C7	C6	C5	C4A	-16.2(4)
C8A	C8	C7	C6	-30.7(4)	C11	C12	C13A	C16A	104.7(12)
C8A	C8	C7	C18	88.9(3)	C11	C12	C13A	C14A	-132.9(10)
C3	O1	C1	C8A	1.4(4)	C11	C12	C13B	C16B	112.2(11)
C3	C9	C10	C11	-179.4(3)	C11	C12	C13B	C14B	-127.7(13)
C9	C3	C4	C4A	-178.8(3)	C12'	C11'	C10'	C9'	175.4(3)
C9	C10	C11	C12	171.7(4)	C12'	C13'	C14'	C15'	68.3(5)

A	B	C	D	Angle/˚	A	B	C	D	Angle/˚
C9	C10	C11	C17	-9.0(5)	C7'	O4'	C19'	O5'	0.2(4)
C4	C4A	C8A	C1	-1.1(4)	C7'	O4'	C19'	C20'	-177.1(3)
C4	C4A	C8A	C8	176.6(2)	C17'	C11'	C10'	C9'	-2.9(4)
C4	C4A	C5	Cl1	0.7(4)	C17'	C11'	C12'	C13'	2.1(6)
C4	C4A	C5	C6	-176.3(3)	C12	C13A	C14A	C15A	60.2(15)
C4	C3	C9	C10	176.4(3)	C12	C13B	C14B	C15B	178.8(12)
C19	O4	C7	C6	-68.3(3)	C16'	C13'	C14'	C15'	-170.2(5)
C19	O4	C7	C8	60.3(3)	C17	C11	C12	C13A	20.0(11)
C19	O4	C7	C18	175.8(2)	C17	C11	C12	C13B	-15.5(10)
C5	C4A	C8A	C1	-178.6(3)	C16A	C13A	C14A	C15A	-174.5(12)
C5	C4A	C8A	C8	-0.9(4)	C16B	C13B	C14B	C15B	-67.9(18)

Table S9: Hydrogen Bonds for compound **1**.

D	H	A	d(D-H)/Å	d(H-A)/Å	d(D-A)/Å	D-H-A/°
C1'	H1'	O2'[1]	0.95	2.46	3.104(4)	124.8

[1]2-X,-1/2+Y,3/2-Z

Table S10: Hydrogen Fractional Atomic Coordinates (×10^4) and Equivalent Isotropic Displacement Parameters (Å^2×10^3) for compound **1**. U_{eq} is defined as 1/3 of the trace of the orthogonalised U_{ij}.

Atom	x	y	z	U(eq)
H9	4903.61	775.76	7294.51	37
H4	5440.06	977.86	6578.65	35
H1'	10020.05	1339.53	7488.74	36
H20A	10787.05	4054.11	5073.52	53
H20B	10873.98	4663.17	5431.54	53
H20C	9151.88	4694.73	5138.43	53
H10'	10660	365.63	6409.65	34
H1	5755.84	3701.8	6817.15	39
H4'	10746.31	3050.09	6445.36	34
H9'	10759.46	1837.79	6022.04	35
H10	5030.41	2206.03	7712.28	41
H12'	11035.14	-764.3	6050.39	46
H17D	9907.72	1166.39	5452.5	61
H17E	11102.63	495.31	5231.82	61
H17F	12132.81	1182.58	5475.6	61
H18A	3225.41	3129.38	5742.07	66
H18B	3858.72	3236.37	5301.62	66
H18C	3808.41	3999.39	5584.02	66
H13'	11776.45	-687.54	5237.98	60
H18D	7120.37	3640.07	7636.55	71
H18E	7540.35	4377.22	7921.49	71
H18F	7314.6	3486.04	8091.33	71
H20D	12725.5	4521.08	8676.38	83
H20E	14670.15	4112.46	8564.43	83
H20F	13072.84	3587.37	8765.67	83
H14E	10169.93	-1952.53	5172.53	102
H14F	9406.4	-1840.72	5603.27	102
H16G	14292.41	-1213.04	5585.31	174
H16H	13408.73	-1931.84	5337.72	174
H16I	12984.59	-1865.59	5790.66	174
H15G	7868.86	-653.18	5403.86	235
H15H	7132.43	-1432.15	5178.42	235
H15I	8543.55	-824.52	4970.8	235
H17A	3050.28	239.44	7832.6	75
H17B	4041.46	44.58	8236.13	75
H17C	5221.33	23.84	7843.91	75
H13A	4898.3	550.8	8756.88	64
H16A	8098.1	986.03	8761.84	118
H16B	7307.04	1002.08	9194.15	118
H16C	7510.01	1823.31	8955.72	118
H12	5150(50)	2120(30)	8383(11)	63(11)
H14A	4042.43	2086.57	9062.35	79
H14B	4037.58	1272	9308.94	79
H15A	1570.16	772.73	8930.67	122
H15B	1583.88	1581.41	8679.03	122
H15C	992.21	1611.19	9124.7	122
H15D	1489.38	833.22	9294.54	149
H15E	1525.23	1681.93	9513.63	149
H15F	3343.99	1125.18	9509.22	149
H13B	3487.51	662.19	8702.89	70
H16D	6696.34	1542.49	8984.37	148
H16E	6780.17	665.61	8793.16	148

Atom	x	y	z	U(eq)
H16F	5820.57	786.73	9206.52	148
H14C	1807	1868.58	8834.84	73
H14D	3595.71	2208.96	9056.88	73

Table S11: Fractional Atomic Coordinates (×10[4]) and Equivalent Isotropic Displacement Parameters (Å[2]×10[3]) for compound **5**. U_{eq} is defined as 1/3 of the trace of the orthogonalised U_{ij}.

Atom	x	y	z	U(eq)
Cl1	-1240.2(9)	3123.3(7)	6019.6(2)	31.13(15)
O4	-143(2)	-58.5(18)	7093.4(4)	23.6(3)
O2	-2162(3)	634(2)	6520.5(5)	31.5(4)
O3	3498(3)	1320(2)	7198.9(5)	32.6(4)
O5	-992(3)	2435.3(19)	7197.5(5)	29.4(4)
O1'	6140(3)	8011(2)	6876.8(5)	36.5(4)
N1	5664(3)	4860(2)	6585.4(5)	25.5(4)
C6	-491(3)	1251(3)	6551.1(6)	23.2(5)
C5	269(4)	2488(3)	6351.9(6)	23.8(5)
C8	2667(3)	1575(3)	6933.2(6)	23.8(5)
C4A	2057(3)	3279(3)	6422.3(6)	22.9(4)
C3	4524(4)	5365(3)	6314.0(6)	28.4(5)
C1	5015(3)	3638(3)	6772.0(6)	23.4(5)
C8A	3279(3)	2848(3)	6704.1(6)	22.4(5)
C19	-1099(4)	1067(3)	7273.0(6)	22.9(5)
C4	2773(4)	4596(3)	6237.9(6)	27.9(5)
C1'	7612(4)	5567(3)	6685.7(7)	29.4(5)
C20	-2249(4)	384(3)	7559.2(7)	31.3(5)
C2'	7384(4)	6741(3)	6967.5(6)	28.7(5)
C7	963(3)	525(3)	6808.5(6)	23.7(5)
C10	4565(4)	7115(3)	5820.2(8)	37.3(6)
C9	5211(5)	6719(4)	6120.0(7)	41.5(7)
C18	1909(4)	-911(3)	6639.9(7)	32.8(6)
C11	5079(5)	8465(3)	5617.6(7)	43.9(8)
C13	4384(6)	10001(4)	5074.4(9)	52.0(8)
C12	4199(5)	8693(4)	5323.0(10)	56.6(9)
C15A	717(17)	10843(13)	5226(3)	100(4)
C14A	2073(11)	10371(8)	4936(2)	56.0(17)
C17A	5817(14)	9883(8)	5786.4(16)	59(2)
C17B	7337(11)	9071(8)	5707.9(16)	45.7(15)
C14B	3234(11)	11476(9)	5150.9(19)	56.2(18)
C15B	3620(16)	12396(11)	5471(2)	77(2)
C16B	4207(17)	9286(10)	4758(2)	61(2)
C16A	5535(17)	9604(12)	4749(2)	65(2)

Table S12: Anisotropic Displacement Parameters (×10[4]) for compound **5**. The anisotropic displacement factor exponent takes the form: $-2\pi^2[h^2a^{*2} \times U_{11} + 2hka^* \times b^* \times U_{12} + \ldots]$.

Atom	U_{11}	U_{22}	U_{33}	U_{23}	U_{13}	U_{12}
Cl1	36.1(3)	36.2(3)	21.1(3)	4.5(2)	-6.3(2)	-2.1(3)
O4	26.2(8)	21.0(7)	23.6(8)	3.0(6)	-0.5(6)	-0.7(7)
O2	28.3(9)	37.1(9)	29.1(10)	5.2(7)	-4.5(7)	-5.6(8)
O3	31.6(9)	35.9(9)	30.3(9)	8.1(7)	-7.9(8)	-4.0(7)
O5	33.0(9)	24.5(8)	30.7(9)	0.4(7)	0.7(8)	1.5(7)
O1'	28.8(9)	30.3(9)	50.5(12)	-2.4(8)	-3.2(8)	1.4(8)
N1	26.7(10)	26.4(10)	23.3(10)	-3.3(8)	3.2(8)	-3.5(8)
C6	24.3(11)	25.1(11)	20.3(11)	-1.9(9)	-0.6(9)	1.0(9)
C5	27.8(12)	26.9(11)	16.6(11)	-0.7(9)	-2.2(9)	3.9(9)
C8	21.3(11)	22.8(11)	27.2(12)	0.2(9)	-0.1(9)	2.8(9)
C4A	26.9(11)	23.7(10)	18.1(10)	-2.3(9)	3.2(8)	2.0(9)
C3	37.1(13)	29.3(12)	18.7(12)	-2.9(9)	5.1(9)	-4.8(10)
C1	24.0(11)	24.2(11)	22.2(11)	-0.7(9)	2.0(9)	3.1(9)
C8A	23.7(11)	22.4(11)	21.1(11)	-2.1(8)	2.0(8)	3.0(8)
C19	20.5(10)	25.8(11)	22.3(11)	0.9(9)	-4.9(9)	0.0(9)
C4	34.6(12)	31.4(12)	17.7(11)	2.5(9)	-0.6(10)	-3.1(10)
C1'	23.9(11)	29.6(12)	34.7(14)	-1.5(10)	4.9(10)	-4.6(10)
C20	30.4(12)	36.6(13)	26.9(13)	2.7(10)	1.6(10)	-1.5(11)
C2'	26.0(12)	28.5(12)	31.5(13)	-0.7(11)	-3.1(10)	-4.3(10)
C7	25.5(11)	23.0(10)	22.5(11)	2.1(9)	1.6(9)	0.4(9)
C10	39.0(14)	33.1(13)	39.7(16)	9.5(12)	-2.2(12)	-9.2(12)
C9	58.6(17)	44.9(15)	21.0(12)	0.5(11)	-0.1(12)	-26.7(15)
C18	33.2(13)	28.2(12)	37.0(15)	-4.3(10)	2.1(11)	4.2(10)
C11	76(2)	33.5(14)	22.5(13)	-0.5(11)	8.3(13)	-19.4(14)
C13	69(2)	42.3(16)	45.1(19)	16.8(14)	8.3(16)	-2.2(16)
C12	52.4(19)	51.9(18)	65(2)	32.2(16)	-12.1(16)	-15.3(15)
C15A	89(7)	77(6)	135(10)	31(6)	43(7)	35(5)
C14A	62(4)	44(3)	62(4)	11(3)	-11(3)	2(3)
C17A	102(6)	42(3)	32(3)	8(3)	-12(3)	-38(4)
C17B	53(4)	47(3)	37(3)	14(3)	-4(3)	-19(3)
C14B	55(4)	63(4)	51(4)	22(3)	-3(3)	-1(3)
C15B	90(6)	77(5)	65(5)	2(4)	10(5)	18(5)
C16B	88(6)	53(4)	43(4)	18(3)	-18(4)	-10(4)
C16A	87(6)	64(5)	43(4)	18(4)	12(4)	10(5)

Table S13: Bond Lengths in Å for compound **5**.

Atom	Atom	Length/Å	Atom	Atom	Length/Å
Cl1	C5	1.745(2)	C3	C9	1.462(4)
O4	C19	1.355(3)	C1	C8A	1.366(3)
O4	C7	1.440(3)	C19	C20	1.490(3)
O2	C6	1.237(3)	C1'	C2'	1.509(4)
O3	C8	1.212(3)	C7	C18	1.532(3)
O5	C19	1.207(3)	C10	C9	1.310(4)
O1'	C2'	1.412(3)	C10	C11	1.447(4)
N1	C3	1.387(3)	C11	C12	1.323(5)
N1	C1	1.350(3)	C11	C17A	1.468(7)
N1	C1'	1.486(3)	C11	C17B	1.631(7)
C6	C5	1.413(3)	C13	C12	1.495(4)
C6	C7	1.539(3)	C13	C14A	1.665(8)
C5	C4A	1.398(3)	C13	C14B	1.505(8)
C8	C8A	1.474(3)	C13	C16B	1.403(9)
C8	C7	1.529(3)	C13	C16A	1.541(9)
C4A	C8A	1.432(3)	C15A	C14A	1.518(13)
C4A	C4	1.423(3)	C14B	C15B	1.516(13)
C3	C4	1.372(4)			

Table S14: Bond Angles in ° for compound **5**.

Atom	Atom	Atom	Angle/°	Atom	Atom	Atom	Angle/°
C19	O4	C7	114.17(17)	C3	C4	C4A	123.3(2)
C3	N1	C1'	124.1(2)	N1	C1'	C2'	112.3(2)
C1	N1	C3	119.4(2)	O1'	C2'	C1'	112.3(2)
C1	N1	C1'	116.5(2)	O4	C7	C6	109.82(18)
O2	C6	C5	125.8(2)	O4	C7	C8	109.15(19)
O2	C6	C7	117.4(2)	O4	C7	C18	106.12(19)
C5	C6	C7	116.5(2)	C8	C7	C6	116.59(19)
C6	C5	Cl1	116.66(18)	C8	C7	C18	107.74(19)
C4A	C5	Cl1	119.51(18)	C18	C7	C6	106.89(19)
C4A	C5	C6	123.7(2)	C9	C10	C11	129.3(3)
O3	C8	C8A	122.9(2)	C10	C9	C3	125.4(3)
O3	C8	C7	121.1(2)	C10	C11	C17A	118.8(3)
C8A	C8	C7	115.9(2)	C10	C11	C17B	110.4(3)
C5	C4A	C8A	121.1(2)	C12	C11	C10	120.3(3)
C5	C4A	C4	124.3(2)	C12	C11	C17A	115.6(4)
C4	C4A	C8A	114.5(2)	C12	C11	C17B	123.8(3)
N1	C3	C9	118.9(2)	C12	C13	C14A	106.4(4)
C4	C3	N1	119.2(2)	C12	C13	C14B	116.7(4)
C4	C3	C9	121.9(2)	C12	C13	C16A	115.6(4)
N1	C1	C8A	123.0(2)	C16B	C13	C12	105.1(4)
C4A	C8A	C8	121.0(2)	C16B	C13	C14B	120.1(5)
C1	C8A	C8	118.4(2)	C16A	C13	C14A	103.0(5)
C1	C8A	C4A	120.6(2)	C11	C12	C13	131.1(3)
O4	C19	C20	111.5(2)	C15A	C14A	C13	110.5(7)
O5	C19	O4	121.8(2)	C13	C14B	C15B	121.0(6)
O5	C19	C20	126.7(2)				

Table S15: Torsion Angles in ° for compound **5**

A	B	C	D	Angle/°	A	B	C	D	Angle/°
Cl1	C5	C4A	C8A	176.51(17)	C8A	C4A	C4	C3	2.2(3)
Cl1	C5	C4A	C4	-0.4(3)	C19	O4	C7	C6	-64.4(2)
O2	C6	C5	Cl1	-3.7(3)	C19	O4	C7	C8	64.5(2)
O2	C6	C5	C4A	171.6(2)	C19	O4	C7	C18	-179.63(19)
O2	C6	C7	O4	-34.6(3)	C4	C4A	C8A	C8	175.9(2)
O2	C6	C7	C8	-159.4(2)	C4	C4A	C8A	C1	-2.3(3)
O2	C6	C7	C18	80.1(3)	C4	C3	C9	C10	-16.9(5)
O3	C8	C8A	C4A	-169.3(2)	C1'	N1	C3	C4	178.1(2)
O3	C8	C8A	C1	8.9(3)	C1'	N1	C3	C9	-2.8(4)
O3	C8	C7	O4	32.5(3)	C1'	N1	C1	C8A	-178.3(2)
O3	C8	C7	C6	157.6(2)	C7	O4	C19	O5	-0.8(3)
O3	C8	C7	C18	-82.3(3)	C7	O4	C19	C20	178.75(19)
N1	C3	C4	C4A	-0.6(4)	C7	C6	C5	Cl1	170.99(16)
N1	C3	C9	C10	164.0(3)	C7	C6	C5	C4A	-13.7(3)
N1	C1	C8A	C8	-177.3(2)	C7	C8	C8A	C4A	13.6(3)
N1	C1	C8A	C4A	0.9(3)	C7	C8	C8A	C1	-168.2(2)
N1	C1'	C2'	O1'	-61.1(3)	C10	C11	C12	C13	177.1(4)
C6	C5	C4A	C8A	1.3(3)	C9	C3	C4	C4A	-179.7(2)
C6	C5	C4A	C4	-175.5(2)	C9	C10	C11	C12	-177.4(4)
C5	C6	C7	O4	150.2(2)	C9	C10	C11	C17A	-24.4(7)
C5	C6	C7	C8	25.5(3)	C9	C10	C11	C17B	27.8(5)
C5	C6	C7	C18	-95.1(2)	C11	C10	C9	C3	176.6(3)
C5	C4A	C8A	C8	-1.3(3)	C12	C13	C14A	C15A	59.8(7)
C5	C4A	C8A	C1	-179.4(2)	C12	C13	C14B	C15B	59.8(8)
C5	C4A	C4	C3	179.2(2)	C14A	C13	C12	C11	-138.7(5)
C3	N1	C1	C8A	0.8(3)	C17A	C11	C12	C13	23.4(7)
C3	N1	C1'	C2'	97.6(3)	C17B	C11	C12	C13	-31.6(7)
C1	N1	C3	C4	-1.0(3)	C14B	C13	C12	C11	-79.3(6)
C1	N1	C3	C9	178.2(2)	C16B	C13	C12	C11	144.8(6)
C1	N1	C1'	C2'	-83.3(3)	C16B	C13	C14B	C15B	-171.2(8)
C8A	C8	C7	O4	-150.35(18)	C16A	C13	C12	C11	107.6(7)
C8A	C8	C7	C6	-25.2(3)	C16A	C13	C14A	C15A	-178.2(7)
C8A	C8	C7	C18	94.8(2)					

Table S16: Hydrogen Bonds for compound **5**.

D	H	A	d(D-H)/Å	d(H-A)/Å	d(D-A)/Å	D-H-A/°
C1	H1	O5[1]	0.95	2.45	3.315(3)	150.6
C20	H20A	O3[2]	0.98	2.66	3.274(3)	121.3
C20	H20C	O5[3]	0.98	2.52	3.454(3)	159.8

[1]1+X,+Y,+Z; [2]-1+X,+Y,+Z; [3]-X,-1/2+Y,3/2-Z

Table S17 Hydrogen fractional atomic coordinates (×104) and equivalent isotropic displacement parameters (Å2×103) for compound **5**. Ueq is defined as 1/3 of the trace of the orthogonalised Uij.

**Atom**	**x**	**y**	**z**	**$U_{(eq)}$**
H1'	6832	8705	6782	55
H1	5801	3313	6959	28
H4	2000	4960	6053	33
H1'A	8218	6093	6488	35
H1'B	8537	4724	6759	35
H20A	-3417	-185	7472	47
H20B	-2701	1227	7709	47
H20C	-1387	-339	7685	47
H10	3625	6414	5722	45
H9	6208	7365	6220	50
H18A	2736	-1475	6804	49
H18B	2750	-572	6451	49
H18C	848	-1605	6557	49
H12	3294	7887	5258	68
H15A	528	9946	5377	150
H15B	-587	11179	5138	150
H15C	1335	11708	5351	150
H14A	2117	11226	4768	67
H14B	1523	9425	4825	67
H17A	4916	10153	5973	88
H17B	5858	10751	5625	88
H17C	7169	9692	5874	88
H17D	7345	9532	5934	69
H17E	7754	9863	5543	69
H17F	8269	8184	5701	69
H14C	1791	11199	5149	67
H14D	3454	12202	4960	67
H15D	2747	13320	5476	116
H15E	5027	12730	5477	116
H15F	3337	11732	5667	116
H16A	5143	8402	4743	92
H16B	4524	10051	4581	92
H16C	2832	8906	4727	92
H16D	6979	9595	4795	97
H16E	5239	10396	4577	97
H16F	5117	8570	4667	97
H2'A	8750(40)	7080(30)	7020(7)	22(6)
H2'B	6660(50)	6230(40)	7157(9)	43(9)
H13	5630(100)	10500(80)	5123(16)	120(20)

Figure S122: Evolution of retention time of acylated azaphilone in function of the length of acylation. Chromatograms are normalized to the most intense peak for each unique *m/z*.

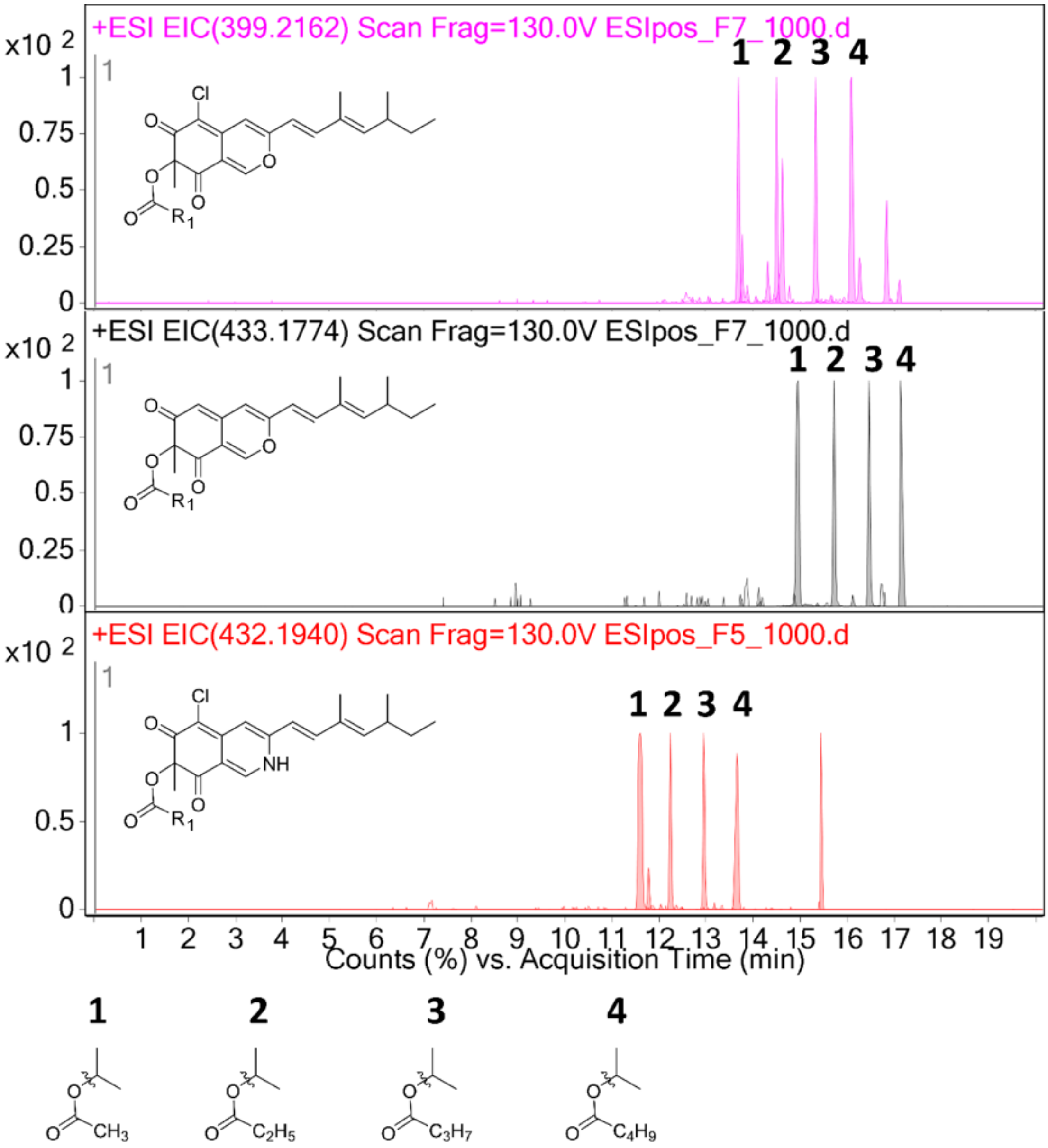

References:

1. Son, S.; Ko, S.-K.; Kim, J. W.; Lee, J. K.; Jang, M.; Ryoo, I.-J.; Hwang, G. J.; Kwon, M. C.; Shin, K.-S.; Futamura, Y.; Hong, Y.-S.; Oh, H.; Kim, B. Y.; Ueki, M.; Takahashi, S.; Osada, H.; Jang, J.-H.; Ahn, J. S. Structures and Biological Activities of Azaphilones Produced by Penicillium Sp. KCB11A109 from a Ginseng Field. *Phytochemistry* **2016**, *122*, 154–164. https://doi.org/10.1016/j.phytochem.2015.12.008.

2. Hemtasin, C.; Kanokmedhakul, S.; Moosophon, P.; Soytong, K.; Kanokmedhakul, K. Bioactive Azaphilones from the Fungus Penicillium Multicolor CM01. *Phytochem. Lett.* **2016**, *16*, 56–60. https://doi.org/10.1016/j.phytol.2016.03.004.

3. Chong, R.; King, R. R.; Whalley, W. B. The Chemistry of Fungi. Part L X P The Synthesis of (+)-Sclerotiorin, of (+)-4,6-Dimethylocta-Trans-2,Trans-4-Dienoic Acid, and of an Analogue of Rotiorin. *J. Chem. Soc*. **1971**, 3566–3571. https://doi.org/10.1039/J39710003566.

4. Gu, B.-B. Azaphilone and Isocoumarin Derivatives from the Sponge-Derived Fungus Eupenicillium Sp. 6A-9. *Tetrahedron Lett.* **2018**, *59* (36), 3345–3348. https://doi.org/10.1016/j.tetlet.2018.06.057
